# An SMS chatbot digital educational program to increase healthy eating behaviors in adolescence: A multifactorial randomized controlled trial among 7,890 participants in the Danish National Birth Cohort

**DOI:** 10.1371/journal.pmed.1004383

**Published:** 2024-06-14

**Authors:** Anne Ahrendt Bjerregaard, Daniel E. Zoughbie, Jørgen Vinsløv Hansen, Charlotta Granström, Marin Strøm, Þórhallur Ingi Halldórsson, Inger Kristine Meder, Walter Churchill Willett, Eric L. Ding, Sjúrður Fróði Olsen

**Affiliations:** 1 Department of Epidemiology Research, Statens Serum Institut, Copenhagen, Denmark; 2 Center for Clinical Research and Prevention, Copenhagen University Hospital – Bispebjerg and Frederiksberg Hospital, Copenhagen, Denmark; 3 University of California, Berkeley, California, United States of America; 4 New England Institute for Complex Systems, Cambridge, Massachusetts, United States of America; 5 University of the Faroe Islands, Tórshavn, Faroe Islands; 6 Faculty of food science and nutrition, University of Iceland, Reykjavík, Iceland; 7 Secretariat of the Danish National Birth Cohort, Statens Serum Institut, Copenhagen, Denmark; 8 Department of Nutrition, Harvard TH Chan School of Public Health (for ELD: affiliation at time of project), Boston, Massachusetts, United States of America; 9 Department of Public Health, University of Copenhagen, Copenhagen, Denmark; University of Cambridge, UNITED KINGDOM

## Abstract

**Background:**

Few cost-effective strategies to shift dietary habits of populations in a healthier direction have been identified. We examined if participating in a chatbot health education program transmitted by Short Messages Service (“SMS-program”) could improve adolescent dietary behaviors and body weight trajectories. We also explored possible added effects of maternal or peer involvement.

**Methods and findings:**

We conducted a randomized controlled trial (RCT) among adolescents from the Danish National Birth Cohort (DNBC). Eligible were adolescents who during 2015 to 2016 at age 14 years had completed a questionnaire assessing height, weight, and dietary habits. Two thirds were offered participation in an SMS-program, whereas 1/3 (“non-SMS group”) received no offer. The SMS program aimed to improve 3 key dietary intake behaviors: sugar-sweetened beverages (SSBs), fruit and vegetables (FV), and fish. The offered programs had 3 factorially randomized schemes; the aims of these were to test effect of asking the mother or a friend to also participate in the health promotion program, and to test the effect of a 4-week individually tailored SMS program against the full 12-week SMS program targeting all 3 dietary factors.

Height and weight and intakes of SSB, FV, and fish were assessed twice by a smartphone-based abbreviated dietary questionnaire completed at 6 months (m) and 18 m follow-up. Main outcome measures were (1) body mass index (BMI) z-score; and (2) an abbreviated Healthy Eating Index (mini-HEI, 1 m window, as mean of z-scores for SSB, FV, and fish).

Among the 7,890 randomized adolescents, 5,260 were assigned to any SMS program; 63% (3,338) joined the offered program. Among the 7,890 randomized, 74% (5,853) and 68% (5,370) responded to follow-ups at 6 m and 18 m, respectively. Effects were estimated by intention-to-treat (ITT) analyses and inverse probability weighted per-protocol (IPW-PP) analyses excluding adolescents who did not join the program.

Mean (standard deviation (SD)) mini-HEI at baseline, 6 m and 18 m was −0.01 (0.64), 0.01 (0.59), and −0.01 (0.59), respectively. In ITT-analyses, no effects were observed, at any time point, in those who had received any SMS program compared to the non-SMS group, on BMI z-score (6 m: −0.010 [95% confidence interval (CI) −0.035, 0.015]; *p* = 0.442, 18 m: 0.002 [95% CI −0.029, 0.033]; *p* = 0.901) or mini-HEI (6 m: 0.016 [95% CI −0.011, 0.043]; *p* = 0.253, 18m: −0.016 [95% CI −0.045, 0.013]; *p* = 0.286).

In IPW-PP analyses, at 6 m, a small decrease in BMI z-score (−0.030 [95% CI −0.057, −0.003]; *p* = 0.032) was observed, whereas no significant effect was observed in mini-HEI (0.027 [95% CI −0.002, 0.056]; *p* = 0.072), among those who had received any SMS program compared to the non-SMS group. At 18 m, no associations were observed (BMI z-score: −0.006 [95% CI −0.039, 0.027]; *p* = 0.724, and mini-HEI: −0.005 [95% CI −0.036, 0.026]; *p* = 0.755).

The main limitations of the study were that DNBC participants, though derived from the general population, tend to have higher socioeconomic status than average, and that outcome measures were self-reported.

**Conclusions:**

In this study, a chatbot health education program delivered through an SMS program had no effect on dietary habits or weight trajectories in ITT analyses. However, IPW-PP-analyses, based on those 63% who had joined the offered SMS program, suggested modest improvements in weight development at 6 m, which had faded at 18 m. Future research should focus on developing gender-specific messaging programs including “booster” messages to obtain sustained engagement.

**Clinical Trial Registration:**

ClinicalTrials.gov Identifier: NCT02809196 https://clinicaltrials.gov/study/NCT02809196.

## Abstract 2

Anne Ahrendt Bjerregaard and team examine if participating in a chatbot health education program could improve adolescent dietary behaviors and body weight trajectories.

## Author summary

**Why was this study done?:**

- Poor nutrition accounts for substantial proportions of occurrences of ill-health and mortality in modern societies and improving dietary habits in the general population is therefore a public health priority.
- Early adolescence would seem an ideal age to provide health promotion programs.
- Use of Short Messages Service (SMS) technology allows easy connection with the adolescents and their social network and present unique possibilities for communicating with large numbers at a low cost.

**What did the researchers do and find?:**

- In the frame of the large population based Danish National Birth Cohort (DNBC), we developed and evaluated the efficacy of a novel SMS-based chatbot educational program aimed at improving adolescent dietary behaviors and body weight trajectories at age 14 years.
- We compared adolescents who were offered a chatbot education program with adolescents who were not offered a chatbot education program. We found no clear effect on healthier dietary habits or different body weight trajectories at 6 and 18 months after project initiation.
- Analyses based on the 63% who enrolled in the SMS program offered to them suggested modest improvements in weight change and diet quality at 6 months in males only, which faded by 18 months.

**What do these findings mean?:**

- Our study documented high feasibility and 63% acceptability in young adolescents of an artificially intelligent digital educational program lasting up to 12 weeks.
- The educational program, designed to improve adolescent dietary behaviors and body weight trajectories, but as implemented here, could not document an effect of offering an SMS-based educational program to adolescents on body mass index (BMI) or dietary quality.
- Limitations include that outcome measures were self-reported.
- Future research should focus on developing gender-specific messaging programs including “booster” messages to obtain sustained engagement.

## Introduction

Poor nutrition accounts for substantial proportions of occurrences of ill-health and mortality in modern societies [1]. Improving dietary habits in the general population is therefore a public health priority. Various campaigns, taxes on foods (e.g., in Denmark, there are taxes on certain sugar containing food), or food-based dietary recommendations are some of the tools used by health authorities to influence food habits of the population in positive directions [2–4]. However, it has proved difficult to develop health promotion programs that efficiently bring about lasting changes towards healthier dietary habits and are sufficiently low-cost to be applied to large numbers of subjects [5,6]. A 2019 Cochrane review of 31 randomized controlled trials (RCTs) testing the effectiveness of a range of interventions designed to prevent obesity in adolescents by targeting dietary or physical activity habits, found limited effective interventions for adolescents aged 13 to 18 years old [7].

Early adolescence would seem an ideal age for such programs [8,9]. During adolescence, the individual becomes more independent with respect to many matters including lifestyle choices [10,11]. Smartphone technologies are widely used messaging platforms among adolescents [12,13] and present unique possibilities for communicating with large numbers at a low cost. Use of Short Messages Service (SMS) technology also allows easy connection with the adolescents’ social network, which in itself carries important but understudied possible avenues for metabolic improvement [14–17]. In particular, there is a paucity of data concerning the extent to which social network activation—in the context of both peers and familial forces—can positively influence critical adolescent behaviors, such as diet, throughout various stages of adolescence and into adulthood [18–21].

To address these gaps, we developed and evaluated the efficacy of a novel SMS-based chatbot educational program aimed at improving adolescent dietary behaviors and body weight trajectories. We undertook the study in the frame of the large population-based Danish National Birth Cohort (DNBC) [22–24], which follows the participants from first trimester of fetal life through childhood and adolescence into adulthood and therefore is uniquely suited for testing short- and long-term effects of health promotional programs. A comprehensive questionnaire completed online at 14 years [24,25] constituted the baseline for evaluating behavioral and height and weight change during adolescence.

Due to pedagogical and logistical constraints, when using SMS, we focused on 3 key dietary behaviors important to various aspects of the adolescents’ health: sugar-sweetened beverages (SSB), fruits and vegetables (FV), and fish. High intake of SSB has been associated with increased risk of obesity, chronic heart diseases, and dental decay [26–28], and a 2019 Cochrane review called for more research into effective, scalable interventions addressing SSB consumption using methodologically rigorous evaluations to strengthen the existing evidence base [29,30]. Similarly, low intakes of FV and of fish have been associated with increased risk of chronic heart diseases and ischemic stroke [31,32], and fish intake has been directly associated with optimal growth and development [33]. The majority of Danish children do not comply with current recommendations for optimal intakes of SSB, FV, and fish [34,35].

The present study was an RCT among 7,890 adolescent DNBC participants, which aimed to examine if changes towards healthier dietary habits and weight trajectories can be detected at 6 and 18 months follow up after the initiation of an SMS-transmitted health education program targeting these 3 key dietary behaviors. The study was, by factorial randomization generating variants of the offered programs, designed to also enable assessing possibly added effects of maternal or peer involvement as well as of targeting the educational content to the adolescent’s individual risk profile. Here, we first report on the overall effects on adolescent dietary behaviors and body weight trajectories of participating in any variant of the offered SMS educational programs, as compared to a control group who were not offered participation in any program. We furthermore report findings regarding; asking the mother versus not asking the mother of the adolescent to participate in a corresponding SMS-transmitted health promotion program summing up main points from the adolescent’s program; asking a friend versus not asking a friend of the adolescent to participate in the same SMS-transmitted program; and tailoring versus not tailoring the SMS-transmitted program to the adolescent’s risk profile by targeting a specific dietary factor.

## Methods and materials

### The Danish National Birth Cohort

Between 1996 and 2002, 91,796 pregnant women were enrolled in the DNBC and contributed with 101,033 births. At enrolment in the DNBC, mothers had given informed consent on behalf of their children. The women were interviewed by telephone twice in pregnancy, and around week 25 of gestation they completed a 360-item food frequency questionnaire (FFQ) [22,23]. After giving birth, regular follow-ups have been conducted at child ages 0.5, 1.5, 7, 11, and 14 years, where mothers acted as gatekeepers when the children were invited to participate in surveys; the follow-up at 14 years served as recruitment platform for the present study. The DNBC was conducted according to the guidelines laid out in the Declaration of Helsinki and all procedures involving human participants were approved by the National Committee on Health Research Ethics in Denmark (H-4-2011-045). The protocol of the present project was approved by the DNBC Management Group and Steering Committee (ref. no. 2014–01). We furthermore submitted an outline of the protocol (S3 Text) to The Scientific Ethical Committee of the Capital Region of Denmark; the Committee classified the project as educational, not biomedical (H-4-2014-FSP), and thus waived the need for committee consent. An outline of the protocol was also submitted for review by the Institutional Review Board at Harvard School of Public Health, Boston, Massachusetts, United States of America (IRB16-0827); we did this because some members of the project team were affiliated with Harvard School of Public Health, Boston, Massachusetts, but since these persons would not have access to data, the Institutional Review Board at Harvard School of Public Health also waived the need for their IRB consent. The project was furthermore approved by the Danish Data Protection Agency (2014-41-3277) and later by SSI’s Department of Data Protection and Information Security (6-12-2020). This study is reported as per the Consolidated Standards of Reporting Trials (CONSORT) guideline for RCTs (see S1 CONSORT Checklist). Further ethical considerations are provided in S1 Text.

Adolescents invited to participate in the SMS educational program were recruited from those DNBC children who had completed the 14-year follow up. The data collection at this occasion included an online comprehensive FFQ component [17], which is here designated “The baseline FFQ,” and which represented dietary intake in the previous year. The baseline FFQ asked about frequency of intake of 158 food items covering main food groups such as beverages, dairy, bread, and cereals, as well as butter on bread, spread on bread, cold and warm dishes, side dishes and condiments, fruits and vegetables, snacks, and desserts. Additionally, there were questions on height and weight, meal patterns, use of dietary supplements, and physical activity. The height and weight questions were worded as follows: “How tall are you? Enter your height in centimeters (whole numbers) and What is your weight? Enter your weight in kilograms (whole numbers).” An English translation of the questionnaire is available in Supporting information in [24].

### Dietary targets and ethical considerations in the SMS educational program

Prior to settling on specific target dietary factors for the SMS educational program, we carefully evaluated the literature. We deemed evidence to be strong that, for the majority of Danish adolescents, a health educational program aiming to reduce intake of SSB, increase intake of FV, and increase intake of fish could be beneficial to health—and that it, at worst, would do no harm to the health of Danish adolescents (see S1 Text, the Section “Ethical and scientific considerations underlying the choice of the program’s three target factors: Lowering the intake of sugar sweetened beverages, increasing the intake of fruit and vegetables, and increasing the intake of fish”).

To enable testing the effect of delivering an SMS educational program targeting individual risk profiles, the adolescents were, prior to nomination for a program, stratified based on risk profiles. The strata were defined by the reported information on dietary intake relative to recommended levels of fish, SSB, and FV intake. This was feasible because the baseline FFQ was completed online, allowing automatized processing of the entered information. Four exhaustive and mutually exclusive strata (Dietary Risk Strata I–IV) with different risk profiles with respect to intake of fish, FV, and SSB were created; a description of the algorithms for defining each stratum is provided in S1 Text (see the Section “Definition of pre-randomization strata”).

### Exposures and variants of the SMS educational program

The study was designed to enable estimation of the overall effect of participating with any variant of the SMS educational program, as well as estimation of the modifying effects of 3 dichotomous exposures that defined the (2 × 2 × 2) 8 variants of the SMS educational program described in the next section on Randomization. The first modifying exposure was that the program could simultaneously involve the adolescent’s friend or not, the second that it could simultaneously involve the adolescent’s mother or not. The third modifying exposure was that the program could either address all three dietary factors (SSB, FV, and fish) (FULL program), or only one of these (SSB, FV, or fish) selected based on the adolescent’s individual risk profile as judged from the adolescent’s online report in the baseline FFQ (please confer Dietary Risk Strata defined above).

### Randomization scheme and hypotheses

The nomination of an adolescent for a particular program took place by a process that corresponded to a “2 × 2 × 2 factorial plus 1 (controls)” design RCT, creating 9 randomization arms, A1–A9; see Table 1. Computer-generated randomization supervised by an independent programmer (KA) was done within 24 blocks defined by the 2 sexes, 3 age groups, and 4 baseline dietary strata (the “Dietary Risk Strata”) to ensure that the arms were balanced with respect to sex, age, and risk profile. Adolescents ending up in the first 8 arms, A1–A8, were offered participation in one of the 8 variants of the program, whereas those ending up in arm A9 were not offered to participate in any of the 8 SMS-based educational programs. Arm A9 was used as a control group representing development over time in lifestyle habits and in body mass index (BMI) when no SMS program was offered. Therefore arm A9 was designed to be 4 times larger than each of the first 8 arms (A1–A8), which were all designed to be of equal size; in other words, the ratios between the sizes of the 9 randomization arms were A1:A2:A3:A4:A5:A6:A7:A8:A9 = 1:1:1:1:1:1:1:1:4. In the following, arm A9 is referred to as the “non-SMS group” and arms A1–A8 are referred to as those who received any SMS program. Thus, within the group of adolescents offered participation in the SMS programs there was a separate 2 × 2 × 2 factorial trial.

**Table 1:** Randomization scheme for a “2 × 2 × 2 factorial plus 1 (controls)” design trial, generating 9 arms.

Arms:	A1	A2	A3	A4	A5	A6	A7	A8	A9 (controls) (non-SMS)
**SMS:**	+SMS	+SMS	+SMS	+SMS	+SMS	+SMS	+SMS	+SMS	-SMS
**Friend:**	+F	+F	+F	+F	-F	-F	-F	-F	N.A.
**Mother:**	+M	+M	-M	-M	+M	+M	-M	-M	N.A.
**Targets:**	3 DFs	1 DF	3 DFs	1 DF	3 DFs	1 DF	3 DFs	1 DF	N.A.

SMS = Short Messages Service; +SMS = Offered to participate in an SMS educational program; -SMS = Not offered to participate in an SMS educational program; +F = Involving a friend; -F = Not involving a friend; +M = Involving the mother; -M = Not involving the mother; 3 DFs = FULL program, addressing all 3 target dietary factors, irrespective of which *Dietary Risk Stratum* the adolescent belonged to; 1 DF = Targeted program, addressing only 1 dietary factor, tailored corresponding to which *Dietary Risk Stratum* the adolescent belonged to (see manuscript text and Supporting information); N.A. = Not Applicable.

In this report, we tested 4 overarching hypotheses defined a priori, see pages 3 and 4 in S2 Text (Statistical Analysis Plan, SAP): Hypothesis (1) Any SMS educational program versus no SMS-effect, where we examined if any of the SMS programs together are superior to a fully null group without any SMS or active education (aggregated arms A1–A8 compared to arm A9). Hypothesis (2) Mother versus no mother-effect (+M,-M), where we examined if an adolescent receiving a standard SMS dietary education program that is reinforced by inviting the mother of the participant to also concurrently participate in a corresponding SMS-transmitted health promotion program summing up main points from the adolescents program is more effective than a program not reinforced by a mother on effects of the specific outcomes (arms A1+A2+A5+A6 compared to arms A3+A4+A7+A8). Hypothesis (3) Friend versus no friend-effect (+F,-F), where we examined if an adolescent receiving a standard SMS dietary education program that is reinforced by inviting a friend of the participant to also concurrently participate in a corresponding SMS-transmitted health promotion program is more effective than a program not reinforced by a friend on effects of the specific outcomes (arms A1+A2+A3+A4 compared to arms A5+A6+A7+A8). Hypothesis (4) SMS tailored versus no tailored (FULL) educational program-effect, where we examined if an adolescent receiving an SMS education program that targets a specific dietary factor, tailored to match the adolescents’ individual risk profiles, is as effective as compared to an SMS education program generically designed to target a standard mixed education of various dietary factor (arms A1+A3+A5+A7 compared to arms A2+A4+A6+A8).

### Eligibility screening and invitation procedure

Eligible for our SMS educational program were those who during 6 months (m) from early summer 2016, in 3 age groups at ages 14 years and 3 m (number assessed for eligibility = 9,181 and number found eligible = 2,614), 14 years and 9 m (10,146 and 2,654, respectively), and 15 years and 3 m (10,178 and 2,622, respectively), had completed the baseline FFQ; where the mother had completed the pregnancy FFQ and participated in both pregnancy interview 1 and 2; and where mother and child at the time of recruitment were living at the same address. The time schedule for sending invitations in the 3 age strata is depicted in S1 Fig.

The assessment of eligibility and the subsequent randomization were done by an automatized procedure. Provided that the adolescent was deemed eligible according to the above criteria, and provided that randomization had allocated him or her to be offered participation in an SMS program (corresponding to randomization arms A1–A8, see above), a personal invitation letter with instructions on how to join the SMS program was generated. Adolescents deemed eligible, but allocated to the non-SMS group (Arm 9 below), were not contacted at this stage. Invitation letters were packed manually and sent out by postal mail approximately once a week. The invitees could call in and speak to a person at the project secretariat if they had any questions regarding participation in the program.

**Fig 1:**
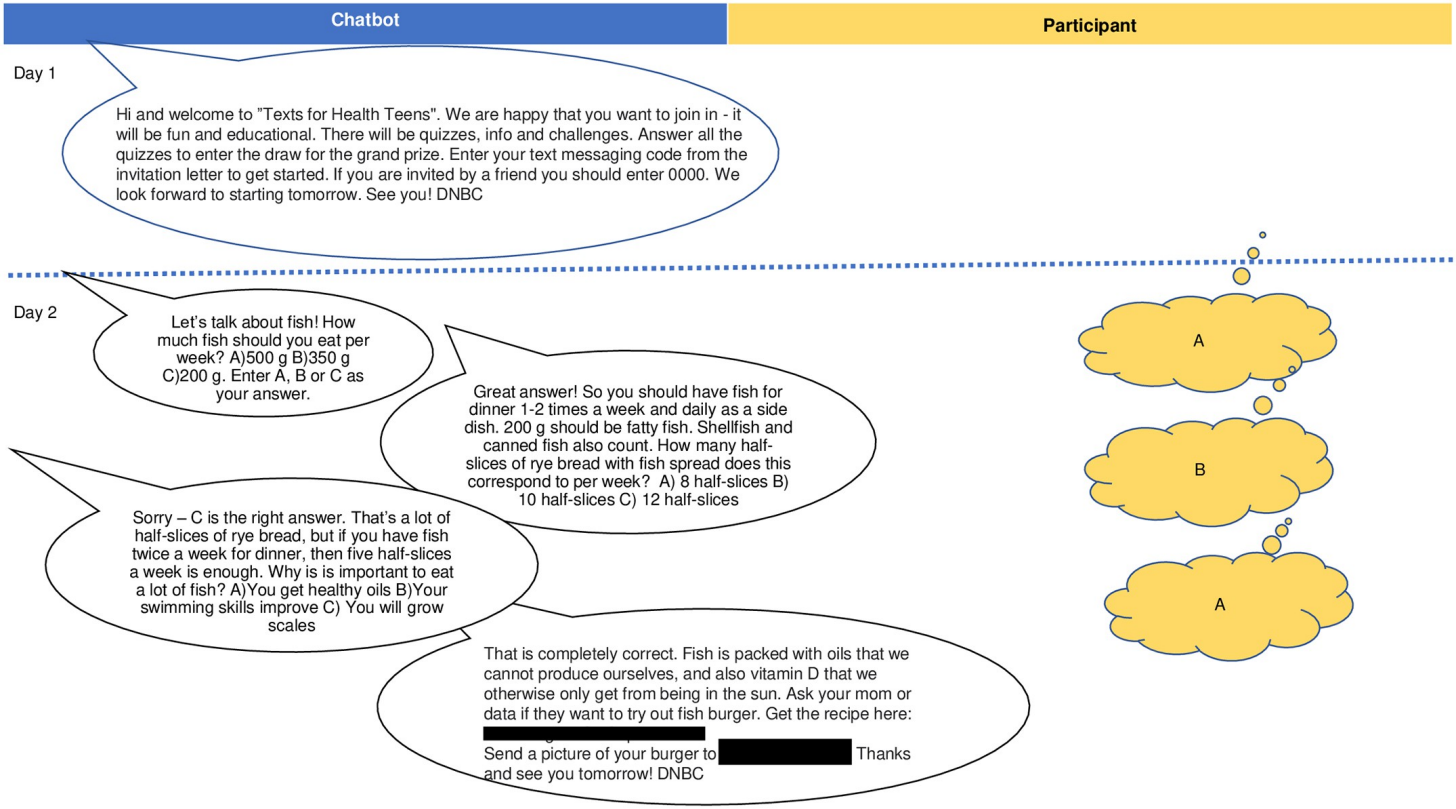
Chatbot cartoon. Example of two-way messaging in the chatbot. The structure and content of the SMS messaging programs are described in more detail in S1 Text (see the Section “Duration, structure and content of the SMS messaging programs”) and S2 Fig: Example of an SMS script from Day 3. SMS, Short Messages Service.

All SMS messages were sent through an international telecommunications carrier. To accept the invitation to participate in the nominated SMS educational program, each adolescent would need to SMS the word JOIN to a specific phone number stated in the invitation he or she had received. The adolescent was then automatically enrolled in the program and received SMS messages that he or she could reply to, which would in turn generate automatic responses, according to a pre-programmed algorithm. Once these steps had been taken, each person (index adolescents, friends, mothers) enrolled in the program continued to receive information and was asked questions on an almost daily basis for the duration of the program, which was 12 weeks for the FULL program and 4 weeks for each of the 3 targeted SMS programs; the latter were targeted towards increasing the adolescents’ fish intake, increasing their FV intake, or reducing their SSB intake. Thus, the main purpose of text messages was education by communicating overall dietary recommendations, as well as recommendations on fish, FV, and/or SSB (depending on the program) to the participants in a manner that would be appealing to the age group. There was no guideline on who to invite as a friend. Messages were grouped according to type of message, for example, quizzes, meal, specific food item, etc., with most of the messages being quizzes to keep the participants engaged in the intervention. The messages were designed in a way such that the answer to a question was put together with a new question, tip, challenge, or reminder. The FULL program consisted of 188 quiz/tips/reminder messages: 23 messages on SSB, 26 messages on FV, 24 messages on fish, 19 messages on physical activity, 3 messages on dietary fibers, and 20 messages about meals (referring to snacking, breakfast, lunch, and distribution of macronutrients in a meal). Each of the 3 targeted SMS programs constituted part of the FULL program messages. All messages were developed by skilled nutritional scientists in collaboration with a communication expert. Content of messages was discussed in-house among scientific colleagues followed by a test of acceptability at 3 different occasions among Danish school children. An example of a two-way communication is shown in the cartoon in Fig 1.

### Incentives to keep participants motivated

An online portal was created particularly for the project on the DNBC website to provide educational resources and other information for the participants. An Instagram site was established, and participants were encouraged to share photos of healthy meals, packed lunches, snacks, physical activity, or the like. Educational information on healthy diet and lifestyle would be presented in a way that had appeal to the young people; it could include competitions about suggesting (the most funny, exciting, exotic, etc.) healthy recipes, where they could win gift cards to amusement parks, gift cards to a selection of shops, or cinema tickets.

### Outcome assessments at 6 and 18 months

All adolescents were asked to complete an FFQ again twice, 7 and 19 m after they had completed the baseline FFQ. Because those who were invited to the SMS programs joined the programs on average 4 weeks after they had completed the baseline FFQ, this corresponded, on average, to 6 and 18 m after commencement of the SMS programs. The follow-ups 7 and 19 m after they had completed the baseline FFQ were thus named the 6 m and the 18 m follow-ups.

To optimize follow-up response rates, the follow-up FFQ was a shortened version of the full baseline FFQ. It assessed frequencies of dietary intake from the previous month and comprised 40 food items covering consumption of food relevant for the SMS educational program. Questions on height and weight, meal patterns, and physical activity were kept identical to the corresponding questions in the baseline FFQ. Frequencies of SSB, FV, and fish intake were assessed with 5 versus 27, 5 versus 5, and 2 versus 6 questions in the follow-up versus the baseline questionnaire, respectively. The height and weight questions were worded similarly as in the Baseline Questionnaire, i.e.: “How tall are you? Enter your height in centimeters (whole numbers) and What is your weight? Enter your weight in kilograms (whole numbers).” For all questionnaire versions frequencies of items were weighted according to predefined portion sizes so that SSB, FV, and fish scores could be interpreted as amounts consumed. Before launching the follow-up questionnaire, it was pretested in a subgroup of 90 adolescents from the non-SMS group (Arm A9). Here, we compared dietary intake assessed with the full FFQ completed at baseline with that assessed with the shorter follow-up FFQ 7 months later. Acceptable Spearman correlations coefficients were observed for SSB (*r* = 0.58), FV (*r* = 0.47), and fish (*r* = 0.66). To further optimize the follow-up response rate, we included a video clip with an encouraging statement from a well-known Danish youtuber in the invitations to complete the follow-up questionnaire, which were sent to the adolescents’ cell phones.

Two main outcomes were defined a priori, to address changes toward healthier dietary behaviors in general and toward healthier weight trajectories, respectively. Main Outcome 1, corresponding to Primary Hypothesis 1 in the a priori SAP (see page 7 in S2 Text), was defined by a “mini Healthy Eating Index” (mini-HEI) to evaluate dietary behaviors. The mini-HEI was based on assessments of intake of the 3 target diets: FV, SSB, and fish. We calculated the average of z-scores (using means and standard deviations from the non-SMS group) of the 3 log-transformed dietary factors. Adolescents reporting zero intake had their measure replaced by the smallest positive intake reported among all other children at the same time point, before taking the logarithm. We considered low SSB, high FV, and high fish intake as healthy eating. Thus, the mini-HEI was calculated to be the FV z-score plus the fish z-score minus the SSB z-score and divided by 3.

To address changes in weight trajectories, a BMI z-score was defined as Main Outcome 2, corresponding to Primary Hypothesis 2 in the a priori SAP (see page 7 in S2 Text). Height and weight were self-reported in both the baseline FFQ and the 2 follow-up FFQs at 6 m and 18 m and used for BMI calculation at the 3 time points. The BMI z-score was based on the standards published by Centers for Disease Control and Prevention (CDC) [36,37] and was calculated similarly at baseline and follow-up. Twelve adolescents were excluded from the analyses of BMI as 2 had no height and weight observations at baseline and 10 were considered outliers on these measures (more than 40 kilos or 40 centimeters difference between observations at different time points).

In addition to the 2 main outcomes, we estimated the separate effects on the 3 log-transformed values of the dietary factors (SSB, FV, and fish) and on 2 dichotomized versions of the BMI z-score using the 85th and 95th percentile of the standard normal distribution cutoff to define overweight and obesity, respectively. Again, the standards published by CDC were used for defining overweight and obesity [37].

### Statistical analyses

A detailed SAP was prepared prior to conducting the analysis and filed at the Office of Research Integrity, Statens Serum Institut, and is available as S2 Text “Statistical Analysis Plan (SAP) for Texts For Healthy Teens: A Health Education Program for Adolescents (T4HT).”

For the mini-HEI and BMI z-score, the effect of the program was assessed at 6 m, at 18 m, and at both points in time by a joint test.

#### Defining participation and calculating propensities for participation

We estimated effects in intention-to-treat (ITT) analyses as well as among those who were actively participating as per-protocol (PP) analyses. The latter excluded adolescents who did not text “JOIN” in their response to the invitation to participate in an SMS program.

In analyses of effect restricted to those who did participate, we accounted for a set of factors which may determine participation in the offered SMS programs. We used a modified version of the propensity score-based method of Stuart and Jo [38] to weigh observations of participants in each arm to have similar characteristics as the full population assigned to that arm. For each of the 4 exposures (getting any SMS, involving mother, involving friend, 1 versus 3 targeted dietary factors) propensities for participation given a set of predictors were calculated by 2 logistic regressions; one for the exposed group and one for the reference group corresponding to the specific exposure. For the estimation of effect of any type of SMS program, there was no issue of nonparticipation in the reference (i.e., non-SMS) group; therefore, everyone in this group was assigned a propensity of 1.

The predictors of the logistic regression were a maternal pregnancy HEI score [24] (low, medium, high), smoking in pregnancy (yes, no), physical activity level in pregnancy (low, medium, high metabolic equivalents (METS) score), pre-pregnancy BMI (underweight, normal weight, overweight, obese), and participation in the following previous DNBC follow-up surveys: when the child was 6 m, 18 m, and 7 years, respectively (“yes” to all versus at least 1 “no”).

Given the values of its predictors, everyone thus had 2 propensities; one for participating had he/she been randomized to the exposed group, and one had he/she been randomized to the reference group. We divided the former by the latter and formed 10 groups by the deciles. Further, we calculated weights as the inverse of the propensity of participation of the individual given the actual exposure group to which the individual was randomized. In the calculation of propensities for participation in the SMS program, 3 binary variables defining if the adolescent was randomized to an arm where (1) the mother was involved; (2) a friend was involved; and (3) dietary factors were targeted, were also included to the logistic models.

#### Statistical models

The continuous outcomes were analyzed using normal generalized estimating equation (GEE) models with identity link function where the measurements at 6 and 18 m after commencement of the SMS program were the dependent variables.

For the ITT analyses, the effects of the exposure at the 2 time points (at 6 and 18 m) were adjusted for the corresponding value at baseline (linear effect) and its interaction with time as well as the full combination of sex, age group, and strata that determined randomization (factor with 24 levels). Repeated measures (at 6 m and 18 m, respectively) were accounted for in the joint test by modeling the 2 follow-up measurements from an individual to be correlated using an unstructured covariance matrix. When estimating the effect among participants, the same statistical model was used as for the ITT analyses except that observations were weighted by the inverse of the propensity of participation and the grouped propensity score was added to the set of covariates in the model. Estimates from the analyses are presented along with the empirical standard error estimates. Additional analyses among participants without weighting and propensity adjustment were also performed.

The binary outcomes were analyzed using Poisson GEE models with log link function allowing measurements from an individual to be correlated using an unstructured covariance matrix. The same set of weights and covariates were included as in the analyses of the continuous outcomes, except for the grouped propensity score and only additive effects of sex, age group, and baseline diet strata were adjusted for because the models did not converge with the interaction of these variables/covariates. Using empirical standard error estimates results are presented as risk ratios (RRs) with 95% confidence intervals (95% CIs). Using the approach of Stuart and Jo [38] supplementary analyses were made for estimating complier average causal effects (CACE) among participants of the offered program compared to the non-SMS group on the mini-Healthy Eating Index (HEI) and on the BMI z-scores; that is instead of weighting the adolescents in the SMS group by the inverse of their propensities to participate it was the adolescents in the non-SMS group that were weighted with their propensities of participation.

PROC GENMOD of SAS 9.4 were used for the analyses. Graphical display of results was made using R, version 3.5.1.

### Trial registration

The trial was uploaded at ClinicalTrial.gov with first submitted date on June 20, 2016, and with first posted date on June 22, 2016; the identifier is NCT02809196.

Activities in the trial according to calendar time are shown in S1 Fig. The first adolescent to join the offered SMS program did so on approximately June 1, 2016. The duration of the time period of sending out invitations was, by design, fixed and made to last for 6 m. The adolescents were split into 3 groups based on date of birth; January 2001 to June 2001, July 2001 to December 2001, and January 2002 to June 2002, respectively, so that they were invited in the week they reached the ages of 15 years and 3 m, 14 years and 9 m, and 14 years and 3 m for the oldest, middle and youngest age strata, respectively. With this design, where 3 age strata were invited consecutively and simultaneously, we were able to invite a large number of eligible adolescents to participate in the SMS educational program during the 6 m when invitations were sent out; see S1 Fig.

While the first follow-up at 6 m was conducted and completed as planned, we had to postpone the second follow-up, which (as stated in the preregistration protocol at ClinicalTrial.gov NCT02809196) was initially planned to take place at 12 m, to 18 m, because we for practical reasons were unable to have all logistics in place for a 12 m follow-up.

The SAP was mailed to, and filed at, the Office of Research Integrity, Statens Serum Institut, on June 17, 2019. This date preceded the date of opening the data (i.e., relating the outcome measures to information about which trial arms the adolescents had been randomly allocated to) to test the hypotheses of the present study. The SAP is available as S2 Text “Statistical Analysis Plan (SAP) for Texts For Healthy Teens: A Health Education Program for Adolescents (T4HT).”

## Results

**Fig 2:**
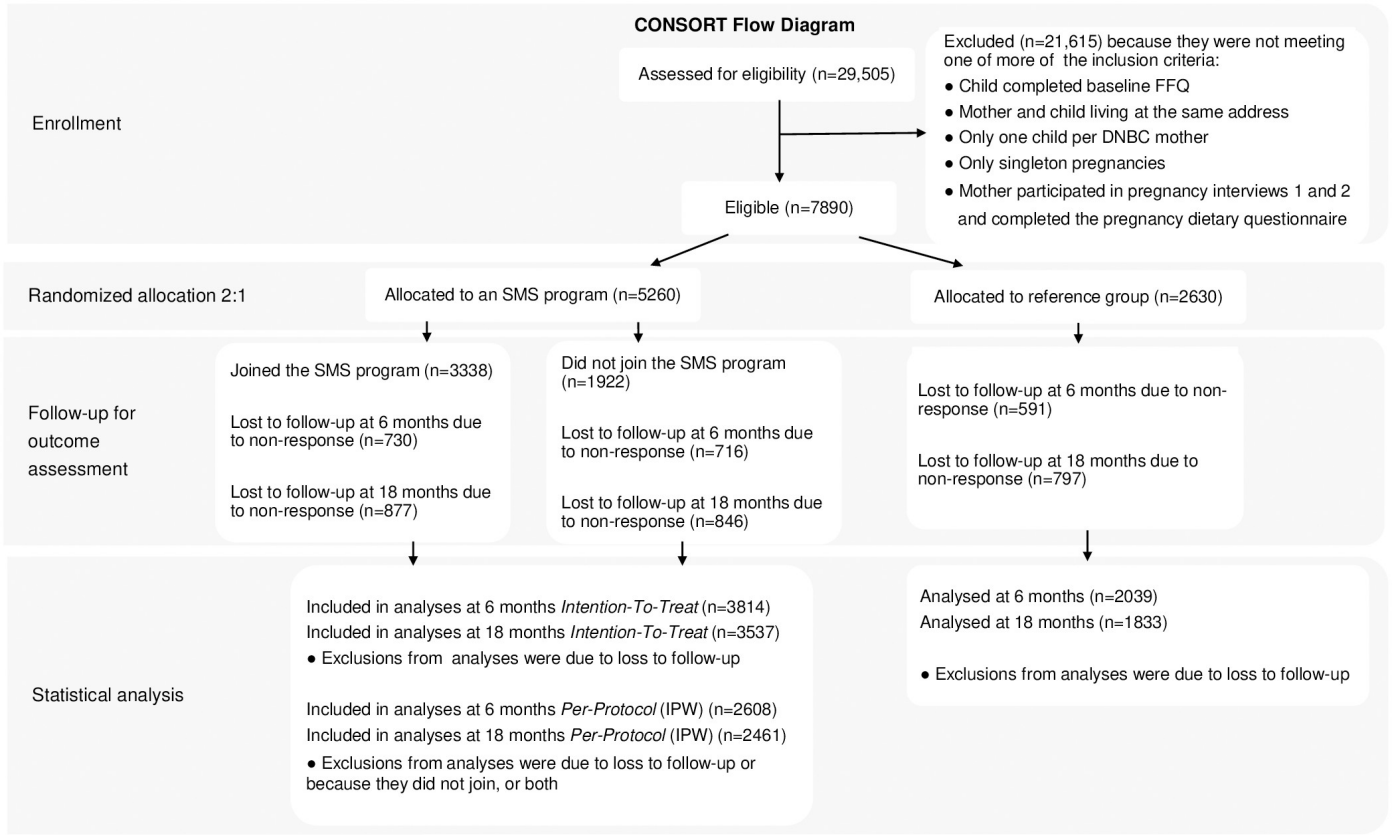
CONSORT Flow diagram. DNBC, Danish National Birth Cohort; FFQ, food frequency questionnaire; IPW, Inverse Probability Weighting; SMS, Short Messages Service.

A total of 7,890 adolescents were eligible and randomized of whom 5,260 were assigned to one of the 8 SMS program (arms A1–A8) and 2,630 were assigned to the non-SMS group (arm A9, controls). Among those assigned to an SMS program, 3,338 (63%) joined the program they had been offered. Among the 7,890 randomized, 5,853 (74%) responded to the follow-up FFQ at 6 months and 5,370 (68%) to the follow-up FFQ at 18 months (see CONSORT Flow diagram in Fig 2).

In Table 2, characteristics at baseline and predictors of participation in the offered SMS educational program are presented. As expected, distributions of variables at baseline were similar between comparison groups.

**Table 2:** Characteristics at baseline and mean of main outcomes at 6- and 18-months follow-up, according to whether or not the adolescents^g^ had been allocated to an SMS program, whether or not they joined the program, and whether or not those who joined had been allocated to a program implicating the mother, a friend, or a programmed that was tailored. Values are mean (SD) unless otherwise assigned.

						Randomization of joined adolescents					
	All eligible		Randomized to		Joined	Including Mother		Including Friend		Tailored	
			No SMS	Any SMS	Any SMS	No	Yes	No	Yes	No	Yes
Baseline											
characteristics											
n (denominator)		7,890	2,630	5,260	3,338	1,704	1,634	1,840	1,498	1,661	1,677
Adolescent HEI^a^		−0.01 (0.64)	0.00 (0.63)	−0.01 (0.65)	0.02 (0.63)	0.01 (0.64)	0.03 (0.62)	0.01 (0.64)	0.03 (0.63)	0.02 (0.63)	0.02 (0.64)
Adolescent BMIz^b^		−0.15 (0.93)	−0.16 (0.92)	−0.15 (0.94)	−0.15 (0.92)	−0.14 (0.90)	−0.15 (0.94)	−0.15 (0.91)	−0.14 (0.92)	−0.13 (0.94)	−0.16 (0.90)
	n	%	%	%	%	%	%	%	%	%	%
Maternal HEI^c^ (%)											
*High*	1,949	24.7	24.0	25.1	23.6	23.5	23.7	23.9	23.3	22.8	24.4
*Medium*	4,000	50.7	50.8	50.6	52.9	52.5	53.3	53.4	52.3	52.9	52.8
*Low*	1,941	24.6	25.2	24.3	23.5	24.1	22.9	22.8	24.4	24.3	22.7
Smoking in pregnancy (%)											
*Yes*	1,554	19.7	19.8	19.6	19.4	20.2	18.5	19.4	19.4	19.9	18.9
*No*	6,336	80.3	80.2	80.4	80.6	79.8	81.5	80.6	80.6	80.1	81.1
Physical activity in pregnancy^d^ (%)											
*Inactive*	4,876	61.8	61.1	62.1	61.4	62.0	60.7	60.6	62.3	60.4	62.3
*Medium Active*	781	9.9	9.8	10.0	10.1	9.6	10.7	10.9	9.2	10.6	9.7
*Highly Active*	2,233	28.3	29.1	27.8	28.5	28.5	28.6	28.5	28.5	29.0	28.0
Maternal pre-pregnancy BMI^e^ (%)											
*Underweight*	292	3.7	4.0	3.5	3.2	2.9	3.5	2.7	3.8	2.8	3.6
*Normal weight*	5,578	70.7	70.1	71.0	71.5	71.6	71.5	71.8	71.2	70.1	72.9
*Overweight*	1,491	18.9	18.5	19.1	18.8	19.2	18.4	18.6	19.0	20.0	17.5
*Obese*	529	6.7	7.4	6.4	6.5	6.3	6.6	6.8	6.0	7.0	6.0
Participated in two											
previous DNBC											
follow-ups^f^ (%)											
*Yes*	4,631	58.7	58.7	58.7	59.6	59.7	59.4	60.4	58.5	60.3	58.9
*No*	3,259	41.3	41.3	41.3	40.4	40.3	40.6	39.6	41.5	39.7	41.1
Adolescent characteristics at 6 months follow-up											
n	5,853		2,039	3,814	2,608	1,332	1,276	1,434	1,174	1,277	1,331
Mini-HEI	0.01 (0.59)		0.00 (0.60)	0.01 (0.59)	0.03(0.59)	0.03 (0.58)	0.03 (0.59)	0.04 (0.58)	0.02 (0.59)	0.03 (0.58)	0.03 (0.59)
BMIz	−0.09 (0.88)		−0.08 (0.89)	−0.09 (0.88)	−0.nn(0.88)	−0.11 (0.89)	−0.11 (0.88)	−0.12 (0.89)	−0.11 (0.88)	−0.10 (0.92)	−0.12 (0.85)
Adolescent characteristics at 18 months follow-up											
n	5,370		1,833	3,537	2,461	1,270	1,191	1,346	1,115	1,229	1,232
Mini-HEI	−0.01 (0.59)		0.00 (0.58)	−0.02 (0.59)	0.00 (0.59)	0.01 (0.59)	−0.01 (0.58)	0.00 (0.59)	0.00 (0.59)	0.01 (0.59)	0.00 (0.59)
BMIz	−0.06 (0.89)		−0.05 (0.87)	−0.06 (0.90)	−0.07 (0.88)	−0.06 (0.89)	−0.07 (0.88)	−0.07 (0.89)	−0.06 (0.88)	−0.05 (0.92)	−0.08 (0.85)

^a^ Adolescent HEI: Healthy Eating Index.

^b^ Adolescent BMIz: body mass index z-score defined by the Centers for Disease Control and Prevention (CDC) standard [36,37].

^c^ Maternal HEI: Healthy Eating Index, high: above third quartile (24.3 points), medium: first—third quartile, low: below first quartile (16.2 points).

^d^ Maternal physical activity level during pregnancy, measured in metabolic equivalents (METs): Inactive: METs score < 3, Medium active: METs score [3,6), Highly active: METs score ≥ 6.

^e^ Maternal pre-pregnancy BMI: body mass index: Underweight BMI <18.5 kg/m2, Normal weight BMI = 18.5–24.9 kg/m^2^, Overweight BMI = 25.0–29.9 kg/m^2^, Obese BMI>30 kg/m^2^.

^f^ DNBC follow-ups conducted at 6 months and 18 months postpartum.

^g^ Adolescents: Adolescents who at age 14 had completed a questionnaire assessing height, weight, and dietary habits.

DNBC, Danish National Birth Cohort; SMS, Short Messages Service, SD, standard deviation.

In Tables 3–5, we report results from both the ITT analyses and the inverse probability weighted (IPW-PP) analyses. All analyses were done in accordance with the pre-written SAP, available as S2 Text. None of the estimates from the predefined ITT analyses reached conventional statistical significance of *p* = 0.05.

Below, we highlight the inverse probability weighted effect estimates from the PP analyses as these express the effect of the intervention after accounting for non-participation in the offered SMS-program.

**Fig 3:**
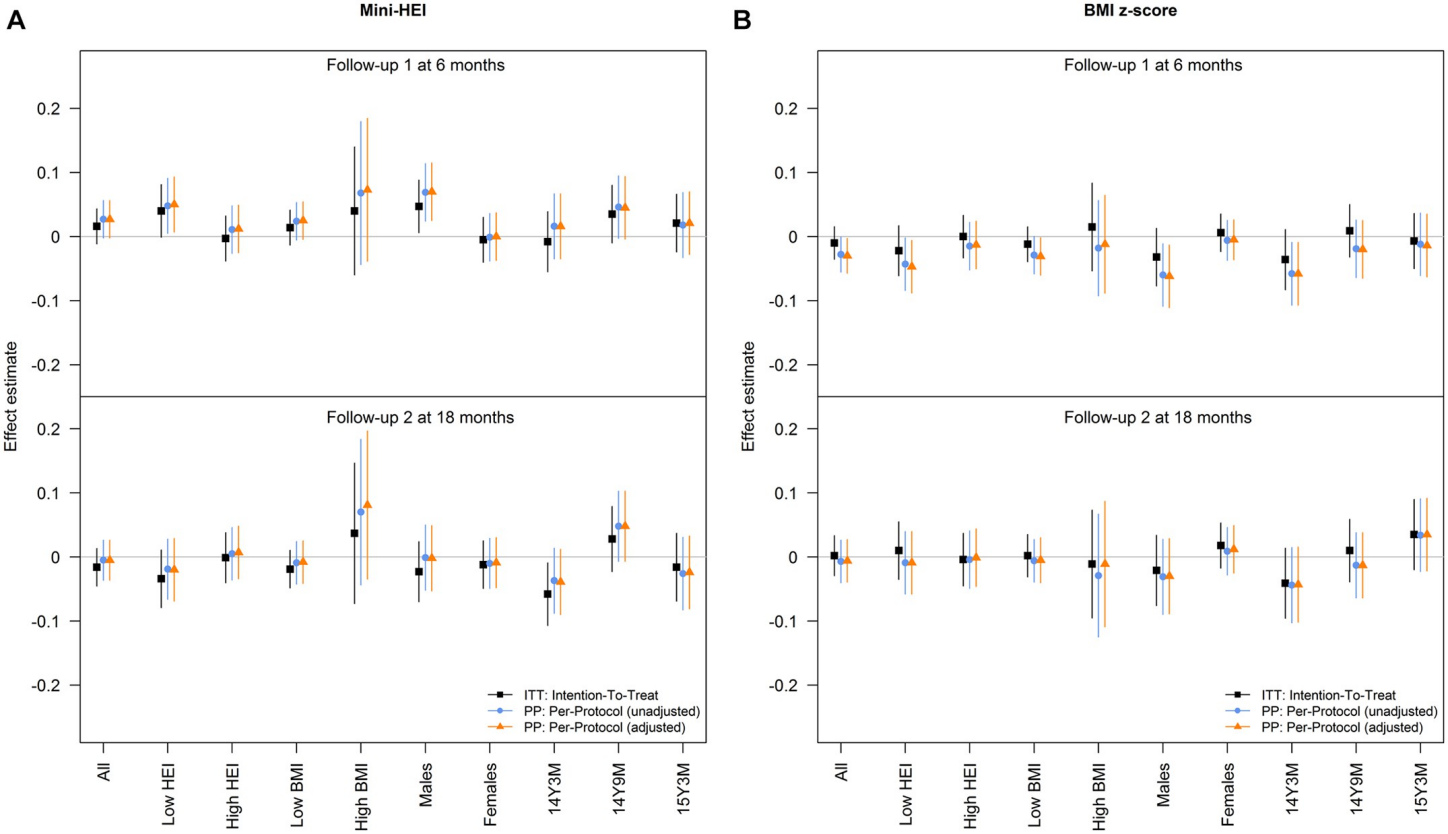
Effects of the SMS program on the mini-HEI and on the BMI z-scores. Estimated effects with 95% CIs of the SMS program on the abbreviated HEI and BMI z-scores among all adolescents and in subgroups consisting of those with below median HEI at baseline, those with above median HEI at baseline, those with low (below the 85th percentile) BMI z-score at baseline, those with high (above the 85th percentile) BMI z-score at baseline, males, females, and the 3 age groups (14 years and 3 months(m) (14Y3M), 14 years and 9 m (14Y9M), and 15 years and 3 m (15Y3M) at baseline). Effect estimates are estimated differences in means of outcomes at 6 m and 18 m follow-up among those allocated to the SMS program compared to those allocated to the control group receiving no SMS-program (non-SMS group). Colors indicate effects estimated from ITT-analyses including all individuals (black) and 2 PP-analyses excluding those who did not join the SMS program that they were offered; one accounting for a set of factors (maternal pregnancy healthy eating index score (low, medium, high), smoking in pregnancy (yes, no), physical activity level in pregnancy (low, medium, high metabolic equivalents (METS) score), pre-pregnancy BMI (underweight, normal weight, overweight, obese), and participation in the following previous DNBC follow-up surveys: when the child was 6 m, 18 m, and 7 years, respectively (“yes” to all vs. at least 1 “no”)) that may influence participation (orange) and one not accounting for these factors (blue). BMI, body mass index; CI, confidence interval; DNBC, Danish National Birth Cohort; HEI, Healthy Eating Index; ITT, intention-to-treat; PP, per-protocol; SMS, Short Messages Service.

Mean (standard deviation (SD)) HEI at baseline, 6 m and 18 m was −0.01 (0.64), 0.01 (0.59), and −0.01 (0.59), respectively (Table 2 and Fig 3). At 6 m, the estimated increase in mini-HEI in those who had received any SMS program compared to the non-SMS group (aggregated arms A1–A8 versus arm A9) was (inverse probability weighted estimate of effect) 0.027 [95% CI −0.002, 0.056]; *p* = 0.072. At 18 m, it was −0.005 [95% CI −0.036, 0.026]; *p* = 0.76. The joint test of effects at the 2 follow-up assessments was also nonsignificant (*p* = 0.12) (Table 3). CACE analysis yielded similar results, please see an alternative version of Table 3 using CACE, S1 Table.

**Table 3:** Effect estimates of primary outcomes endpoints among adolescents at 6- and 18-months follow-up receiving an SMS intervention compared with non-SMS group.

			ITT analyses^a^					IPW participant analyses^b^				
Effects on:	Effects of:	Follow-up	Estimate	95% CI		*p* ^c^	*p* ^d^	Estimate	95% CI		*p* ^c^	*p* ^d^
**Mini HEI**	**AnySMS vs. Non-SMS**	6 months	0.016	−0.011	0.043	0.253	0.172	0.027	−0.002	0.056	0.072	0.117
		18 months	−0.016	−0.045	0.013	0.286		−0.005	−0.036	0.026	0.755	
**BMIz-score**	**AnySMS vs. Non-SMS**	6 months	−0.010	−0.035	0.015	0.442	0.635	−0.030	−0.057	−0.003	0.032	0.092
		18 months	0.002	−0.029	0.033	0.901		−0.006	−0.039	0.027	0.724	

All analyses are adjusted for the full combination of sex, age group, and diet strata and the baseline value corresponding to the outcome.

^a^ ITT: intention-to-treat analysis comparing all adolescents randomized to (any) SMS intervention with non-SMS group.

^b^ IPW: inverse probability weighting analysis comparing all adolescents randomized to (any) SMS intervention with non-SMS group. Analyses excluding non-participants of the SMS intervention. Observations are weighted by their inverse probability of participation given their predictors at DNBC baseline and analyses are additionally adjusted for grouped (10 groups) propensity scores.

^c^ *p*-Value for individual test testing effects at each follow-up time

^d^ *p*-Value for joint test testing effects at 6 months and 18 months at the same time.

DNBC, Danish National Birth Cohort; SMS, Short Messages Service; SSB, sugar-sweetened beverages; FV, fruits and vegetables; HEI, Healthy Eating Index constitutes of SSB, FV, and fish; BMI, body mass index z-score defined by the Centers for Disease Control and Prevention (CDC) standard [36,37].

At 6 m, a statistically significant decrease in mean BMI z-score was observed in those who had received any SMS program compared to the non-SMS group (−0.030 [95% CI −0.057, −0.003]; *p* = 0.032) (Table 3 and Fig 3). At 18 m, this effect had faded (−0.006 [95% CI −0.039, 0.027]; *p* = 0.72), and the joint test at the 2 follow-ups was not significant (*p* = 0.09). Both at 6 m and 18 m, the risk of overweight was similar among those who had received any SMS program compared to those in the non-SMS group (RR 1.04 [95% CI 0.90, 1.20] and 1.07 [95% CI 0.91, 1.26]; respectively; joint test *p* = 0.71) (Table 4).

**Table 4:** The risk of being above the 85th and 95th percentiles of the population BMIz-score at 6- and 18-months follow-up among adolescents participating in the SMS intervention compared with non-SMS group.

		ITT analyses^a^			IPW participant-analyses^b^		
	Follow-up	RR (95% CI)	*p* ^c^	*p* ^d^	RR (95% CI)	*p* ^c^	*p* ^d^
**BMIz85**	6 months	1.09 (0.95, 1.24)	0.210	0.410	1.04 (0.90, 1.20)	0.603	0.711
	18 months	1.07 (0.92, 1.24)	0.391		1.07 (0.91, 1.26)	0.432	
**BMIz95**	6 months	0.90 (0.65, 1.23)	0.494	0.537	0.78 (0.54, 1.12)	0.176	0.216
	18 months	1.10 (0.75, 1.62)	0.610		1.09 (0.72, 1.65)	0.686	

All analyses are adjusted for sex, age group, diet strata, and the baseline value corresponding to the outcome.

^a^ ITT: intention-to-treat analysis comparing all adolescents randomized to (any) SMS intervention with non-SMS group.

^b^ IPW: inverse probability weighting analysis. Analyses excluding non-participants of the SMS intervention. Observations are weighted by their inverse probability of participation given their predictors at DNBC baseline and analyses are additionally adjusted for grouped (10 groups) propensity scores.

^c^ *p*-Value for individual test testing effects at each follow-up time.

^d^ *p*-Value for joint test testing effects at 6 months and 18 months at the same time.

DNBC, Danish National Birth Cohort, SMS, Short Messages Service; RR, risk ratio; CI, confidence interval; BMI, body mass index z-score defined by the Centers for Disease Control and Prevention (CDC) standard [36,37].

### Effects on HEI and BMI z-score in adolescents with low diet quality at baseline

In analyses confined to adolescents with an HEI below the median of the standard normal distribution at baseline (*n* = 3,748), the estimate of the association at 6 m was statistically significant, 0.050 [95% CI 0.007, 0.093]; *p* = 0.023, whereas the estimate at 18 m (−0.020 [95% CI −0.069, 0.029]; *p* = 0.424) was, again, compatible with no effect at this point in time (Fig 3). In these adolescents (*n* = 3,748), the association with mean BMI z-score at 6 m was more pronounced (−0.047 [95% CI −0.088, −0.006]; *p* = 0.025) than in the unselected sample (−0.030 SD ([95% CI −0.057, −0.003]; *p* = 0.032)). Again, this association had disappeared at 18 m (−0.009 [95% CI −0.058, 0.040]; *p* = 0.719).

### Effects on BMI z-score in adolescents who were overweight at baseline

In analyses restricted to adolescents with a BMI z-score above the 85th percentile of the standard normal distribution at baseline (*n* = 671), we observed no association with mean BMI z-score at 6 m (−0.012 [95% CI −0.088, 0.064]; *p* = 0.77) or 18 m (−0.011 [95% CI −0.109, 0.087]; *p* = 0.88) follow-up.

### Effects according to sex

The above reported increasing effect at 6 m on mini-HEI of any SMS program compared to the non-SMS group was only apparent in males, with no effects apparent in females (males, *n* = 3,644: 0.070 [95% CI 0.025, 0.115]; *p* = 0.002, and females, *n* = 4,246: 0.000 [95% CI −0.037, 0.037]; *p* = 1.0) (test for interaction by sex: *p* = 0.025). The same was true for the lowering effect on BMI at 6 m of any SMS program compared to the non-SMS group (males: −0.062 [95% CI −0.111, −0.013]; *p* = 0.013, and females: −0.005 [95% CI −0.036, 0.026]; *p* = 0.78) (test for interaction by sex: *p* = 0.047). These effect estimates are visualized in Fig 3.

When looking at males and females with low dietary quality at baseline, respectively, at 6 m, the difference in BMI was estimated to −0.086 ([95% CI −0.155, −0.017]; *p* = 0.014) among males and −0.003 ([95% CI −0.052, 0.046]; *p* = 0.90) in females (interaction by sex: *p* = 0.1067) for those with low diet quality at baseline, whereas the corresponding differences in HEI were estimated to 0.107 ([95% CI 0.044, 0.170]; *p* = 0.001) and −0.003 ([95% CI −0.064, 0.058]; *p* = 0.92) for the 2 sexes (interaction by sex: *p* = 0.0371), respectively. At 18 m, all associations observed at 6 m in these subgroup analyses had disappeared.

### Effects according to age

Overall, no clear differences in the effect estimates were observed between the 3 age groups (Fig 3).

### Effects on the 3 individual target diets underlying the HEI calculation: SSB, FV, and fish intake

**Fig 4:**
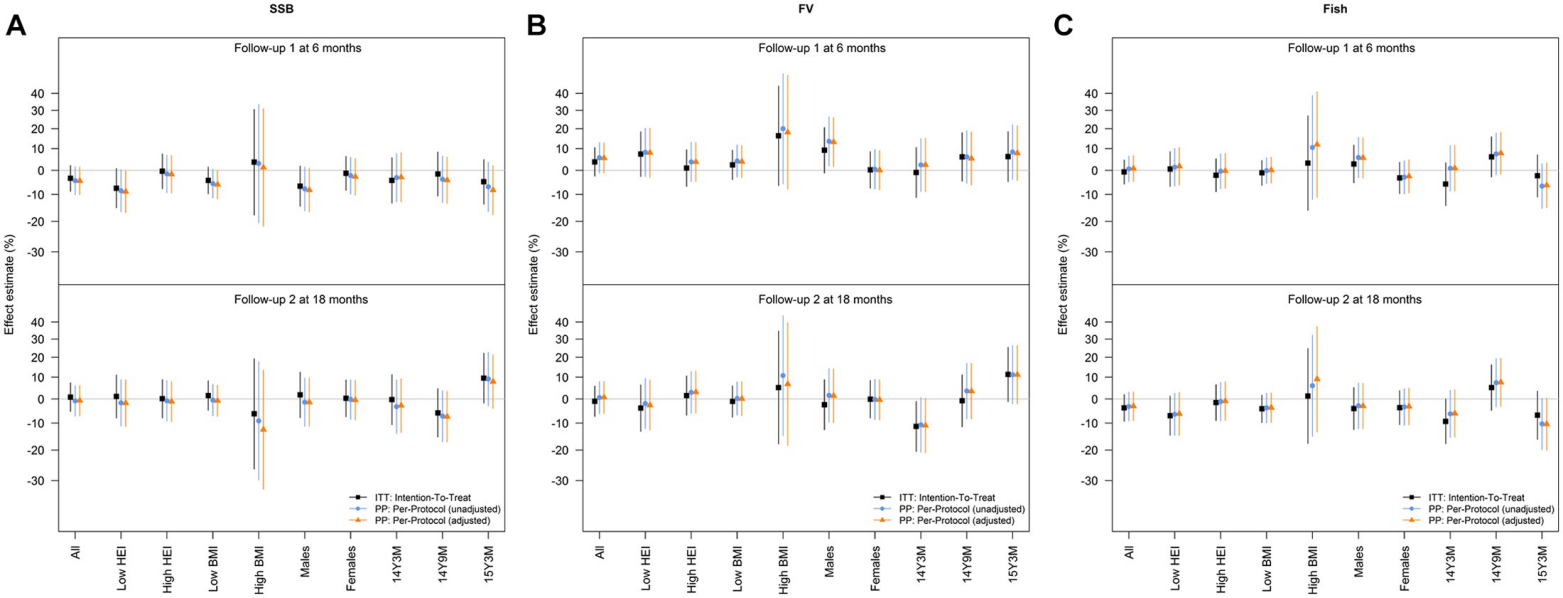
Effects of the SMS program on intake of SSBs, intake of FV, and intake of fish. Estimated effects with 95% CIs of the SMS program on SSBs, FV, and fish among all adolescents and in subgroups consisting of those with below median HEI at baseline, those with above median HEI at baseline, those with low (below the 85th percentile) BMI z-score at baseline, those with high (above the 85th percentile) BMI z-score at baseline, males, females, and the 3 age groups (14 years and 3 months (m) (14Y3M), 14 years and 9 m (14Y9M), and 15 years and 3 m (15Y3M) at baseline). Effect estimates are estimated relative differences in intake of the outcomes at 6 m and 18 m follow-up among those allocated to the SMS program compared to those allocated to the control group receiving no SMS program (non-SMS group). Colors indicate effects estimated from ITT analyses including all individuals (black) and 2 PP analyses excluding those who did not join the SMS program that they were offered; one accounting for a set of factors (maternal pregnancy healthy eating index score (low, medium, high), smoking in pregnancy (yes, no), physical activity level in pregnancy (low, medium, high metabolic equivalents (METS) score), pre-pregnancy BMI (underweight, normal weight, overweight, obese), and participation in the following previous DNBC follow-up surveys: when the child was 6 m, 18 m, and 7 years, respectively (“yes” to all vs. at least 1 “no”)) that may influence participation (orange) and one not accounting for these factors (blue). BMI, body mass index; CI, confidence interval; DNBC, Danish National Birth Cohort; FV, fruit and vegetables; HEI, Healthy Eating Index; ITT, intention-to-treat; PP, per-protocol; SMS, Short Messages Service; SSB, sugar-sweetened beverage.

Associations with each of these secondary outcomes are presented in Table 5 and Fig 4. No associations were detected in the ITT analyses on the separate measures for SSB, FV, and fish intake. However, according to the inverse probability weighted PP analyses, participation in any SMS program compared to the non-SMS group was associated with an increase in FV of 13.2% ([95% CI 1.6%, 26.1%], *p* = 0.024) at 6 m in male participants; no such association was observed in female participants (estimate: 0.1% [95% CI −8.2%, 9.1%], *p* = 0.982) (interaction by sex: *p* = 0.2515) (see Fig 3). When analyses were limited to only include adolescents with low diet quality at baseline, participating in any SMS program was associated with a higher intake of FV by 25.1% [95% CI 7.0%, 46.3%], *p* = 0.005 in males and −5.5% [95% CI −18.6%, 9.6%], *p* = 0.61 for females (interaction by sex: *p* = 0.0425). For SSB intake effects, the corresponding measures of association for SSB intake were −8.3% [95% CI −16.6%, 1.0%]; *p* = 0.079 (“minus” here denoting a reduction) in the males and −2.8% [95% CI −10.3%, 5.4%], *p* = 0.50 in the females (interaction by sex: *p* = 0.40 in the unselected sample, and, in adolescents with low diet quality at baseline, −11.0% [95% CI −21.8%, 1.2%], *p* = 0.08 in the males and −6.9% [95% CI −18.5%, 6.4%], *p* = 0.29 in the females (interaction by sex: *p* = 0.65). The measures of association for fish intake were generally null.

**Table 5:** Effect estimates on intake of SSB, FV, and fish.

				ITT analyses^a^					IPW participant analyses^b^			
Effects on:	Effects of:	Follow-up	Estimate	StErr	*p* ^c^	*p* ^d^	Effect %	Estimate	StErr	*p* ^c^	*p* ^d^	Effect %
**SSB**	**AnySMS vs. Non-SMS**	6 months	−0.035	0.029	0.227	0.370	−3.4	−0.046	0.031	0.138	0.323	−4.5
		18 months	0.008	0.032	0.803		0.8	−0.007	0.034	0.837		−0.7
**FV**	**AnySMS vs. Non-SMS**	6 months	0.037	0.032	0.248	0.404	3.8	0.054	0.034	0.112	0.283	5.5
		18 months	−0.010	0.034	0.769		−1.0	0.007	0.036	0.846		0.7
**Fish**	**AnySMS vs. Non-SMS**	6 months	−0.007	0.027	0.795	0.423	−0.7	0.008	0.029	0.783	0.527	0.8
		18 months	−0.039	0.030	0.194		−3.8	−0.032	0.032	0.317		−3.1

All analyses are adjusted for the full combination of sex, age group, and diet strata and the baseline value corresponding to the outcome.

^a^ ITT: intention-to-treat analysis comparing adolescents randomized to SMS intervention with one of the 3 additional elements; adding a friend, adding the mother, or tailored text messages to adolescents without the element.

^b^ IPW: inverse probability weighting analysis. Analyses excluding non-participants of the SMS intervention. Observations are weighted by their inverse probability of participation given their predictors at DNBC baseline and analyses are additionally adjusted for grouped (10 groups) propensity scores.

^c^ *p*-Value for individual test testing effects at each follow-up time

^d^ *p*-Value for joint test testing effects at 6 months and 18 months at the same time.

DNBC, Danish National Birth Cohort; SMS, Short Messages Service; StErr, standard error; Effect %, percentage increase or decrease in diet intake of specific intervention group compared to connected control group; SSB, sugar-sweetened beverages; FV, fruits and vegetables.

### Analyses limited to the factorial component (arms A1–A8) excluding arm A9

In Table 6, results from testing hypotheses 2 to 4 are reported, both by ITT analyses and by (IPW-PP) analyses; all analyses were done in accordance with the pre-written SAP. Effect estimates for examining these hypotheses in subgroups can furthermore be seen in S3 Fig.

**Table 6:** Effect estimates of adding friend, adding mother, or tailored to the SMS intervention among adolescents at 6- and 18-months follow-up.

			ITT analyses^a^				IP-weighted participant analyses^b^			
		Follow-up	Estimate	95% CI	*p* ^c^	*p* ^d^	Estimate	95% CI	*p* ^c^	*p* ^d^
**Mini HEI**	**Mother**	6 months	−0.004	−0.035, 0.027	0.803 0.953	0.96	0.002	−0.035, 0.039	0.916 0.568	0.80
		18 months	0.001	−0.032, 0.034			−0.012	−0.053, 0.029		
	**Friend**	6 months	−0.029	−0.060, 0.002	0.070 0.638	0.20	−0.042	−0.081, −0.003	0.036 0.600	0.09
		18 months	−0.008	−0.041, 0.025			−0.011	−0.052, 0.030		
	**Tailor**	6 months	−0.009	−0.040, 0.022	0.574 0.597	0.81	−0.008	−0.047, 0.031	0.689 0.849	0.92
		18 months	−0.009	−0.042, 0.024			−0.004	−0.045, 0.037		
**BMIz-score**	**Mother**	6 months	0.006	−0.023, 0.035	0.689 1.000	0.91	0.009	−0.028, 0.046	0.636 0.820	0.80
		18 months	0.000	−0.037, 0.037			−0.005	−0.048, 0.038		
	**Friend**	6 months	0.014	−0.015, 0.043	0.351 0.046	0.12	0.016	−0.021, 0.053	0.400 0.256	0.48
		18 months	0.038	0.001, 0.075			0.025	−0.018, 0.068		
	**Tailor**	6 months	−0.008	−0.037, 0.021	0.594 0.528	0.79	0.011	−0.026, 0.048	0.563 0.928	0.84
		18 months	−0.012	−0.049, 0.025			0.002	−0.041, 0.045		
**SSB**	**Mother**	6 months	−0.019	−0.086, 0.048	0.576 0.978	0.83	−0.033	−0.113, 0.047	0.421 0.579	0.48
		18 months	0.001	−0.072, 0.074			0.025	−0.063, 0.113		
	**Friend**	6 months	0.005	−0.062, 0.072	0.883 0.344	0.63	0.011	−0.069, 0.091	0.788 0.768	0.94
		18 months	0.035	−0.038, 0.108			0.013	−0.073, 0.099		
	**Tailor**	6 months	0.008	−0.059, 0.075	0.814 0.048	0.13	0.013	−0.069, 0.095	0.757 0.091	0.23
		18 months	0.073	0.000, 0.146			0.076	−0.012, 0.164		
**FV**	**Mother**	6 months	−0.089	−0.163, −0.015	0.019 0.739	0.06	−0.093	−0.185, −0.001	0.048 0.419	0.14
		18 months	−0.013	−0.089, 0.063			−0.038	−0.130, 0.054		
	**Friend**	6 months	−0.056	−0.130, 0.018	0.141 0.918	0.32	−0.050	−0.140, 0.040	0.277 0.655	0.39
		18 months	−0.004	−0.080, 0.072			0.021	−0.071, 0.113		
	**Tailor**	6 months	−0.038	−0.112, 0.036	0.317 0.293	0.23	−0.027	−0.119, 0.065	0.566 0.142	0.18
		18 months	0.041	−0.035, 0.117			0.069	−0.023, 0.161		
**FISH**	**Mother**	6 months	0.047	−0.014, 0.108	0.129 0.576	0.32	0.055	−0.018, 0.128	0.137 0.733	0.33
		18 months	0.019	−0.048, 0.086			0.014	−0.066, 0.094		
	**Friend**	6 months	−0.048	−0.109, 0.013	0.122 0.837	0.24	−0.095	−0.168, −0.022	0.010 0.283	0.03
		18 months	0.007	−0.060, 0.074			−0.044	−0.124, 0.036		
	**Tailor**	6 months	0.006	−0.055, 0.067	0.847 0.930	0.98	0.005	−0.068, 0.078	0.893 0.903	0.98
		18 months	0.003	−0.064, 0.070			−0.005	−0.085, 0.075		

All analyses are adjusted for the full combination of sex, age group, and diet strata and the baseline value corresponding to the outcome.

^a^ ITT: intention-to-treat analysis comparing adolescents randomized to SMS intervention with one of the 3 additional elements; adding a friend, adding the mother, or tailored text messages to adolescents without the element.

^b^ IP: inverse probability weighting analysis. Analyses restricted to participants of the interventions including participation of one of the 3 additional elements; a friend, the mother, or tailored text messages. Observations are weighted by their inverse probability of participation given their predictors at DNBC baseline and analyses are additionally adjusted for grouped (10 groups) propensity scores.

^c^ *p*-Value for testing effects at 6 months and 18 months, separately.

^d^ *p*-Value for joint test testing effects at 6 months and 18 months at the same time.

Effect %: percentage increase or decrease in diet intake of specific intervention group compared to connected control group.

HEI, Healthy Eating Index constitutes of SSB, FV, fish; BMI, body mass index z-score defined by the Centers for Disease Control and Prevention (CDC) standard [36,37]; SSB, sugar-sweetened beverages; FV, fruits and vegetables.

Among adolescents randomized to invite their mother, 49.0% (1,443/2,630) had their mother join, whereas among adolescents randomized to invite 1 friend, 20.0% (517/2,630) had a friend join.

No clear effects, neither in ITT analyses or IPW-PP analyses, were observed of inviting the mother on neither HEI nor BMI z-score.

Neither were any beneficial effects on HEI nor BMI observed for inviting a friend.

No clear differences, neither in HEI nor in BMI z-scores, were observed between offering an SMS education program that targeted a specific dietary factor, tailored to match the adolescents’ individual risk profiles, as compared to offering an SMS education program generically designed to target a standard mixed education of various dietary factor.

## Discussion

In this large trial, we examined effects of participating in an SMS-based health educational program on adolescent dietary behaviors and body weight trajectories. None of the ITT analyses exhibited significant beneficial effects. It is important to note, however, that 37% chose not to participate in the educational program they had been allocated to. In light of this non-participation, we therefore undertook the IPW-PP analyses, which revealed interesting patterns. Notably, in these analyses among adolescents who had joined the offered educational program, we observed, at 6 m, that participating in any variant of the SMS-based health educational programs was associated with a modestly lower BMI z-score and higher diet quality, as compared to those who had not been offered to participate in any SMS program.

We noted different patterns among male and female adolescents. As per FDA-recommended best practices, we examined the effects separately in male and female adolescents and tested for effect modification. These analyses suggested modest weight trajectory and dietary behavior improvements at 6 months in the males only. However, neither male nor female subjects showed any improvements at 18 months.

RCTs of effect of digital inverventions to induce healthier dietary habits among adolescents have generally been much smaller than our individual-based RCT, or used cluster randomization with schools [39,40] or school classes [41] as randomization units enabling inclusion of large numbers of adolescents. Two cluster RCTs in Denmark, one among >4,000 adolescents examining a multicomponent intervention including a smartphone application [39], the other among 1,488 adolecents employing SMS [41] like in our trial, reported no effects on dietary habits or BMI. A cluster RCT in Brazil among 895 adolescents [40], examining the effect of participating in an online program encouraging and guiding weight control and healthy eating habits, reported increased intake of beans and reduced intake of soft drinks but no effects on anthropometric parameters. The program included the participation of parents and teachers, but effects of these elements of the intervention were potentially confounded by effects of other components, such as information the adolescents had retrieved on their own by accessing the online program. In a 2020 review [14] Partridge and colleagues noted that high-quality studies that evaluate text messages as the only modality of intervention delivery are required. We are not aware of other studies that have attempted to examine simultenously the separate effects of activiting members of adolescents’ social network and the separate effects of text messages sent to the adolescents, as we could by virtue of our factorial design.

In fact, by creating 4 distinct randomizations we were able to effeciently operate what is effectively 4 trials in one. Uniquely, this enabled us to take a higher resolution look into how subtle variations of the same SMS program might be associated with improvements or degradation or no change of dietary behaviors and weight trajectories. The absence of significant results for activation of peer and familial forces, may suggest that in adolescent settings, dietary and lifestyle coaching may be better served in a private setting. This is in contrast with some studies in adult populations that have suggested benefit from social support [42].

Besides the factorial design, our study had many strengths, but also limitations. It was a large, randomized controlled trial. Generalizability to other populations beyond DNBC remains to be studied. However, we mitigated this limitation by undertaking analyses in disadvantaged subsections in the DNBC database. Because we used the DNBC as recruitment frame we had, as a unique feature of the study, access to detailed baseline information on this large group of 14-year-old adolescents. Although mothers who initially populated the DNBC though derived from the general population, tended to be healthier and better educated than the average population [43], we were able to examine effects in adolescents with a presumed greater room for improvement. Given that the observed effects of the program seemed more pronounced among those 50% of adolescents who had the poorest dietary quality, we would expect that the program would offer the greatest benefit among this sub-segment of the general population most in need of it. Correspondingly, among adolescents with a BMI above the 85th or 95th BMI percentiles at baseline, we examined the effects of our program on risk of overweight and obesity and on BMI z-score, but were unable to show significant beneficial effects on these outcome measures in these subsections of the trial. This latter is in contrast to a meta-analysis, including 18 RCTs, which concluded that E-health interventions can be effective in treating children and adolescents with overweight and obesity [43]; numbers and thus statistical power were possibly too low in these subsections in our trial.

Other limitations of the program include the imperfect follow-up. In a population of adolescents, however, we managed to obtain a follow-up rate on outcomes of around 70% within 6 m and 18 m, which may be considered good bearing in mind that we had no direct contact with the adolescents. Though the participants were not prompted to invite a friend and there was no advice or guidelines as to who they could invite, we learned via interviews that some cohort members were put off by the idea of having to invite a friend, so much so that they chose not to participate at all; social desirability bias may have been at play here because, among adolescents randomized to invite a friend, only 20.0% (517/2,630) had a friend join. Even though it was optional to invite a friend, the project felt too private for a friend to be involved, and we believe this sentiment reflects the feeling cohort participants often have when they have been part of a cohort study since birth. Furthermore, dietary behaviors were self-reported, along with height and weight, and the adolescents were not asked to measure themselves before completing the questions; notably, the assessment method used to assess dietary intake at baseline was validated [24,25], but the assessments of height and weight were not. Desirability bias, or social approval bias is a common problem when assessing dietary habits by self-report [44]. Although everything was anonymous (meaning that it is not like anyone would be embarrassed, or held accountable), we cannot exclude that such mechanisms may have played a role for how those adolescents who had participated in the offered SMS-based educational program responded to the dietary questions at the 6 m follow up; but desirability bias can hardly explain the lower BMI z-scores observed in that group. Importantly, because we randomized prior to offering the adolescents the specific program, the control group were unaffected by information about the program, thus avoiding possible co-intervention, or “contamination”, bias. For example, only the SMS-receiving groups were informed about the social media/Instagram components but the non-SMS group (arm 9) might have stumbled across the project Instagram account (SundmedSMS), as access was not limited in any way. Taking the rather limited SoMe activity among the SMS participants into consideration we do not believe the non-SMS group had any noteworthy interaction with this element of the study.

An important limitation was that 37% chose to not participate in the offered SMS educational program. Therefore, randomization does not ensure that participants are comparable to the reference group at baseline. In per protocol analyses excluding non-participants, we attempted to address this issue by using inverse probability weighting (the IPW-PP analyses). The ITT results are not affected by this limitation but targets the slightly different question of estimating the average effect of offering the program which expectedly is numerically smaller than the average effect of the program among participants only targeted by the IPW-PP analyses.

In the IPW-PP analyses, we observed a noteworthy consistency between the 2 outcomes, BMI and diet quality. Particularly, the observation that no association for any could be seen at 18 m but for both at 6 m is interesting as it may confer credibility that the associations we see at 6 m reflect causality. Thus, we might reasonably expect that effects of an intervention lasting only 12 weeks (for the shorter program: only 4 weeks) would have vanished 15 m (for the shorter program: 17 m) after the end of the active intervention. Assuming this to be the case, it would mean that the observed estimates of association at 18 m, indicating no effect in the IPW-PP analyses on either outcome could actually be a correct result; or, in other words, be unbiased, despite the deviation from the randomization scheme resulting from the 37% non-participation; and by analogy that the associations actually observed for both the HEI and BMI z-score at the 6 m assessment—because the analytic conditions with respect to the 37% non-participation were identical at the 2 points in time—might also be unbiased and thus reflect true causal effects of the SMS program on the 2 outcomes. Other consistencies between the 2 outcomes were also observed: in subgroups defined by low diet quality at baseline, and in males versus females.

The study has broad public health and clinical relevance, given that poor nutrition accounts for substantial proportions of occurrences of ill-health and mortality in modern societies. The associations observed between participating in an SMS-based educational program and a subsequent higher diet quality and lower BMI z-score were clearest among those in most need of improvement in diet quality. Similar findings have been seen in other interventions for weight control among adolescents, for example, in a trial displacing SSB with noncaloric beverages during 25 weeks [45]. Our observation, in male adolescents with low diet quality at baseline, of an effect of participating in any SMS-based educational program on mean BMI z-score, is interesting in the light of the earlier mentioned 2019 Cochrane review, testing the effectiveness of a range of different interventions, and which found limited effective interventions on BMI z-scores for adolescents aged 13 to 18 years old [7]. Our trial suggests that policy makers might be able to use and further optimize low- or no-cost SMS interventions to improve dietary behaviors and metabolic health in adolescents.

It can be difficult to interpret effect sizes and standard errors on the BMI z-score scale. Null effects of the BMI z-score scale transfer to null effects when measured in kilos. The standard errors of the estimated effects of receiving any SMS program as compared to the non-SMS group are close to 0.015. For adolescents with about average height and weight this roughly corresponds to a standard error of about 0.15 kg for the estimated (null) effect in kilos. While each 1 kg of weight loss is estimated to decrease risk of progression to type 2 diabetes by 16% [46], such protective effects of weight loss seem not to have been documented in adolescents. While the association measures of the intervention were relatively small at 6 m from onset of the intervention and faded after 18 m, it is possible that the passage of time diminished adherence. The benefits of a more sustained program need to be evaluated. Future trials should investigate the efficacy of “booster” chatbot interventions in sustaining dietary and weight trajectory improvements.

Whereas the measures of association seen for HEI do not either translate directly to measures of physical quantities, the measures of association for the 3 individual target diets underlying the HEI calculation, SSB, FV, and fish intake, do. It is therefore interesting to note that according to the IPW-PP analyses, participating in any SMS program was associated with a higher intake of FV by 13% in males, and that when analyses were limited to only include adolescents with low diet quality at baseline, participating in any SMS program was associated with a higher intake of FV by 25% in males, with no significant associations in females. These findings are interesting seen in the light of a recent systematic review concluding that few digital interventions have been effective in increasing vegetable intake among adults [47]; it could be speculated that a text messaging intervention is more appealing to adolescents, especially adolescent males, and may increase self-efficacy to a larger extent in this group. Thus, in a study among Danish adolescents, participants with higher level of engagement in a text messaging fruit and vegetable school intervention increased intake whereas those with low engagement, did not [41]; the authors suggested this to be mediated through increased self-efficacy. For SSB intake, again according to the IPW-PP analyses, associations were significant for the subgroup of males with low diet quality at baseline and indicated a decrease in SSB intake of 11%. Few studies have stratified SSB intake by sex and in general SSB intake is higher in boys than in girls [48,49] also in treatment seeking obese adolescents [50]. Since 2% to 4% of total energy intake comes from SSB in children and adolescents in Denmark, the reductions observed in our study would be relevant to improve the nutritional status [51]. The measures of association for fish intake were generally null.

Recent studies have highlighted the importance of analyzing sex weight loss patterns separately [52–55], and sex differences are important factors in interpreting program efficacy. Due to societal norms, it has been observed that males tend to desire weight gain whereas females desire weight loss [52]. Weight gaining or “bulking” behaviors among male adolescents and eating disorders among females have been documented elsewhere as areas of concern [56,57]. The US CDC documented a higher rate of weight loss seeking behaviors among female (45.2%) versus male (30.1%) adolescents [58]. A study undertaken in the Avon Longitudinal Study of Parents and Children cohort, which, although older, is likely to have many similarities to our DNBC cohort, also found dieting to be much more prevalent in females than males at age 14 [59]. It is therefore noteworthy that our program suggested weight loss efficacy among the subpopulation, male adolescents, most likely to seek weight gain. Further studies should investigate the psycho-social dimensions of this phenomenon and any possible protective effects of a digital SMS intervention against harmful dietary behaviors, eating disorders, and bodily dysphoria. Irrespective of the mechanisms that may be underlying the patterns we observe, our results emphasize that prevention strategies in this field need to be sex-specific.

Our study documented high feasibility and relatively high acceptability in young adolescents of a digital educational program lasting up to 12 weeks. Because the program was entirely based on automatized text messaging via smartphones, and in certain randomized modalities tailored to individual baseline risk factors, interventions based on this technology are scalable. This could potentially be offered to adolescents in clinical practices that for various reasons are (e.g., because they are too stressed) unwilling or unable to engage in 1:1 counselling. Our findings suggest that sustained engagement will be necessary. Other elements that could improve acceptability and engagement include program launching strategies via schools and free media publicity, and prizes and raffles, and competitions could be tied to both engagement and quiz scores [41]. We concur with Partridge and colleagues [14,60,61] that future research should focus on separating the effects of only text message interventions from other modalities, such as we were able to do with our factorial design, and that co-designing, personalization, and Just-in-Time-Adaptation are important principles to take into account when developing the programs.

In conclusion, this study could not document an effect of offering adolescents our SMS-based educational programs on BMI or dietary quality. However, analyses among those who participated in the offered programs were suggestive of beneficial effects lasting up to 6 m after program commencement, particularly so among the males, and among those with a beforehand low dietary quality. Another outcome of our study is that it provides indications for how the power of technology could be harnessed to deliver low-cost, targeted messaging that can improve health at scale. Further studies that seek to optimize messaging effects, especially among female research subjects is needed, along with the development of “booster” messaging blasts to sustain long-term outcomes.

## Supporting information

S1 Text 1 Duration, structure and content of the SMS messaging programs. 2 Definition of pre-randomization strata (more detailed and concise descriptions can be provided). 3 Ethical and scientific considerations underlying the choice of the program’s three target factors: Lowering the intake of sugar sweetened beverages, increasing the intake of fruit and vegetables, and increasing the intake of fish.
(DOCX)

S2 Text Statistical Analysis Plan (SAP) for Texts For Healthy Teens: A Health Education Program for Adolescents (T4HT). (PDF)

S3 Text Protocol sent to The Scientific Ethical Committee. (PDF)

S1 Table Table 3 with an alternative statistical method for adjustment: CACE. (DOCX)

S1 CONSORT Checklist CONSORT 2010 checklist of information to include when reporting a randomised trial. (DOC)

S1 TIDieR Checklist The TIDieR (Template for Intervention Description and Replication) Checklist. (DOCX)

S1 Fig Time schedule for sending invitations in the three age strata. (PPTX)

S2 Fig Example of an SMS script from Day 3. (TIF)

S3 Fig Main results for effects on mini-HEI and BMI z-scores, and SSB, FV and Fish z-scores, for all adolescents (the whole trial population). Analyses of the two main outcomes, mini-HEI and BMI z-score, are shown in the upper and lower left panel, respectively, on each page; whereas analyses of the secondary outcomes, SSB, FV and Fish z-scores, are shown in the upper, middle and lower right panel, respectively. In each panel are shown analyses of the effect estimates for the continuous outcomes performed for AnySMS compared to Non-SMS (the estimate to the left in each panel); and for each of the three additional elements, i.e. adding mother compared to not adding mother, adding friend compared to not adding friend, or tailored SMS program compared to the full SMS program, assessed within the group of AnySMS, thus excluding the Non-SMS group (the three estimates to the right in each panel, respectively). Effect sizes are estimated differences between comparison groups in means of outcomes at 6 months and 18 months follow-up. Colors indicate effects estimated from intention-to-treat (ITT)-analyses including all individuals (black) and two Per Protocol-analyses excluding those who did not join the SMS-program that they were offered; one accounting for a set of factors (maternal pregnancy healthy eating index score (low, medium, high), smoking in pregnancy (yes, no), physical activity level in pregnancy (low, medium, high metabolic equivalents (METS) score), pre-pregnancy BMI (underweight, normal weight, overweight, obese), and participation in the following previous DNBC follow-up surveys: when the child was 6 m, 18 m and 7 years, respectively (‘yes’ to all vs. at least one ‘no’)) that may influence participation (orange) and one not accounting for these factors (blue). Values presented by a dot show data at 6 months follow-up whereas values presented by a triangle show data at 18 months follow-up.
IP: Inverse Probability, 95% CI: 95% Confidence Interval, SSB: Sugar sweetened beverages, FV: Fruits and vegetables, BMI: Body Mass Index, HEI: Healthy Eating Index, DNBC: Danish National Birth Cohort, y: Years, m: Months, SMS: Short Messages Service.
(TIF)

S4 Fig Main results for effects on mini-HEI and BMI z-scores, and SSB, FV and Fish z-scores, for males. Analyses of the two main outcomes, mini-HEI and BMI z-score, are shown in the upper and lower left panel, respectively, on each page; whereas analyses of the secondary outcomes, SSB, FV and Fish z-scores, are shown in the upper, middle and lower right panel, respectively. In each panel are shown analyses of the effect estimates for the continuous outcomes performed for AnySMS compared to Non-SMS (the estimate to the left in each panel); and for each of the three additional elements, i.e. adding mother compared to not adding mother, adding friend compared to not adding friend, or tailored SMS program compared to the full SMS program, assessed within the group of AnySMS, thus excluding the Non-SMS group (the three estimates to the right in each panel, respectively). Effect sizes are estimated differences between comparison groups in means of outcomes at 6 months and 18 months follow-up. Colors indicate effects estimated from intention-to-treat (ITT)-analyses including all individuals (black) and two Per Protocol-analyses excluding those who did not join the SMS-program that they were offered; one accounting for a set of factors (maternal pregnancy healthy eating index score (low, medium, high), smoking in pregnancy (yes, no), physical activity level in pregnancy (low, medium, high metabolic equivalents (METS) score), pre-pregnancy BMI (underweight, normal weight, overweight, obese), and participation in the following previous DNBC follow-up surveys: when the child was 6 m, 18 m and 7 years, respectively (‘yes’ to all vs. at least one ‘no’)) that may influence participation (orange) and one not accounting for these factors (blue). Values presented by a dot show data at 6 months follow-up whereas values presented by a triangle show data at 18 months follow-up.
IP: Inverse Probability, 95% CI: 95% Confidence Interval, SSB: Sugar sweetened beverages, FV: Fruits and vegetables, BMI: Body Mass Index, HEI: Healthy Eating Index, DNBC: Danish National Birth Cohort, y: Years, m: Months, SMS: Short Messages Service.
(TIF)

S5 Fig Main results for effects on mini-HEI and BMI z-scores, and SSB, FV and Fish z-scores, for females. Analyses of the two main outcomes, mini-HEI and BMI z-score, are shown in the upper and lower left panel, respectively, on each page; whereas analyses of the secondary outcomes, SSB, FV and Fish z-scores, are shown in the upper, middle and lower right panel, respectively. In each panel are shown analyses of the effect estimates for the continuous outcomes performed for AnySMS compared to Non-SMS (the estimate to the left in each panel); and for each of the three additional elements, i.e. adding mother compared to not adding mother, adding friend compared to not adding friend, or tailored SMS program compared to the full SMS program, assessed within the group of AnySMS, thus excluding the Non-SMS group (the three estimates to the right in each panel, respectively). Effect sizes are estimated differences between comparison groups in means of outcomes at 6 months and 18 months follow-up. Colors indicate effects estimated from intention-to-treat (ITT)-analyses including all individuals (black) and two Per Protocol-analyses excluding those who did not join the SMS-program that they were offered; one accounting for a set of factors (maternal pregnancy healthy eating index score (low, medium, high), smoking in pregnancy (yes, no), physical activity level in pregnancy (low, medium, high metabolic equivalents (METS) score), pre-pregnancy BMI (underweight, normal weight, overweight, obese), and participation in the following previous DNBC follow-up surveys: when the child was 6 m, 18 m and 7 years, respectively (‘yes’ to all vs. at least one ‘no’)) that may influence participation (orange) and one not accounting for these factors (blue). Values presented by a dot show data at 6 months follow-up whereas values presented by a triangle show data at 18 months follow-up.
IP: Inverse Probability, 95% CI: 95% Confidence Interval, SSB: Sugar sweetened beverages, FV: Fruits and vegetables, BMI: Body Mass Index, HEI: Healthy Eating Index, DNBC: Danish National Birth Cohort, y: Years, m: Months, SMS: Short Messages Service.
(TIF)

S6 Fig Main results for effects on mini-HEI and BMI z-scores, and SSB, FV and Fish z-scores, for age group 14y3m. Analyses of the two main outcomes, mini-HEI and BMI z-score, are shown in the upper and lower left panel, respectively, on each page; whereas analyses of the secondary outcomes, SSB, FV and Fish z-scores, are shown in the upper, middle and lower right panel, respectively. In each panel are shown analyses of the effect estimates for the continuous outcomes performed for AnySMS compared to Non-SMS (the estimate to the left in each panel); and for each of the three additional elements, i.e. adding mother compared to not adding mother, adding friend compared to not adding friend, or tailored SMS program compared to the full SMS program, assessed within the group of AnySMS, thus excluding the Non-SMS group (the three estimates to the right in each panel, respectively). Effect sizes are estimated differences between comparison groups in means of outcomes at 6 months and 18 months follow-up. Colors indicate effects estimated from intention-to-treat (ITT)-analyses including all individuals (black) and two Per Protocol-analyses excluding those who did not join the SMS-program that they were offered; one accounting for a set of factors (maternal pregnancy healthy eating index score (low, medium, high), smoking in pregnancy (yes, no), physical activity level in pregnancy (low, medium, high metabolic equivalents (METS) score), pre-pregnancy BMI (underweight, normal weight, overweight, obese), and participation in the following previous DNBC follow-up surveys: when the child was 6 m, 18 m and 7 years, respectively (‘yes’ to all vs. at least one ‘no’)) that may influence participation (orange) and one not accounting for these factors (blue). Values presented by a dot show data at 6 months follow-up whereas values presented by a triangle show data at 18 months follow-up.
IP: Inverse Probability, 95% CI: 95% Confidence Interval, SSB: Sugar sweetened beverages, FV: Fruits and vegetables, BMI: Body Mass Index, HEI: Healthy Eating Index, DNBC: Danish National Birth Cohort, y: Years, m: Months, SMS: Short Messages Service.
(TIF)

S7 Fig Main results for effects on mini-HEI and BMI z-scores, and SSB, FV and Fish z-scores, for age group 14y9m. Analyses of the two main outcomes, mini-HEI and BMI z-score, are shown in the upper and lower left panel, respectively, on each page; whereas analyses of the secondary outcomes, SSB, FV and Fish z-scores, are shown in the upper, middle and lower right panel, respectively. In each panel are shown analyses of the effect estimates for the continuous outcomes performed for AnySMS compared to Non-SMS (the estimate to the left in each panel); and for each of the three additional elements, i.e. adding mother compared to not adding mother, adding friend compared to not adding friend, or tailored SMS program compared to the full SMS program, assessed within the group of AnySMS, thus excluding the Non-SMS group (the three estimates to the right in each panel, respectively). Effect sizes are estimated differences between comparison groups in means of outcomes at 6 months and 18 months follow-up. Colors indicate effects estimated from intention-to-treat (ITT)-analyses including all individuals (black) and two Per Protocol-analyses excluding those who did not join the SMS-program that they were offered; one accounting for a set of factors (maternal pregnancy healthy eating index score (low, medium, high), smoking in pregnancy (yes, no), physical activity level in pregnancy (low, medium, high metabolic equivalents (METS) score), pre-pregnancy BMI (underweight, normal weight, overweight, obese), and participation in the following previous DNBC follow-up surveys: when the child was 6 m, 18 m and 7 years, respectively (‘yes’ to all vs. at least one ‘no’)) that may influence participation (orange) and one not accounting for these factors (blue). Values presented by a dot show data at 6 months follow-up whereas values presented by a triangle show data at 18 months follow-up.
IP: Inverse Probability, 95% CI: 95% Confidence Interval, SSB: Sugar sweetened beverages, FV: Fruits and vegetables, BMI: Body Mass Index, HEI: Healthy Eating Index, DNBC: Danish National Birth Cohort, y: Years, m: Months, SMS: Short Messages Service.
(TIF)

S8 Fig Main results for effects on mini-HEI and BMI z-scores, and SSB, FV and Fish z-scores, for age group 15y3m. Analyses of the two main outcomes, mini-HEI and BMI z-score, are shown in the upper and lower left panel, respectively, on each page; whereas analyses of the secondary outcomes, SSB, FV and Fish z-scores, are shown in the upper, middle and lower right panel, respectively. In each panel are shown analyses of the effect estimates for the continuous outcomes performed for AnySMS compared to Non-SMS (the estimate to the left in each panel); and for each of the three additional elements, i.e. adding mother compared to not adding mother, adding friend compared to not adding friend, or tailored SMS program compared to the full SMS program, assessed within the group of AnySMS, thus excluding the Non-SMS group (the three estimates to the right in each panel, respectively). Effect sizes are estimated differences between comparison groups in means of outcomes at 6 months and 18 months follow-up. Colors indicate effects estimated from intention-to-treat (ITT)-analyses including all individuals (black) and two Per Protocol-analyses excluding those who did not join the SMS-program that they were offered; one accounting for a set of factors (maternal pregnancy healthy eating index score (low, medium, high), smoking in pregnancy (yes, no), physical activity level in pregnancy (low, medium, high metabolic equivalents (METS) score), pre-pregnancy BMI (underweight, normal weight, overweight, obese), and participation in the following previous DNBC follow-up surveys: when the child was 6 m, 18 m and 7 years, respectively (‘yes’ to all vs. at least one ‘no’)) that may influence participation (orange) and one not accounting for these factors (blue). Values presented by a dot show data at 6 months follow-up whereas values presented by a triangle show data at 18 months follow-up.
IP: Inverse Probability, 95% CI: 95% Confidence Interval, SSB: Sugar sweetened beverages, FV: Fruits and vegetables, BMI: Body Mass Index, HEI: Healthy Eating Index, DNBC: Danish National Birth Cohort, y: Years, m: Months, SMS: Short Messages Service.
(TIF)

S9 Fig Main results for effects on mini-HEI and BMI z-scores, and SSB, FV and Fish z-scores, for baseline dietstratum 1 where the tailored SMS program targeted Fish. Analyses of the two main outcomes, mini-HEI and BMI z-score, are shown in the upper and lower left panel, respectively, on each page; whereas analyses of the secondary outcomes, SSB, FV and Fish z-scores, are shown in the upper, middle and lower right panel, respectively. In each panel are shown analyses of the effect estimates for the continuous outcomes performed for AnySMS compared to Non-SMS (the estimate to the left in each panel); and for each of the three additional elements, i.e. adding mother compared to not adding mother, adding friend compared to not adding friend, or tailored SMS program compared to the full SMS program, assessed within the group of AnySMS, thus excluding the Non-SMS group (the three estimates to the right in each panel, respectively). Effect sizes are estimated differences between comparison groups in means of outcomes at 6 months and 18 months follow-up. Colors indicate effects estimated from intention-to-treat (ITT)-analyses including all individuals (black) and two Per Protocol-analyses excluding those who did not join the SMS-program that they were offered; one accounting for a set of factors (maternal pregnancy healthy eating index score (low, medium, high), smoking in pregnancy (yes, no), physical activity level in pregnancy (low, medium, high metabolic equivalents (METS) score), pre-pregnancy BMI (underweight, normal weight, overweight, obese), and participation in the following previous DNBC follow-up surveys: when the child was 6 m, 18 m and 7 years, respectively (‘yes’ to all vs. at least one ‘no’)) that may influence participation (orange) and one not accounting for these factors (blue). Values presented by a dot show data at 6 months follow-up whereas values presented by a triangle show data at 18 months follow-up.
IP: Inverse Probability, 95% CI: 95% Confidence Interval, SSB: Sugar sweetened beverages, FV: Fruits and vegetables, BMI: Body Mass Index, HEI: Healthy Eating Index, DNBC: Danish National Birth Cohort, y: Years, m: Months, SMS: Short Messages Service.
(TIF)

S10 Fig Main results for effects on mini-HEI and BMI z-scores, and SSB, FV and Fish z-scores, for baseline dietstratum 2 where the tailored SMS program targeted FV. Analyses of the two main outcomes, mini-HEI and BMI z-score, are shown in the upper and lower left panel, respectively, on each page; whereas analyses of the secondary outcomes, SSB, FV and Fish z-scores, are shown in the upper, middle and lower right panel, respectively. In each panel are shown analyses of the effect estimates for the continuous outcomes performed for AnySMS compared to Non-SMS (the estimate to the left in each panel); and for each of the three additional elements, i.e. adding mother compared to not adding mother, adding friend compared to not adding friend, or tailored SMS program compared to the full SMS program, assessed within the group of AnySMS, thus excluding the Non-SMS group (the three estimates to the right in each panel, respectively). Effect sizes are estimated differences between comparison groups in means of outcomes at 6 months and 18 months follow-up. Colors indicate effects estimated from intention-to-treat (ITT)-analyses including all individuals (black) and two Per Protocol-analyses excluding those who did not join the SMS-program that they were offered; one accounting for a set of factors (maternal pregnancy healthy eating index score (low, medium, high), smoking in pregnancy (yes, no), physical activity level in pregnancy (low, medium, high metabolic equivalents (METS) score), pre-pregnancy BMI (underweight, normal weight, overweight, obese), and participation in the following previous DNBC follow-up surveys: when the child was 6 m, 18 m and 7 years, respectively (‘yes’ to all vs. at least one ‘no’)) that may influence participation (orange) and one not accounting for these factors (blue). Values presented by a dot show data at 6 months follow-up whereas values presented by a triangle show data at 18 months follow-up.
IP: Inverse Probability, 95% CI: 95% Confidence Interval, SSB: Sugar sweetened beverages, FV: Fruits and vegetables, BMI: Body Mass Index, HEI: Healthy Eating Index, DNBC: Danish National Birth Cohort, y: Years, m: Months, SMS: Short Messages Service.
(TIF)

S11 Fig Main results for effects on mini-HEI and BMI z-scores, and SSB, FV and Fish z-scores, for baseline dietstratum 3 where the tailored SMS program targeted SSB. Analyses of the two main outcomes, mini-HEI and BMI z-score, are shown in the upper and lower left panel, respectively, on each page; whereas analyses of the secondary outcomes, SSB, FV and Fish z-scores, are shown in the upper, middle and lower right panel, respectively. In each panel are shown analyses of the effect estimates for the continuous outcomes performed for AnySMS compared to Non-SMS (the estimate to the left in each panel); and for each of the three additional elements, i.e. adding mother compared to not adding mother, adding friend compared to not adding friend, or tailored SMS program compared to the full SMS program, assessed within the group of AnySMS, thus excluding the Non-SMS group (the three estimates to the right in each panel, respectively). Effect sizes are estimated differences between comparison groups in means of outcomes at 6 months and 18 months follow-up. Colors indicate effects estimated from intention-to-treat (ITT)-analyses including all individuals (black) and two Per Protocol-analyses excluding those who did not join the SMS-program that they were offered; one accounting for a set of factors (maternal pregnancy healthy eating index score (low, medium, high), smoking in pregnancy (yes, no), physical activity level in pregnancy (low, medium, high metabolic equivalents (METS) score), pre-pregnancy BMI (underweight, normal weight, overweight, obese), and participation in the following previous DNBC follow-up surveys: when the child was 6 m, 18 m and 7 years, respectively (‘yes’ to all vs. at least one ‘no’)) that may influence participation (orange) and one not accounting for these factors (blue). Values presented by a dot show data at 6 months follow-up whereas values presented by a triangle show data at 18 months follow-up.
IP: Inverse Probability, 95% CI: 95% Confidence Interval, SSB: Sugar sweetened beverages, FV: Fruits and vegetables, BMI: Body Mass Index, HEI: Healthy Eating Index, DNBC: Danish National Birth Cohort, y: Years, m: Months, SMS: Short Messages Service.
(TIF)

S12 Fig Main results for effects on mini-HEI and BMI z-scores, and SSB, FV and Fish z-scores, for baseline dietstratum 4 where the tailored SMS program (also) targeted Fish. Analyses of the two main outcomes, mini-HEI and BMI z-score, are shown in the upper and lower left panel, respectively, on each page; whereas analyses of the secondary outcomes, SSB, FV and Fish z-scores, are shown in the upper, middle and lower right panel, respectively. In each panel are shown analyses of the effect estimates for the continuous outcomes performed for AnySMS compared to Non-SMS (the estimate to the left in each panel); and for each of the three additional elements, i.e. adding mother compared to not adding mother, adding friend compared to not adding friend, or tailored SMS program compared to the full SMS program, assessed within the group of AnySMS, thus excluding the Non-SMS group (the three estimates to the right in each panel, respectively). Effect sizes are estimated differences between comparison groups in means of outcomes at 6 months and 18 months follow-up. Colors indicate effects estimated from intention-to-treat (ITT)-analyses including all individuals (black) and two Per Protocol-analyses excluding those who did not join the SMS-program that they were offered; one accounting for a set of factors (maternal pregnancy healthy eating index score (low, medium, high), smoking in pregnancy (yes, no), physical activity level in pregnancy (low, medium, high metabolic equivalents (METS) score), pre-pregnancy BMI (underweight, normal weight, overweight, obese), and participation in the following previous DNBC follow-up surveys: when the child was 6 m, 18 m and 7 years, respectively (‘yes’ to all vs. at least one ‘no’)) that may influence participation (orange) and one not accounting for these factors (blue). Values presented by a dot show data at 6 months follow-up whereas values presented by a triangle show data at 18 months follow-up.
IP: Inverse Probability, 95% CI: 95% Confidence Interval, SSB: Sugar sweetened beverages, FV: Fruits and vegetables, BMI: Body Mass Index, HEI: Healthy Eating Index, DNBC: Danish National Birth Cohort, y: Years, m: Months, SMS: Short Messages Service.
(TIF)

S13 Fig Main results for effects on mini-HEI and BMI z-scores, and SSB, FV and Fish z-scores, for those defined to be underweight or normal weight (85% limit). Analyses of the two main outcomes, mini-HEI and BMI z-score, are shown in the upper and lower left panel, respectively, on each page; whereas analyses of the secondary outcomes, SSB, FV and Fish z-scores, are shown in the upper, middle and lower right panel, respectively. In each panel are shown analyses of the effect estimates for the continuous outcomes performed for AnySMS compared to Non-SMS (the estimate to the left in each panel); and for each of the three additional elements, i.e. adding mother compared to not adding mother, adding friend compared to not adding friend, or tailored SMS program compared to the full SMS program, assessed within the group of AnySMS, thus excluding the Non-SMS group (the three estimates to the right in each panel, respectively). Effect sizes are estimated differences between comparison groups in means of outcomes at 6 months and 18 months follow-up. Colors indicate effects estimated from intention-to-treat (ITT)-analyses including all individuals (black) and two Per Protocol-analyses excluding those who did not join the SMS-program that they were offered; one accounting for a set of factors (maternal pregnancy healthy eating index score (low, medium, high), smoking in pregnancy (yes, no), physical activity level in pregnancy (low, medium, high metabolic equivalents (METS) score), pre-pregnancy BMI (underweight, normal weight, overweight, obese), and participation in the following previous DNBC follow-up surveys: when the child was 6 m, 18 m and 7 years, respectively (‘yes’ to all vs. at least one ‘no’)) that may influence participation (orange) and one not accounting for these factors (blue). Values presented by a dot show data at 6 months follow-up whereas values presented by a triangle show data at 18 months follow-up.
IP: Inverse Probability, 95% CI: 95% Confidence Interval, SSB: Sugar sweetened beverages, FV: Fruits and vegetables, BMI: Body Mass Index, HEI: Healthy Eating Index, DNBC: Danish National Birth Cohort, y: Years, m: Months, SMS: Short Messages Service.
(TIF)

S14 Fig Main results for effects on mini-HEI and BMI z-scores, and SSB, FV and Fish z-scores, for those defined to be overweight (85% limit). Analyses of the two main outcomes, mini-HEI and BMI z-score, are shown in the upper and lower left panel, respectively, on each page; whereas analyses of the secondary outcomes, SSB, FV and Fish z-scores, are shown in the upper, middle and lower right panel, respectively. In each panel are shown analyses of the effect estimates for the continuous outcomes performed for AnySMS compared to Non-SMS (the estimate to the left in each panel); and for each of the three additional elements, i.e. adding mother compared to not adding mother, adding friend compared to not adding friend, or tailored SMS program compared to the full SMS program, assessed within the group of AnySMS, thus excluding the Non-SMS group (the three estimates to the right in each panel, respectively). Effect sizes are estimated differences between comparison groups in means of outcomes at 6 months and 18 months follow-up. Colors indicate effects estimated from intention-to-treat (ITT)-analyses including all individuals (black) and two Per Protocol-analyses excluding those who did not join the SMS-program that they were offered; one accounting for a set of factors (maternal pregnancy healthy eating index score (low, medium, high), smoking in pregnancy (yes, no), physical activity level in pregnancy (low, medium, high metabolic equivalents (METS) score), pre-pregnancy BMI (underweight, normal weight, overweight, obese), and participation in the following previous DNBC follow-up surveys: when the child was 6 m, 18 m and 7 years, respectively (‘yes’ to all vs. at least one ‘no’)) that may influence participation (orange) and one not accounting for these factors (blue). Values presented by a dot show data at 6 months follow-up whereas values presented by a triangle show data at 18 months follow-up.
IP: Inverse Probability, 95% CI: 95% Confidence Interval, SSB: Sugar sweetened beverages, FV: Fruits and vegetables, BMI: Body Mass Index, HEI: Healthy Eating Index, DNBC: Danish National Birth Cohort, y: Years, m: Months, SMS: Short Messages Service.
(TIF)

S15 Fig Main results for effects on mini-HEI and BMI z-scores, and SSB, FV and Fish z-scores, for those who had an unhealthy diet (HEI below zero) at baseline. Analyses of the two main outcomes, mini-HEI and BMI z-score, are shown in the upper and lower left panel, respectively, on each page; whereas analyses of the secondary outcomes, SSB, FV and Fish z-scores, are shown in the upper, middle and lower right panel, respectively. In each panel are shown analyses of the effect estimates for the continuous outcomes performed for AnySMS compared to Non-SMS (the estimate to the left in each panel); and for each of the three additional elements, i.e. adding mother compared to not adding mother, adding friend compared to not adding friend, or tailored SMS program compared to the full SMS program, assessed within the group of AnySMS, thus excluding the Non-SMS group (the three estimates to the right in each panel, respectively). Effect sizes are estimated differences between comparison groups in means of outcomes at 6 months and 18 months follow-up. Colors indicate effects estimated from intention-to-treat (ITT)-analyses including all individuals (black) and two Per Protocol-analyses excluding those who did not join the SMS-program that they were offered; one accounting for a set of factors (maternal pregnancy healthy eating index score (low, medium, high), smoking in pregnancy (yes, no), physical activity level in pregnancy (low, medium, high metabolic equivalents (METS) score), pre-pregnancy BMI (underweight, normal weight, overweight, obese), and participation in the following previous DNBC follow-up surveys: when the child was 6 m, 18 m and 7 years, respectively (‘yes’ to all vs. at least one ‘no’)) that may influence participation (orange) and one not accounting for these factors (blue). Values presented by a dot show data at 6 months follow-up whereas values presented by a triangle show data at 18 months follow-up.
IP: Inverse Probability, 95% CI: 95% Confidence Interval, SSB: Sugar sweetened beverages, FV: Fruits and vegetables, BMI: Body Mass Index, HEI: Healthy Eating Index, DNBC: Danish National Birth Cohort, y: Years, m: Months, SMS: Short Messages Service.
(TIF)

S16 Fig Main results for effects on mini-HEI and BMI z-scores, and SSB, FV and Fish z-scores, for those who had a healthy diet (HEI above zero) at baseline. Analyses of the two main outcomes, mini-HEI and BMI z-score, are shown in the upper and lower left panel, respectively, on each page; whereas analyses of the secondary outcomes, SSB, FV and Fish z-scores, are shown in the upper, middle and lower right panel, respectively. In each panel are shown analyses of the effect estimates for the continuous outcomes performed for AnySMS compared to Non-SMS (the estimate to the left in each panel); and for each of the three additional elements, i.e. adding mother compared to not adding mother, adding friend compared to not adding friend, or tailored SMS program compared to the full SMS program, assessed within the group of AnySMS, thus excluding the Non-SMS group (the three estimates to the right in each panel, respectively). Effect sizes are estimated differences between comparison groups in means of outcomes at 6 months and 18 months follow-up. Colors indicate effects estimated from intention-to-treat (ITT)-analyses including all individuals (black) and two Per Protocol-analyses excluding those who did not join the SMS-program that they were offered; one accounting for a set of factors (maternal pregnancy healthy eating index score (low, medium, high), smoking in pregnancy (yes, no), physical activity level in pregnancy (low, medium, high metabolic equivalents (METS) score), pre-pregnancy BMI (underweight, normal weight, overweight, obese), and participation in the following previous DNBC follow-up surveys: when the child was 6 m, 18 m and 7 years, respectively (‘yes’ to all vs. at least one ‘no’)) that may influence participation (orange) and one not accounting for these factors (blue). Values presented by a dot show data at 6 months follow-up whereas values presented by a triangle show data at 18 months follow-up.
IP: Inverse Probability, 95% CI: 95% Confidence Interval, SSB: Sugar sweetened beverages, FV: Fruits and vegetables, BMI: Body Mass Index, HEI: Healthy Eating Index, DNBC: Danish National Birth Cohort, y: Years, m: Months, SMS: Short Messages Service.
(TIF)

## Abbreviations

BMI: body mass index
CACE: complier average causal effect
CI: confidence interval
DNBC: Danish National Birth Cohort
FFQ: food frequency questionnaire
FV: fruit and vegetables
GEE: generalized estimating equation
HEI: Healthy Eating Index
IPW-PP: inverse probability weighted per-protocol
ITT: intention-to-treat
PP: per-protocol
RCT: randomized controlled trial
RR: risk ratio
SAP: Statistical Analysis Plan
SMS: Short Messages Service
SSB: sugar-sweetened beverage
