# Supplementary material for: An SMS chatbot digital educational program to increase healthy eating behaviors in adolescence: A multifactorial randomized controlled trial among 7,890 participants in the Danish National Birth Cohort
Source: PLoS Med. 2024 Jun 14;21(6):e1004383. doi: 10.1371/journal.pmed.1004383 (PMC11178212; doi:10.1371/journal.pmed.1004383)
Supplement: S1 Text — 1 Duration, structure and content of the SMS messaging programs. 2 Definition of pre-randomization strata (more detailed and concise descriptions can be provided). 3 Ethical and scientific considerations underlying the choice of the program’s three target factors: Lowering the intake of sugar sweetened beverages, increasing the intake of fruit and vegetables, and increasing the intake of fish. (DOCX) [file pmed.1004383.s001.docx]

**Supporting Information 1**

**Content:**

1. Duration, structure and content of the SMS messaging programs.
2. Definition of pre-randomization strata (more detailed and concise descriptions can be provided).
3. Ethical and scientific considerations underlying the choice of the program’s three target factors: Lowering the intake of sugar sweetened beverages, increasing the intake of fruit and vegetables, and increasing the intake of fish.
4. **Duration, structure and content of the SMS messaging programs**

The FULL program comprised messages distributed over 12 weeks, whereas each of the three other programs, FISH, fruits and vegetables (FV), and sugar-sweetened beverages (SSB), each comprised messages distributed over 4 weeks. In all four programs, messages belonged to one (or more) of five different categories: Information, quizzes, tips, challenges, and reminders. Information messages included a “Welcome and start up message” and a final message saying “Goodbye and thanks for participating”. The FULL program also contained one message that encouraged the participant to carry on after the program ended and information about winning two cinema tickets. The quiz messages constituted the majority of the messages and covered overall dietary recommendations, as well as recommendations on FISH, FV, and/or SSB (depending on the program). The messages were designed in a way such that the answer to a question was put together with a new question, tip, challenge or reminder. Reminder messages referred to previously received quiz or challenge messages. The FULL program consisted of 188 quiz/tips/reminder messages: 23 messages on SSB, 26 messages on FV, 24 messages on fish, 19 messages on physical activity, three messages on dietary fibers, and 20 messages about meals (referring to snacking, breakfast, lunch and distribution of macronutrients in a meal); in addition, there were four information messages and two challenges. The FISH program consisted of 37 quiz/tips/reminder messages: 25 messages on fish and 12 messages about meals; in addition, there were two information messages and two challenges. The FV program consisted of 33 quiz/tips/reminder messages: 31 messages on FV, two messages on FV/SSB, and 6 messages about meals; in addition, there were two information messages and one challenge. The SSB program consisted of 36 quiz/tips/reminder messages: 21 messages on SSB, three messages on SSB/FV, and 12 about meals; in addition, there were two information messages and one challenge.

1. **Definition of pre-randomization strata (more detailed and concise descriptions can be provided)**

*Dietary Risk Stratum I:* All participants consume fish below the recommendation. All consume SSB and FV corresponding to the respective recommendations. The dietary factor addressed in the targeted educational program shown in **Table 1** (i.e., “1 DF” in the table) was: Fish.

*Dietary Risk Stratum II:* All participants consume FV below the recommendation. All consumed SSB corresponding to the recommendation. Nearly all consume fish below the recommendation. The dietary factor addressed in the targeted educational program shown in **Table 1** (i.e., “1 DF” in the table) was: FV.

*Dietary Risk Stratum III:* All participants consume SSB above the recommendation. Nearly all consume FV corresponding to the recommendation. Nearly all consume fish below the recommendation. The dietary factor addressed in the targeted educational program shown in **Table 1** (i.e., “1 DF” in the table) was: SSB.

*Dietary Risk Stratum IV:* Problems with all three diets/dietary behaviors. All participants consume fish below the recommendation. All consume SSB above the recommendation. All consume FV below the recommendation. The dietary factor addressed in the targeted educational program shown in **Table 1** (i.e., “1 DF” in the table) was: Fish.

1. **Ethical and scientific considerations underlying the choice of the program’s three target factors: Lowering the intake of sugar sweetened beverages, increasing the intake of fruit and vegetables, and increasing the intake of fish.**

Below is the justification for our choice to target these particular dietary factors / dietary behaviors. According to scientific evidence available in the literature, summarized below, at the time of the planning of the study, the changes in dietary habits aimed for in the program would most likely be beneficial – and in the worst case scenario, at least not be harmful – to the participants’ health. Consequently, it was our judgment that participation in an SMS educational program targeting these factors would not involve more than "minimal risk", as defined in the US Federal Regulations. According to these regulations, "minimal risk" means that "the probability and magnitude of harm or discomfort anticipated in the research are not greater in and of themselves than those ordinarily encountered in daily life or during the performance of routine physical or psychological examinations or tests ([45 CFR 46.102(i)] and [21 CFR Part 56.102(i)]).

*Sugar sweetened beverages:* In general, Danish schoolchildren surpass the recommendations of maximum 10E%/day from sugar, corresponding to 45-50 g/day (1). The underlying food item for the high sugar consumption is sugar-sweetened beverages (SSB),(2) but also candy, snack, cakes etc. contribute with high amounts of sugar. A recent dietary survey showed that 77% of children aged 4-18 years consumed more than the recommended 0.5 l SSB per week (4). For the 15–18-year-olds, a peak in intake of SSB has been identified with an average intake of 1.8l/week. 80-90% of all children and adolescents, consumed more sugar than recommended mainly due to intake of SSB (1). The high concentration of sugar in SSB, although not providing the feeling of fullness like solid food, may lead to an increase in energy intake, which can lead to weight gain (3). Furthermore, high consumption of SSB has also been associated with low bone mass accrual and increased bone fracture risk among adolescents (4–7). An American prospective study including 548 children aged 11-12 years found a 60% increased risk of developing obesity when just a can (380 ml) of soda were consumed on a daily basis (8). In a Danish randomized intervention study including 47 adults, there was an association between high intake of SSB and accumulation of fat in liver, muscles and abdomen after 6 months of intervention with an intake of 1 liter of SSB per day (9). In this intervention, the daily intake of SSB was relatively high, however the study revealed the importance of lowering the intake of SSB among children and adolescents to prevent later obesity. This has also been demonstrated in two relatively large trials (n=224 and n=641) on SSB in children and adolescents with effect on body weight at 12 and 18 months follow-up (10,11), although this was not confirmed in a Danish intervention study among 269 children which could be due follow-up of 3 to 7 years (12).

*Fruit and vegetables:* The Danish recommendations for adolescents with respect to intake of fruit and vegetables are 600 g per day of which minimum 50% should be vegetables (13,14), but the majority of Danish adolescents does not meet those recommendations. In the International Health Behavior in School – aged Children (HBSC) study the consumption of fruit was measured at six different time-points from 1988 to 2006 in Danish school children (n=23,871). Overall, the consumption of fruit decreased from 1988 to 2002 and in 2002, only 35.8% of the girls (n=2,476) and 17.3% of the boys (n=2,348) (15 years of age) consumed at least one piece of fruit per day. The percentages consuming at least one piece of fruit per day increased and in 2006 it was 63.6% and 41.3% for girls and boys, respectively (15). The increase since 2002 may be due to a nation-wide initiative commenced in 2001 with the purpose to increase intake of fruit and vegetables in the Danish population. However, the national recommendations for intake of fruit and vegetables were not met in 2006 for the majority of the children (15). The low percentages meeting recommendations are in accordance with another study from the European 2003 Pro Children Survey (EPCS), including 1410 Danish schoolchildren. In this study, intake of fruit and vegetables was measured among 11-year old schoolchildren from nine different European countries (16). Among the Danish children participating, 58% of girls (n=683) and 63.3% of boys (n=660) consumed less than 200 g fruit per day, and 72,2% of the girls and 77.7% of the boys had a vegetable intake below 130 g day, which is far below the recommendations (17). Data from the Danish National Survey of Dietary Habits and Physical Activity (DSDH,

, The DSDH-2003-8, showed that the median intake (10%, 90% percentile) of fruit and vegetables was 338 (115, 771) g per day in 595 children aged 10-17 years, which substantiates that the recommendations for intake of fruit and vegetables are not met for a majority of Danish children (18). A meta-analysis has shown a positive effect against stroke by consuming fruit and vegetable. More than three servings of fruit and vegetables per day was associated with a reduced risk of stroke compared with individuals who had less. Three to four servings per day reduced the risk by 11%, and more than five servings per day were associated with a reduction of 26% in the risk of stroke (19). These findings support the Danish recommendation. Fruit and vegetables are the food group contributing the most vitamins and minerals compared to the amount of energy (20). They are important sources of dietary flavonoids, which has been shown to have a health benefits towards cardiovascular diseases and hypertension (21). Furthermore, fruit and vegetables in general have a high content of dietary fiber. Studies indicate that adults significantly lower the risk of obesity, diabetes and constipation with higher dietary fiber consumption (22). However, there is a lack of studies investigating this in children (23). A recent study has further analyzed the association between dietary fiber consumption in children and their cognitive performance. Interestingly, the study demonstrated that dietary fiber consumption was positively associated with cognitive performance in 7-9 year old children (n=65) (24).

*Fish:* The recommendations for children and adolescents with respect to intake of seafood are 350 g per week of which 200 g should be fatty fish (13,14). A majority of Danish children does not meet those recommendations. Data from the DSDH-2003-8 showed that the median intake (10%, 90% percentile) of seafood was 7.0 (0, 28) g/day in 595 children aged 10-17 years (18). The low intake of seafood among children and adolescents is of concern, since seafood is the main dietary source of vitamin D. In the DSDH-2003-8 it was estimated that the median intake of vitamin D in children was less than 2 µg/day, and in adults 2.5 µg/day (18). This is far below the recommendations of 10 µg/day (25). Marine food is the principal source of the long chain n-3 fatty acids, docosahexaenoic acid (DHA) and eicosapentaenoic acid (EPA), which are believed to be important for brain and eye development and to protect the cardiovascular system. Other important nutrients of seafood include selenium, iodine, magnesium, iron, copper and high-quality proteins, which all are important for child growth and development (26).

**References**

1. Matthiessen J, Groth M, Fagt S. Kostens betydning for børns sundhed og overvægt. DTU Fødevareinstituttet. 2013. E-artikel ISSN 1904-5581

2. Fagt S. Udvikling i uregelmæssige måltider og indtag of fast-food blandt børn og unge. DTU Fødevareinstituttet. 2008. E-artikel ISSN 1904-5581

3. World Health Organization (WHO). Reducing consumption of sugar-sweetened beverages to reduce the risk of childhood overweight and obesity - GUIDELINE “SUGAR INTAKE FOR ADULTS AND CHILDREN" 2015.

4. Wyshak G. Teenaged girls, carbonated beverage consumption, and bone fractures. Arch Pediatr Adolesc Med. 2000;154(6).

5. Ma D, Jones G. Soft drink and milk consumption, physical activity, bone mass, and upper limb fractures in children: A population-based case-control study. Calcif Tissue Int. 2004;75(4).

6. McGartland C, Robson PJ, Murray L, Cran G, Savage MJ, Watkins D, et al. Carbonated soft drink consumption and bone mineral density in adolescence: The Northern Ireland young hearts project. J Bone Miner Res. 2003;18(9).

7. Libuda L, Alexy U, Remer T, Stehle P, Schoenau E, Kersting M. Association between long-term consumption of soft drinks and variables of bone modeling and remodeling in a sample of healthy German children and adolescents. Am J Clin Nutr. 2008;88(6).

8. Ludwig DS, Peterson KE, Gortmaker SL. Relation between consumption of sugar-sweetened drinks and childhood obesity: A prospective, observational analysis. Lancet. 2001;357(9255).

9. Maersk M, Belza A, Stødkilde-Jørgensen H, Ringgaard S, Chabanova E, Thomsen H, et al. Sucrose-sweetened beverages increase fat storage in the liver, muscle, and visceral fat depot: A 6-mo randomized intervention study. Am J Clin Nutr. 2012;95(2).

10. Ebbeling CB, Feldman HA, Chomitz VR, Antonelli TA, Gortmaker SL, Osganian SK, et al. A Randomized Trial of Sugar-Sweetened Beverages and Adolescent Body Weight. N Engl J Med. 2012;367(15).

11. de Ruyter JC, Olthof MR, Seidell JC, Katan MB. A Trial of Sugar-free or Sugar-Sweetened Beverages and Body Weight in Children. N Engl J Med. 2012;367(15).

12. Jensen BW, Nielsen BM, Husby I, Bugge A, El-Naaman B, Andersen LB, et al. Association between sweet drink intake and adiposity in Danish children participating in a long-term intervention study. Pediatr Obes. 2013;8(4).

13. Danish Veterinary and Food Administration. The Official Dietary Guidelines – good for health and climate [Internet]. [accessed 2023 Nov 30]. Available from: The Official Dietary Guidelines – good for health and climate

14. Tetens I, Andersen LB, Astrup A, Gondolf UH, Hermansen K, Jakobsen MU, et al. Evidensgrundlaget for danske råd om kost og fysisk aktivitet. DTU Fødevareinstituttet. 2013. ISBN (print) 978-87-92763-96-9

15. Rasmussen M, Krølner R, Svastisalee C, Due P, Holstein B. Secular trends in fruit intake among Danish schoolchildren, 1988 to 2006: Changing habits or methodological artefacts? Int J Behav Nutr Phys Act. 2008;5.

16. Klepp KI, Pérez-Rodrigo C, De Bourdeaudhuij I, Due P, Elmadfa I, Haraldsdóttir J, et al. Promoting fruit and vegetable consumption among European schoolchildren: Rationale, conceptualization and design of the Pro Children Project. Vol. 49, Annals of Nutrition and Metabolism. 2005.

17. Krølner R, Due P, Rasmussen M, Damsgaard MT, Holstein BE, Klepp KI, et al. Does school environment affect 11-year-olds’ fruit and vegetable intake in Denmark? Soc Sci Med. 2009;68(8).

18. Pedersen AN, Fagt S, Groth M V, Christensen T, Biltoft-Jensen A, Matthiessen J, et al. Danskernes kostvaner 2003-2008. DTU Fødevareinstituttet. 2010. ISBN (print) 978-87-92158-67-3

19. He FJ, Nowson CA, MacGregor GA. Fruit and vegetable consumption and stroke: Meta-analysis of cohort studies. Lancet. 2006;367(9507).

20. Hallund JD, Halkjær J, Madsen C, Ovesen L, Rasmussen H., Tetens I, et al. Frugt, grøntsager og sundhed. Opdatering af vidensgrundlaget for mængdeanbefalinger 2002-2006. 2007.

21. Kozlowska K. Functional somatic symptoms in childhood and adolescence. Curr Opin Psychiatry [Internet]. 2013;26(5):485–92. Available from: http://www.ncbi.nlm.nih.gov/pubmed/23867659

22. Otles S, Ozgoz S. Health effects of dietary fiber. Acta Sci Pol Technol Aliment . 2014;13(2):191–202.

23. Kranz S, Brauchla M, Slavin JL, Miller KB. What do we know about dietary fiber intake in children and health? The effects of fiber intake on constipation, obesity, and diabetes in children. Vol. 3, Advances in Nutrition. 2012.

24. Khan NA, Raine LB, Drollette ES, Scudder MR, Kramer AF, Hillman CH. Dietary fiber is positively associated with cognitive control among prepubertal children. J Nutr. 2015;145(1).

25. Nordic Counsil of Ministers. Nordic Nutrition Recommendations - Integrating nutrition and physical activity. Vol. 5. 2012. 349–350 p.

26. Nunes E, Cavaco A, Carvalho C. Children’s health risk and benefits of fish consumption: Risk indices based on a diet diary follow-up of two weeks. J Toxicol Environ Heal - Part A Curr Issues. 2014;77(1–3).
