# Supplementary material for: An SMS chatbot digital educational program to increase healthy eating behaviors in adolescence: A multifactorial randomized controlled trial among 7,890 participants in the Danish National Birth Cohort
Source: PLoS Med. 2024 Jun 14;21(6):e1004383. doi: 10.1371/journal.pmed.1004383 (PMC11178212; doi:10.1371/journal.pmed.1004383)
Supplement: S2 Text — (PDF) [file pmed.1004383.s002.pdf]

Statistical Analysis Plan (SAP) for

Texts For Healthy Teens: A Health Education Program for Adolescents (T4HT)

Registered at ClinicalTrials.Gov with ID NCT02809196

SAP version 2.0; June 17, 2019

This document lays out the analysis plan prior to decoding the trial data.

Preliminary analyses have been undertaken *a priori* in connection with the development of statistical models and statistical power considerations. However, until this point those analyses have been done solely among the null control group receiving no SMS messaging.

Content

1. The main questions that the trial will seek to answer
2. Methodological issues
3. Primary analyses testing the main hypotheses
4. Catalogue of planned analyses addressing various additional questions

1. The main questions that the trial will seek to answer

The overall objective is to examine if, in a 2x2x2+1 design participating in an SMS-transmitted health promotion program targeting a cluster of dietary factors over a period of a few weeks during the years of adolescence can

1) induce durable changes towards more healthful dietary habits, as expressed by change in a Healthy Eating Index, and

2) reduce the propensity for developing overweight, as expressed by change in mean BMI z-score, and the probability of exceeding the 85% percentile of BMI Z-for-age-for-gender at follow up.

These hypotheses may (e.g., if a specific change in diet induced by the SMS-program leads to a change in overweight propensity), or may not, be related and will from the outset be tested independently of each other.

The corresponding main specific aims, are to evaluate the following interventions and outcomes

1) OUTCOME 1: Examine if changes towards more healthful dietary habits, as expressed by change in a Healthy Eating Index, can be detected among the adolescents at follow up at 6 (FU1) and 18 months (FU2) after the initiation of the SMS-transmitted health promotion program; i.e.

- **ANY SMS DIETARY EDUCATION PROGRAM vs. NO-SMS NULL GROUP:** Examine if any of the SMS programs together are superior to a fully null group without any SMS or active education.

Whereas, the employed factorial recruitment schedule will enable us to evaluate the outcome with respect to these INTERVENTIONS:

- **SMS FRIEND vs. NO-FRIEND effect (+F,-F):** Examine if an adolescent receiving a standard SMS dietary education program that is reinforced by asking a friend of the participant to also concurrently participate in a corresponding SMS-transmitted health promotion program is more effective than a program not reinforced by a friend on effects of the specific outcomes.
- **SMS MOTHER vs. NO MOTHER effect (+M,-M):** Examine if an adolescent receiving a standard SMS dietary education program that is reinforced by asking the mother of the participant to also concurrently participate in a corresponding SMS-transmitted health promotion program summing up main points from the adolescents program is more effective than a program not reinforced by a mother on effects of the specific outcomes.; and
SMS TAILORED vs. NO TAILORED (STANDARD) EDUCATION PROGRAM effect (+T,-T): Examine if an adolescent receiving an SMS education program that *targets a specific dietary factor*, is as effective as compared to an SMS education program generically designed to target a standard mixed education of various dietary factor.

2) OUTCOME 2: Examine if changes towards reduced propensity for overweight development , as expressed by change in mean BMI z-score and % exceeding 85% percentile, can be detected among the adolescents follow up at 6 (FU1) and 18 months (FU2) after the initiation of the SMS-transmitted health promotion program; i.e.

- **ANY SMS DIETARY EDUCATION PROGRAM vs. NO-SMS NULL GROUP:** Examine if any of the SMS programs together are superior to a fully null group without any SMS or active education.

Whereas, the employed factorial recruitment schedule will further enable us to evaluate the outcome with respect to these INTERVENTIONS:

- **SMS FRIEND vs. NO-FRIEND effect (+F, -F):** Examine if an adolescent receiving a standard SMS dietary education program that is reinforced by asking a friend of the participant to also concurrently participate in a corresponding SMS-transmitted health promotion program is more effective than a program not reinforced by a friend on effects of the specific outcomes.
- **SMS MOTHER vs. NO MOTHER effect (+M,-M):** Examine if an adolescent receiving a standard SMS dietary education program that is reinforced by asking the mother of the participant to also concurrently participate in a corresponding SMS-transmitted health promotion program summing up main points from the adolescents program; and
- **SMS TAILORED vs. NO TAILORED (STANDARD) EDUCATION PROGRAM effect (+T,-T):** Examine if an adolescent receiving an SMS education program that *targets a specific dietary factor*, is as effective as compared to an SMS education program generically designed to target a standard mixed education of various dietary factor.

2. Methodological issues

Definition of outcome in the statistical models:

Data from the null control group suggest that the logarithm of the dietary intake of SSB, fruit & vegetables, and fish at FU1 and FU2 (6 and 18 months after the initiation of the SMS-transmitted health promotion program) are all approximately normally distributed. Therefore, we will model the log-transformed dietary intake values at relevant follow-up to be normally distributed.

To address outcome 1, the mini-HEI index will be calculated as the average of z-scores (using means and standard deviations from the null control group) of the three log-transformed dietary intake values (SSB, fruit & vegetables and fish; low SSB-, high fruit and vegetables- and high fish intake will is regarded as healthy eating).

In analyses estimating the effect of the SMS-transmitted health promotion program on single dietary intake variables; SSB, fruit & vegetables, and fish, respectively, the effect estimates will be interpreted as percentage difference (increase or decrease) in intake at FU1, FU2, or at both time points in intervention groups compared to fully null group without any intervention.

To address outcome 2, the BMI z-scores among the individuals in the null control group is approximately normally distributed, so no further transformation will be made in the analysis where BMI z-score is the outcome. The standards published by CDC will be used for defining overweight https://www.cdc.gov/obesity/childhood/defining.html .

The two effects at FU1 and FU2 will be adjusted for the corresponding value at baseline (linear effect) and the full combination of gender, age group and strata that determined randomization (factor with 24 levels). Repeated measures will be accounted for by modeling the two follow-up measurements from an individual to be correlated. Effects of the different interventions will be tested by joint tests of effects at FU1 and FU2.

Models for assessing main effects:

The effect of participating in the SMS programs can in principle be defined and modelled in several different ways. There will technically be at least four options available for defining and estimating the effect of SMS v. the null control group:

Option 1. “SMS Basic” effect: compare only those receiving SMS(-F,-M,-T) v. null control group

Option 2. “SMS de luxe” effect: compare only those receiving SMS(+F,+M,+T) v. null control group.

Option 3. Overall (crude) SMS effect: compare all receiving SMS (irrespective of friend or mother) v. null control group.

Option 4. SMS effect after adjustment for any “friend-effect”, any “mother-effect”, and any tailoring-effect: compare all receiving SMS v. null control group, but adjust for “friend-effect”, “mother-effect”, and “tailoring-effect” by including these three variables in a multivariable model.

Option 3 will be the preferred model. The 2*2*2 design within the group receiving the SMS intervention will enable estimation of possible separate effects of adding a friend, of adding the mother, and of tailoring the to the SMS program.

Models for adjusting for differential participation in the follow up questionnaire and in the offered SMS programs:

We anticipate that the planned adjustment in three models, detailed below, will to some extent adjust for differential participation in the follow up questionnaire and in the offered SMS programs.

Adjustment in Model I:

We will, for the main analysis, adjust for the corresponding baseline value of the outcome and for the combination of gender (male, female), age groups (years at baseline 14.3, 14.9, or 15.3), and four strata that determined randomization (1: low intake of fish, fruits and vegetables, and high intake of SSB, 2: high intake of SSB, 3: low intake of fruits and vegetables, and 4: low intake of fish); this will be Model I results.

Adjustment in Model II:

To further adjust for factors which may determine participation in the follow up questionnaire and in the offered SMS programs, we will further estimate a propensity of participation determined variables available in the DNBC database assessed during pregnancy. These will include maternal pregnancy healthy eating index score (low, medium, high), smoking in pregnancy (yes, no), physical activity level in pregnancy (low, medium, high METS score), pre-pregnancy BMI (under-, normal-, overweight, obese), and participation in the following previous DNBC follow up surveys: when the child was 6 months, 18 months, 7 years and 11 years old, respectively (yes, no); we will then both weigh (IPW) the observations and adjust for the estimated propensity in the analyses: this will be Model II results.

Adjustment in Model III:

To further adjust for factors which may determine participation in the follow up questionnaire and in the offered SMS programs, we will further include parental education, parental employment, and family income, which are variables available by linkage to Denmark Statistics registers to the estimation of propensity of participation; this will be Model III results.

3. **Primary analyses testing the main hypotheses**

This first part of the analysis plan uses data from all participants. The model corresponding to Option 3 in the “Methodological Issues” section will be used for testing effect of SMS-messaging.

Primary Hypothesis 1: Compared to adolescents offered no SMS-program (null control group), adolescents offered any SMS-program will report dietary intakes resulting in *increased* mini-HEI assessed at, either 6m, 18m, or at both points in time by a joint test.

Primary Hypothesis 2: Compared to adolescents offered no SMS-program (null control group), adolescents offered any SMS-program will report heights and weights resulting in *reduced* BMI (as measured by both mean BMI and above the 85% cup-point) assessed at, either 6m, 18m, or at both points in time by a joint test.

For each of the two endpoints we proceed to estimate

- effect of adding friend,
- effect of adding mother, and
- effect of tailoring

among only those who were in any of the SMS intervention arms.

In general, for hypothesis testing a p-value < 0.05 (two-tailed) will be considered statistically significant.

Sequential hierarchical testing with closed testing procedure will be applied. The overall effect of SMS will be tested as a confirmatory hypothesis. If an effect is found we then proceed to test differential effects determined by mother, friend and tailoring. Because this is a multi-layered 2x2x2 independent factorial trial, we do not believe we need to adjust for multiple comparisons between the trial arms when analyzing the overall effect of each factorial randomization. If no effect of the overall SMS is found we will still estimate possible differential effect, but regard these analyses as exploratory. Following this approach keeps the overall type I error at a 5% level for each outcome.

4. Catalogue of planned analyses addressing various additional questions

Assessing effects on SSB intake

Intake of SSB reported in adolescents offered the full SMS-program v. in adolescents offered no SMS-program (fully null group without any SMS or active education), assessed at, either 6m (FU1), 18m (FU2), or at both points in time (FU1 and FU2) by a joint test.

Background: High intake of Sugar Sweetened Beverages (SSB) is prevalent, and there is strong evidence that it has negative health effects; it is, therefore, an important parameter to assess on its own.

It should be noted that SSB has some distinct advantages as compared to the other available dietary parameters, F&V and Fish. The advantages include that SSB comes in discrete units and may therefore be relatively easier for the respondent to memorize and report on, as compared to F&V and Fish. Also, a large proportion of the adolescents may have been able to purchase SSB on their own and will therefore be more in control over their use of SSB, as compared intake of F&V and Fish which are foods that are more likely to be purchased at family level.

Assessing effects on F&V intake

Intake of F&V in adolescents offered the full SMS-program v. in adolescents offered no SMS-program (fully null group without any SMS or active education), assessed at, either 6m (FU1), 18m (FU2), or at both points in time (FU1 and FU2) by a joint test.

Background: As was the case for high SSB intake, low F&V intake is *per se* an important public health problem.

Assessing effects on Fish intake

Intake of F&V in adolescents offered the full SMS-program v. in adolescents offered no SMS-program (fully null group without any SMS or active education), assessed at, either 6m (FU1), 18m (FU2), or at both points in time (FU1 and FU2) by a joint test.

Background: As was the case for high SSB intake, low Fish intake is *per se* an important public health problem.

Assessing effect of tailoring v. full-script

Effect on SSB intake assessed among those randomized to either 'tailored SSB' or 'full' script (stratum 2)

Effect on F&V intake assessed among those randomized to either 'tailored F&V' or 'full' script (stratum 3)

Effect on Fish intake assessed among those randomized to either 'tailored Fish' or 'full' script (strata 1&4)

Background: For those who have a problem with a specific factor (be it high SSB, low F&V, or low Fish) that we want to influence, it would seem more rational to use a SMS-messaging program tailored specifically to influence that factor. The question is if such program will be as efficient (or even more efficient) at

influencing that factor, as compared to a longer SMS-messaging program that addresses a cluster of dietary factors, and thus also other issues than the specific factor.

Comparing effects in specific sub-groups

Comparing effects in boys v. girls

Background: Testing for gender effects is relevant, e.g., to answer the question: are girls more receptive to dietary advice?

Comparing effects across the three age groups

Background: Testing for age effects is relevant, e.g., to answer the questions: are more mature adolescents more receptive – or more resistant – to dietary advice?

Assessing effects in specific sub-groups of particular interest

Effects maybe enhanced in specific sub-groups, e.g.

- Sub-groups who at baseline are high in SSB; low in F&V, low in Fish, or have low score in HEI.

Background: Favorable changes in dietary habits maybe easier to detect in these sub-groups.

- Adolescents from resourceful families (according to education/incomes/residence).

Background: These may be better at taking in advice to improve their lifestyle.

- Adolescents who are overweight at baseline.

Background: These may be better/worse? at taking in advice to improve their lifestyle.

- Adolescents who score high in quiz test.

Background: These may be better at understanding the messages about adopting a healthier life style.

Interaction effect models

- of whether the combination of both the participation of a **friend** and the **mother** together during the adolescent's program has interactive effects (aka effect modification) in enhancing or attenuating the effectiveness on specified outcomes
- of whether the combination of both the participation of a **friend** and **tailored** SMS together during the adolescent's program has interactive effects (aka effect modification) in enhancing or attenuating the effectiveness on specified outcomes

- of whether the combination of both the participation of the **mother** and **tailored** SMS together during the adolescent's program has interactive effects (aka effect modification) in enhancing or attenuating the effectiveness on specified outcomes.

Identifying dietary factors with impact on overweight propensity / BMI z-score

If an effect/effects on overweight propensity / BMI z-score can be detected, a program of exploratory analyses will be developed (not further specified here) to examine if any such effect is mediated by – and can be attributed to – changes in specific dietary habits.
