# Supplementary material for: An SMS chatbot digital educational program to increase healthy eating behaviors in adolescence: A multifactorial randomized controlled trial among 7,890 participants in the Danish National Birth Cohort
Source: PLoS Med. 2024 Jun 14;21(6):e1004383. doi: 10.1371/journal.pmed.1004383 (PMC11178212; doi:10.1371/journal.pmed.1004383)
Supplement: S3 Text — (PDF) [file pmed.1004383.s003.pdf]

Project description

Project Title

Texts 4 Healthy Teens – a digital social network messaging educational program for dietary and lifestyle behavior modification among adolescents

Abstract

This proposal focuses on the delivery of a social-network based digital health educational program for educating and thereby changing dietary and lifestyle behaviors among Danish adolescents aged 14-16. Leveraging social network groups and harnessing the power of social network peer influence, the overall goal of this program is 3-fold: 1) examine the impact effectiveness of the program, 2) examine potential mechanisms underlying a possible impact effectiveness of the program in influencing social propagation and contagion of positive dietary/lifestyle habits, and 3) consider wider dissemination of the program to broader populations provided that it is found to change habits in beneficial directions.

This novel project will for the first time combine unique research methodologies and resources: a) utilize a state-of-the-art food frequency questionnaire (FFQ), b) a novel Epidemic Health SMS social network-based digital health messaging and feedback platform, c) apply a new multilevel methodology for social network analysis, d) decipher using advanced time-dependent causal analysis of social network behavior, e) leverage the power of random selection for instrumental variable causal analysis of social network phenomena, and f) use the Danish National Birth Cohort as a frame from which to identify adolescents who will be invited and offered to receive digital messaging programs with varying levels of intensity.

Apart from the high scientific value of this study, the digital messaging program most importantly target Danish adolescents with the aim of positively and contagiously modifying their lifestyle and health behaviors. If successful, the digital messaging program will not only improve their immediate health condition, but if scaled up, could shape the health of larger groups of the Danish adolescent population, and beyond.

Introduction

It is widely known that text messages are the cause of numerous injuries each year, as evidenced by ever-increasing reports of distracted pedestrians and drivers causing accidents. In this proposal we wish to use text messaging for a positive purpose: to help construct an inexpensive and scalable way of promoting health by positively shaping the behaviors of impressionable young people. We propose to examine the impact of implicating the social network of the youth in the health promotion program, and to do so using text message technology within the framework of the Danish National Birth Cohort.

Why Social Networks?

Recent sociological, anthropological, and epidemiological literature points to the power and potential of human social network interactions as a key to effective and sustained behavioral interventions and lifestyle modification for the prevention and management of chronic diseases. It

is well-established that through small group interactions, individuals influence other individuals and groups using preexisting organic social networks via a process termed ‘social induction.’ From smoking, to alcohol use, to drug use, to obesity, the emerging evidence indicates that social network phenomena are major drivers of lifestyle patterns and risk factors that contribute to obesity and metabolic risks(1).

However, the majority of these efforts have been aimed at understanding how lifestyle behaviors and risk factors naturally flow through existing social networks to lead to *unhealthy* outcomes. To date, research in this area, notably the flagship studies of Christakis and Fowler, has been observational in nature and have not addressed the potentially transformative corollary to this phenomenon, *i.e.*, that social networks can be engineered to spread *healthy* lifestyle behaviors to improve public health. Recently, Ding and Zoughbie (2013) used a randomized controlled trial design to address this gap by showing how social networks in the USA can be harnessed to induce significant and sustained weight loss, blood pressure, and waist circumference improvements(2). Additionally, systematic reviews of obesity intervention studies in school aged (3) children indicate that interventions that comprehensively educate children in nutrition and lifestyle changes like physical activity, and which involve social support elements, have been shown to be effective for obesity prevention.

Yet as evidenced by the paucity of data, systematic studies of social network interactions in the context of SMS health promotion, health propagation, and the specific social instruments that can be utilized to enhance healthy behavior modifications among adolescent youths, have simply not been conducted. Although there have been lifestyle programs involving social support and group-based interventions in Western populations(2;4), no prior programs have directly harnessed and leveraged existing SMS-based adolescent social networks for the prevention of type 2 diabetes, obesity, CVD, and other chronic conditions. Moreover, no social network analysis from settings with randomized interventions has ever conducted such a study in the context of a rich longitudinal data set such as the expansive Danish National Birth Cohort. Such a study could offer powerfully generalizable conclusions about the origins, trajectories, and prevention of major disease epidemics.

Why Social Media?

There is also a growing body of evidence demonstrating people’s interest in using and leveraging social media for health related education. In a major survey by a consulting firm, when asked how people have used social media for health-related purposes (5).

- 42% of respondents said they have used social media to look up consumer reviews of health treatments or physicians;
- 30% said they have supported a health cause through social media;
- 25% said they have shared their own health experiences on social media Web sites; and
- 20% said they have joined a health forum or online health community.

Furthermore, in the survey, social media have been found to influence people’s health decisions:

- 45% of respondents said health information obtained through social media sites would cause them to seek a second opinion;
- 41% said social media sites would influence their choice of a specific physician, hospital, or medical facility;
- More than 40% said health information on social media sites would affect how they manage a chronic condition or approach diet and exercise routines; and

- 34% said social media Web sites would affect their decision to take certain medications.

Moreover, there is a movement of parents to use mobile phones for their children's health. A study showed (6) that 55% of parents use their mobile device to improve their child's education, and 51% feel mobiles educational media have made their children more successful in school.

Why SMS?

According to a 2010 Pew survey, 75% of adolescents in the developed world have mobile phones, an increase of 45% since 2006, (7;8); additionally, the percent of individuals using mobile phones to access health information almost doubled between 2010 and 2012 (7). However, only 27% of teens in the survey reported using their mobile devices to access internet-powered mobile applications, Facebook, Twitter, and other forms of social media. The #1 most prevalent form of adolescent digital communication, therefore, is SMS messaging, which possesses unique properties of instantaneous information exchange, along with a near 100% message read rate. Indeed, about 95-98% of text messages are read within the first minute of receipt, and amongst adolescent teens, a SMS platform is the quickest and most reliable way to connect with large populations. A recent study by Nielson's also showed that adolescents on average exchange 6 text messages per hour while they were awake (9). And finally, as a comparison of mobile communication mediums, 66% of surveyed mobile users indicated that text messaging improve their social relationships, followed by picture/video messaging (35%), social networking sites (31%), email (25%) and video messaging (9%) — indicating the superior communication of SMS messages.

Examples of past successful SMS health programs

SMS messages remain the focal point of teen mobile use and SMS is therefore a prime medium for having conversations and engaging with teenagers and their friends. Indeed, SMS is much more successful for report-backs and engagement than email or web. It has been reported recently by DoSomething.org (a US non-profit specializing in new media for teenagers and young adults under 25) that their SMS opt-out rates are consistently low (under 1-percent) with response rates frequently reaching as high as 46 percent among adolescent teenagers. SMS can be engaged as a highly effective push (information dissemination) and a pull (data collection) mechanism for engaging teenagers, creating a more intimate communication channel for adolescents. The potential of using an SMS platform for social good has not gone unnoticed by other organizations. For example, *Text4baby*, a free mobile health information service that supplies pregnant women and new moms with maternal and newborn health information via text messages, is one excellent example of the benefits gained from using an SMS platform as a means of health promotion. A research team in San Diego suggested that 75.4 percent of mothers were kept informed and thus up to date with health procedures such as remembering doctor appointments, getting necessary immunizations, and learning about warning signs.

Despite the emergence of SMS platforms for health, a 2012 systematic review in the *Journal of Medical Internet Research* revealed a 'dearth of peer-reviewed studies' that rigorously examine the socio-economic and behavioral aspects of SMS messaging (4). To the best of our knowledge, no peer-review studies exist that rigorously evaluate the propagative potential of SMS messages within a social network framework. In light of the public health possibilities, we believe the Epidemic Health SMS platform—which fuses cutting edge SMS communication with social network epidemiology—when applied to the DNBC will aid in the discovery of causal mechanisms for the adoption and propagation of healthy behaviors amongst adolescents.

Why Age 14 Adolescents, Why DNBC?

Dietary intake and physical activity in youth may not only influence their growth and development (1), but also their risk of cardiovascular diseases, cancer, and other chronic diseases in later life (10;11). Several studies from industrialized countries suggest that a large proportion of youth have dietary and physical activity habits which in general terms can be described as inadequate. These habits are characterized by low intake of fruit, vegetables, and cereal products; a high intake of fast-food, sweets, and beverages high in sugar, and a sedentary lifestyle (12-16). These trends have been supported by Danish publications, including “Danish dietary habits”, published by the Danish National Food Institute (17).

The Danish National Birth Cohort (DNBC) was established in 1996-2003 as a research registry in the Danish Health and Medicines Authority (Sundhedsstyrelsen) (18;19) where 101,045 women gave consent to enroll in the study early in their pregnancy. Using questionnaires, telephone interviews, and registries it has been possible to gather extensive information on the women and their children. Thus, in several interviews during and after the pregnancy (in gestational week 16 and 30 and at 6 and 18 months after birth), detailed information was collected on health and lifestyle factors, use of medication together with social- and profession-related factors. There is also available detailed information on dietary intake during pregnancy from nearly 70,000 women, which was assessed using a questionnaire in gestational week 25 (19). Furthermore, maternal blood samples were collected during their first and second visit to their doctor’s office; in gestational week 8 to 10; and in gestational week 24. Blood from the umbilical cord was collected at birth. Follow-up, including the children, is being undertaken regularly. From 2005-2010 mothers participated in a 7-year follow-up; and in an ongoing follow-up (2010-2014) the children participate in an online self-administered questionnaire to which they are invited at the age of 11. Several measures have been initiated to increase the participants’ link to the study, for instance sending out newsletters, updating the study webpage, and the interactive webpage Club11 (www.club11.dk).

At 14 years all children in the DNBC cohort will, for the first time, be asked to complete a questionnaire on diet and physical activity. During the years 2014 and 2015, 45,000 children in DNBC will turn 14; this will provide for a unique sampling frame and opportunity for testing out methods for improving lifestyles during the years of adolescence.

The Digital Messaging Programs

This study will test and utilize the Epidemic Health SMS Program to run what will correspond to a 4-arm randomized controlled trial so that social mechanisms can be identified and evaluated with the intention of ultimately amplifying them so as to induce the maximum positive behavior change amongst adolescent youths. The Epidemic Health Model focuses on 1) increasing the quantity of minutes engaged in physical activity, 2) decreasing overconsumption of food, 3) improving the overall nutritional quality of meals consumed, and 4) tobacco avoidance.

There are 4 key components to the full SMS Epidemic Health Curriculum:

1) **Socially Communicated Preventive Health Education**
   a) Nutrition education and compliance feedback, which could include
      i) Reduction of sugar sweetened beverages (Goal: 0 servings per day)
      ii) Reduction of fast food restaurant consumption (Goal: 0 servings per day)
      iii) Increase fruits and vegetable consumption (Goal: 5 or more servings per day)
   b) Physical Activity education and compliance feedback, which could include

   i) Increase in vigorous aerobic activity (Goal: 30 minutes per day)

c) Smoking
   i) If smoker, reduction of smoking frequency (Goal: to <1/month or 0)
   ii) If non-smoker, prevention of initiation (Goal: stay non-smoker)

2) Social Network

a) Recruitment of friends and family members of their social network to join the program with them as secondary program members

b) Assessment of social network ties via Social Network Matrix Analysis.

3) Individual Self-Reporting of Body Size Measures and Adherence to Health Education Goals

a) Body size measure status report

b) Adherence to health education goals
   i) Adherence to SSB goals, fast food goals, fruits and vegetable goals, exercise goals, and smoking cessation / non-smoking goals
   ii) General happiness and other mental health ratings

4) Social Media Reporting, Support, and Peer Comparison

a) Social media newsfeed of peer-updates of social network health status progress
   i) Newsfeed provides updates of social network friends' body size measure status
   ii) Newsfeed provides updates of social network friends' nutrition/exercise/smoking status
   iii) Encouraging health messages are sent to users

48 Month Schedule of Data Collections and Analysis Activities (will be be revised)

Schedule of activities	2014	2014	2015	2015	2016	2016	2017	2017	2018
Ethical Approval									
Advisory Board Formation									
Program Translation and Piloting									
Dummy Trial Implementation and Feedback									
Program Revision									
1st FFQ and weight and height at 14y – for all 14y old in DNBC									
Identifying 6000 eligible 14y olds									
Random selection, offering participation in programs A-C									
2nd FFQ and weight and height to all in DNBC									
Analysis, Publication, and Scale-Up Planning									

Selection of Groups Offered Participation in the Digital Messaging Programs

The around 45,000 children who turn 14y during two years in DNBC will all be invited to complete a FFQ, and again approximately one year later (for description of methods, see later). We are planning to identify a subgroup of ~6,000 of these, from whom three A-Cgroups will randomly selected and offered participation in various levels of the digital messaging program, whereas the remaining children, making up Arm D, will not be offered participation in a digital messaging program; according to current plans, the ratio between arms A:B:D:C will be 1500:1500:1500:1500, but this may change depending on the conclusions from the pilot phase (an alternative ratio considered is 1000:1000:1000:3000). The subgroup will comprise children who in their FFQ completed at 14 report lifestyle characteristics that can be made subject to possible improvement by the digital messaging programs; these characteristics could include consumption of sugary beverages above a certain, high level, or rare performance of leisure physical activity, etc.; the criteria for selection will be decided upon careful consideration during the initial phase of the project. The full program (arm A)will be 3 months in duration followed by health reminder follow-ups and further data collection.

Translation and Cultural Sensitivity

The investigators have considerable experience in social epidemiological and anthropological research methods and previously designed three major social network trials in the United States, the Kingdom of Jordan, and Qatar. Style and wording of messaging will be tested during focus groups with Danish young people, and most importantly, will be piloted during the dummy trial to ensure that information is appropriate and understandable to Danish youth.

Functionality of the SMS Based Platform in a Danish Context; Online Educational Portal

Each person who joins the trial will need to text message the word JOIN (or a Danish translation) to a specific phone number. They will then automatically join the study and will receive text messages from the computer that they can reply to, and which will generate automatic responses. Person eligible for the trial may recruit a group of persons; for that they will have to text in a special group ID which we give each person, and they are automatically grouped together.

Once these steps are taken, each person who is enrolled in the program receives periodic information (PUSH), and is asked questions (PULL). Running the study requires ongoing attention and extensive monitoring and data management.

All SMS messages are sent through an international telecommunications carrier. The platform will work in Denmark. Distinct features of the platform include that it enables 1) to run digital health messaging programs in groups defined by random selection, and 2) to create groups and networks; we are not aware of the existence of any similar systems.

An additional online portal is planned to be created for this project to provide educational resources and other information for participants in the project. Educational information on healthy diet and lifestyle will be presented in a way that has appeal to the young people; it may include competitions about suggesting (the most funny, exciting, exotic etc.) healthy recipes, where they can win iPADs or iTunes downloads. This will be developed in collaboration with the Nutrition Source at Harvard School of Public Health http://www.hsph.harvard.edu/nutritionsource/ .

Digital Messaging Programs

As shown in Figure 1, each arm will consist of:

- **Arm A** receives:
  - Recruitment of friends and family members from their social networks to join the program with them as secondary program members, plus assessment of social network ties via Social Network Matrix Survey.
  - The full Epidemic Health Educational Curriculum with adolescent-tailored programming about healthy diet, exercise, and tobacco avoidance – plus encouragement to share healthy activities with friendship group.
  - Each member will report back information, via SMS, about their weight, height, basic diet, and basic lifestyle habits.
  - Social network news feeds and social support features: each member of Arm A, as well as their social network friends, will receive social news feeds of the diet and lifestyle status and progress of the other members of their social network, as well as ability to get social support feedback from friends.

- **Arm B** receives:
  - Recruitment of friends and family members of their social network to join the program with them as secondary program members, plus assessment of social network ties via Social Network Matrix Survey.
  - Partial Epidemic Health Educational Curriculum with information about healthy diet and lifestyle.
  - However, there will be no social network component to the program – i.e. no social news feed status updates of friends' health and activities, and no social support feedback from friends on the platform.
  - Each member will report back information, via SMS, about their weight, height, basic diet, and basic lifestyle habits.

- **Arm C** receives:
  - No recruitment of friends and family members.
  - No Epidemic Health Educational Curriculum program and no social sharing components.
  - No social network newsfeed status (because no friends recruited), and no social network support feedback on platform
  - Each member will simply report back information, via SMS, about their weight, height, basic diet, and basic lifestyle habits.

- **Arm D** will not be offered to participate in a digital message program, but like all arms, will receive the dietary FFQ and other assessment protocols of DNBC participants.

Study Objectives

Objective 1: Does the SMS based-messaging program work? To evaluate the efficacy of the full digital health messaging program(education + social network support + SMS data collection) compared to a group receiving a modified behavioral health program without social network interactions (education + SMS data collection) compared to SMS data collection only and a non-intervention control group in improving lifestyle factors, we shall:

a) Complete what will correspond to **a four-arm parallel** study of impact of participating in programs of various intensities, where the full program implies participation in a 3 month digital health messaging interaction and follow-ups every three months.
b) Assess the lifestyle behavioral change effects (*e.g.* changes in diet, physical activity, smoking patterns) of arm A against arms B, C, and D:
    a. Comparing A vs. B: Estimate effects of social network peer-newsfeed update and social network peer support system of SMS platform
    b. Comparing B vs. C: Estimate social network sharing and monitoring effects
    c. Comparing C vs. D: Estimate basic Epidemic Health SMS Educational Curriculum effects
c) Assess the self-reported anthropometric changes (*e.g.* weight, height) of arm A against arms B, C, and D (and B vs. C; C vs. D, per above).
d) Evaluate the sustainability and long-term advantage of the digital messaging program in maintaining healthy lifestyle behaviors up to 2 years after when compared to control B (lacking social networking), C (lacking education and social networking), and D (lacking social networking, education, and SMS health monitoring).
e) Evaluate the impact of participants' extended social ties (collected with our social-network-matrix) on the effectiveness and long-term impact on affecting healthy lifestyle behaviors, knowledge and skills on, e.g., diabetes, and diet and physical activity.

Objective 2: Why does it work? To define the pathways through which the social network behavioral health program worked by partitioning the different social network processes of **direct causal induction** (*i.e.* change catalyzed by a social network) from **homophilly** (*i.e.* where social networks are formed based on similar health behaviors), and **confounding** (*i.e.* correlation with exogenous shared variable). Notably, direct causal induction effects will be partitioned from homophilly associated effects on health behaviors via various methods isolating the social network induction.

a. Using multilevel repeated measures analysis, we shall partition the induction effects from homophilly by evaluating the magnitude of longitudinal behavioral changes in the group receiving the full program by using multilevel repeated measures analysis and discounting for natural background correlation among known friends/relatives in controls.
b. Utilizing a rich collection of longitudinal data and temporal changes in behavioral factors, estimate the differences in the trajectories of change between partners overtime to determine the presence of any between-person (*i.e.* social cross-propagating) cascading of effects within social clusters and between social clusters, and the determinants of such social clusters with greatest and least social propagation.
c. To disaggregate social induction *vs.* homophilly of shared familial factors, we shall investigate whether social clusters comprised of first-degree biological and co-habitation family members exhibit stronger social network effects on lifestyle (diet and physical activity).

Analysis Plan

The first analysis (**Objectives 1a**) in this proposed work will examine the baseline characteristics of the study subjects in each treatment arm within the two studies with respect to demographic variables lifestyle factors, including diet, smoking, and exercise. Baseline social network clustering will be assessed to establish background social correlations.

For direct analysis of causal effects on continuous variables of lifestyle change from baseline up to 18 months, we will use longitudinal multilevel repeated measures analysis using unstructured (maximally conservative) covariance matrices. The key time-by-treatment interaction terms (between arms and follow-up time) shall be included in the models to test for differences between arms and comparison group(s) over time in lifestyle changes. Time shall be conservatively modeled using indicators, to allow for maximal data-driven flexibility in temporal patterns.

In further comprehensive assessments, repeated measure multilevel modeling will specifically leverage the hierarchal nested structure of the multiple social aggregation levels. Specifically, individuals within social clusters, within multiple social clusters, within geographical areas, and within temporal-cohorts, will be used to quantify degree of social clustering of changes in lifestyle outcomes. First, these models will be analyzed to measure the mean difference in lifestyle factors at the 3, 6, 9, 12 and 18 -month time points adjusting for confounders. Second, the careful modeling of the multi-levels will not only improve precision, but more importantly partition the degree and structure of variances of each program—quantifying the magnitude of clustering in changes of lifestyle outcomes within each cohort cycle, neighborhood, social network group, and individual levels.

Non-linear quadratic coefficients and splines shall assess for non-linearity of lifestyle changes and trajectories over time, particularly evaluating the apex of maximal behavioral efficacy. To account for missing data and loss-to-follow-up, we shall use various, imputation methods, from conservative methods (*i.e.* baseline value carried forward), to sequential regression multiple imputation strategy (SRMI), and potentially including inverse probability weighting of missingness (akin to marginal structural models [MSM]) in situations involving time-dependent dropout and non-differential missingness.

For evaluations of binary behavioral and knowledge acquisition outcomes, Cox Proportional Hazards models will be used to estimate the hazards ratio, allowing for variable lengths of follow-up of binary time-to-event outcomes. Repeated measures logistic mixed and Poisson mixed models will be used to assess change in prevalence risk ratios of lifestyle outcomes at end of follow-up. Analyses will be adjusted for any baseline factors that are randomization imbalanced. All tests are 2-sided with $\alpha=0.05$.

To address **Objectives 1d,** the multi-level models will be re-run incorporating aspects of the social network. First, our novel and comprehensive ***SMS social network*** **system** will enable us to map social network interactions; within social levels will be used to characterize the a) quantity, b) qualitative frequency, and c) qualitative strength of social networks, as well as the degree of social connectedness within and between social clusters and other layers. Second, these characteristics of the networks will be included in all previous models as confounders as well as effect modifiers to examine the impact of social network characteristics on the effectiveness and sustainability of the social network behavioral health program.

The questions under **Objectives 2a, b, and c** will be addressed during Phase 3 of the study. This Phase will focus on analyzing the social network's effects in differentiating between three network modalities—1) causal induction, and non-causal clustering of 2) homophilly and 3) confounding. Further for causation, we also shall determine the extent of long-term temporal cross-

propagation between individuals within a social cluster, and between clusters via our unique *Social Network Matri*x, which enables us to track social relationships. Direct causal induction effects will be partitioned from non-causal factor for outcome trajectories by relying on various analytical methods to isolate and quantify the role of social network induction.

Social Network Effect Size: Using multilevel repeated measures mixed models, we shall partition induction effects by evaluating the magnitude of longitudinal lifestyle change trajectory in the group in question while correcting for both natural background magnitude and background correlation (covariance) among existing friends/family clusters in the control groups. Notably, this allows us to partition out the causal induction effects from the natural homophilly and confounding of shared genetic and social factors. Furthermore, for this, we also innovated a new metric of social network propagation attributable to a social level: a *Ratio of Intraclass Correlations* (ICCR), a ratio to quantify relative magnitude of social propagation effect between groups, where ICCR=1 represent equal effects of social network clustering, while ICCR>1 or <1 represent greater/lower acceleration of social network propagation. Thus, ICCR holds promise for measuring the impact of relative induction of social network effects. Finally, to study the causal effects of social network lifestyle factors, the unique causal methodological approach of instrumental variable (IV) analysis can be applied for our analysis--which has not been utilized before in such a setting because no prior study has ever had a randomized social network program for an instrument. Notably, our IV causal analysis can leverage our trials' use of random selection (as the unconfounded instruments for social network behavior) to elucidate causal effects for social networks and behavior.

Degree of Propagation. In addition, the rich longitudinal data collection of temporal sequence and changes in behavioral factors will allow us to estimate in a prospective temporal fashion the causally induced changes in a given parameter between time intervals. First, the person-time intervals between follow-ups will be analyzed as separate induction timeframe windows, in which the changes observed in the T=0 person-time window in the node individual of a cluster, is used to predict the outcome in the subsequent T=1 person-time interval of other partners inside and outside of the social cluster, all while adjusting for concurrent time-varying changes in other covariates (via inverse probability weights, now available in STATA 12). Secondly, we shall assess whether second-order changes at T=1 in other friends and family members may cyclically propagate back to inducing changes in the primary node individual and change in other MC groups in a subsequent tertiary T=2 person-time interval--and so on, until we isolate the degree of between-person propagation and between-cluster propagation effects. For this, the help of special time-dependent confounding models, such as Marginal Structural Models using inverse probability weighting, will be used to causally analyze and isolate such time-depending, temporal network relationships.

Effects of Familial/Biological Ties. Finally, we shall seek to ascertain whether the network effects differ by the degree of familial interrelationships (**Objectives 1e**) within a social cluster. Each social cluster will be examined along multiple factors: 1) whether intra-social cluster group members are related by friendship ties or familial tie (or proportion familial if mixed), 2) whether social cluster group members of familial bonds are biological or non-biological (*e.g.* spouses), and 3) whether biological relationships are parent-child, siblings, cousins, or more distant. Using the repeated measures analysis approach described above, these three intra-social cluster group familial network factors will be investigated with changes in behavioral trajectories.

Assessment of Dietary Intake and Physical Activity at 14 Years

Around 45,000 children in DNBC will turn 14 during 2014 and 2015, during which years selection into the study arms A-D will take place (see section above on 'Selection of Groups Offered

Participation in the Digital Messaging Programs'). In the following we justify the method we use for assessing dietary intake at 14 years and describe how it was developed. The majority of 14 year olds have sufficient language skills and reading abilities to complete a self-administered questionnaire. However, some conditions can be expected to have a negative influence on how accurate the children in this age group report dietary intake and physical activity: unstructured eating habits and problems in relation to self-perception of weight and body image. These conditions may also affect the children motivation to complete an accurate report (20-22). Therefore the choice of assessment method is a critical point (23;24). Food frequency questionnaires (FFQs) are both a practical and economically achievable method to assess dietary intake in youth (25). FFQs have previously been used with success in large epidemiological studies in adolescents. Experiences from these studies show that FFQs provide data with acceptable validity and reliability which can be used to rank adolescent according to magnitude of intake of foods and nutrients (26-28). Furthermore, experience show that the main limitation of FFQs is measurement errors because of limited food databases, lack of accuracy in reported frequency of consumption of foods, and portion sizes. For the latter, the complex cognitive process necessary in order to report accurate portion sizes is considered to be a difficult task for adolescents because their portion sizes often vary substantially between meals(29). Compared to adults, youth in general pay less attention to how large portions they consume (12). However, it has been shown that it is possible to improve skills to better estimate portions sizes by training and by using age-specific graphic aids (30;31). Nonetheless, it seems that including portions sizes does not significantly add to the results compared to the problems it generates with respect to increased respondent burden and increased risk of missing answers in the questionnaire. The contribution to increase validity of an FFQ from including questions on portions sizes in an FFQ (a quantitative FFQ) is estimated to be limited besides the information already being collected by the frequency questions (32;33). Therefore focus has been on developing questionnaire without quantification (that is studies where individual portions sizes are not being recorded but instead an assumption of standard portion sizes is being applied) as the 'the method of choice' to rank individuals, making a relative provision, meaning relative in relation to other respondents, with respect to their consumption of specific foods, nutrients, and dietary pattern. Additionally, FFQs can assess information on dietary eating patterns typical of the respondent (34;35). Several FFQs have been developed for adolescents in other countries (25;36;37); however, it has been necessary to modify our FFQ specifically to Danish adolescents (25;(38)).

The questionnaire has been developed through several phases which we will describe briefly: The Principal Investigator has during several visit at Department of Nutrition discussed the possibilities to develop a Danish questionnaire in cooperation with the people responsible for the American Nurses' Health Study (NHS, based at Harvard) and the Growing Up Today Study (GUTS) which is a follow-up of the offspring of the nurses (meaning the second generation of NHS). The group at Harvard developed a questionnaire for GUTS for adolescents and we have had the privilege to be able to use the software and other facilities which the GUTS FFQ is built upon. The main applicant and his colleagues received in 2010 a grant from the Danish Council for Strategic Research (DCSR) to establish the Center of Fetal Programming and this grant has made the cooperation between the applicant's research team and researchers at the nutrition group from the MoBa study in Norway, the Danish National Food Institute, and the GUTS study, possible. The American questionnaire has through this collaborative process been translated and modified to Danish culture after a pre-test in Danish schools. This has also resulted in an update of the food database. Additionally, an advanced calculation program has been developed which enables us to estimate intake, not only as food frequencies but also as quantified amounts of foods and nutrients with the assumption of standard portion sizes and using the Danish food tables.

Evaluators of this protocol can view the current version of the questionnaire via the URL: http://bsmb14.limequery.com/71992/lang-da (log in as Evaluator1, Evaluator2, … , Evaluator10).

Organizing data collection on diet

All participants within the DNBC will be contacted via their mother (on occasion their father) in the week around their 14-year old birthday. All respondents (postal letter/e-mail) will receive the first invitation, which will include a link and login to the online questionnaire. In the first invitation the mother (or father) will be asked to pass on the information to the child, who again will be encouraged to fill out the questionnaire or unsubscribe from the study by contacting the study coordinator if he/she does not wish to participate. If the child does not respond to the invitation, after 2 weeks the first reminder will be sent out via e-mail or postal letter. Again 2 weeks after the first reminder a second reminder will be sent if a response is not registered. A third reminder letter will be sent after another 2 weeks by postal letter to everyone who have not yet either filled in the questionnaire or unsubscribed from the study. A similar procedure will be developed for the second FFQ which will be sent around one year later to all children.

The FFQ data collection is conducted by the DNBC Maternal Nutrition group in collaboration with the DNBC secretariat led by the DNBC project coordinator, Inger Kristine Meder. The setup and management of the IT-logistics is carried out by the data manager in the Maternal Nutrition Group.

Ethical Considerations

The investigators will seek approval that may be needed from the Scientific-Ethical Committee and the Danish Data Protection Agency (Datatilsynet) to conduct data collection. All research will be carried out in accordance with the Helsinki-declaration, and the guidelines set forth by the DNBC Management Group and Steering Committee, and will follow all rules set out for security of data. To incentivize the adolescents to fill out the questionnaire, they may be invited to participate in a draw for gift cards and movie tickets as described earlier. It will be clearly stated that the respondent will volunteer to participate and can drop from the study at any time.

The Importance and Potential of the Project

Examining how to modify dietary and physical activity habits among 14 year olds in Denmark would be of potentially great benefit to society, with important lessons that can be used for a national health program in Denmark and other countries.

More detailed analyses could contribute to clarifying what factors determine dietary and physical activity habits among adolescents, such as socio-demographic conditions of the family or life style habits of the mother, and thereby enhance our understanding of which relations play a role when the dietary and physical activity habits of the children are formed.

The rich and rare social networking data that would be obtained, combined with the longitudinal nature of the DNBC, can provide us with the opportunity to obtain a robust understanding of the origins and trajectory of major disease epidemics. It would enable us to better understand how to prevent these epidemics, especially during the years of adolescence.

If the trial component of the study clearly demonstrates an effect of implicating the social network in the digital messaging program , it will be important to be facilitate implementation of this knowledge. To this end we plan to establish and activate a Project Advisory Board comprised of members of the Danish national public health service and other public health stakeholders, as well as the PI and sponsors of the study. We suggest that the Board be established and its commission

defined as early as possible during the course of the project, and that it will convene regularly, e.g. every six months, to review progress of the project.

Reference List

(1) Evans WD, Abroms LC, Poropatich R, Nielsen PE, Wallace JL. Mobile health evaluation methods: the Text4baby case study. J Health Commun 2012;17 Suppl 1:22-9.

(2) Ding E, Prescott M, Watson K, Bui N, Makarechi L, Zoughbie D. Microclinic Social Network Lifestyle Intervention for Weight Loss and Obesity Management: A 10-Month Randomized Controlled Trial . Circulation 2013 March 26;127(12 supp.).

(3) Sobol-Goldberg S, Rabinowitz J, Gross R. School-based obesity prevention programs: A meta-analysis of randomized controlled trials. Obesity (Silver Spring) 2013 June 22.

(4) Deglise C, Suggs LS, Odermatt P. SMS for disease control in developing countries: a systematic review of mobile health applications. J Telemed Telecare 2012 July;18(5):273-81.

(5) Social Media "Likes" Healthcare: From Marketing to Social Business. 2012.
Ref Type: Online Source

(6) U.S. Cellular Survey Reveals Better Moments via Mobile Devices. 2013.
Ref Type: Online Source

(7) Pew: 19 percent of smartphone users have health apps. 2013.
Ref Type: Online Source

(8) Bernstein E. Why Texting Turns Us Back Into Teenagers . Wall Street Journal . 2011.
Ref Type: Magazine Article

(9) U.S. Teen Mobile Report Calling Yesterday, Texting Today, Using Apps Tomorrow. 2010.
Ref Type: Online Source

(10) Kavey RE, Daniels SR, Lauer RM, Atkins DL, Hayman LL, Taubert K. American Heart Association guidelines for primary prevention of atherosclerotic cardiovascular disease beginning in childhood. Circulation 2003 March 25;107(11):1562-6.

(11) Linos E, Willett WC, Cho E, Frazier L. Adolescent diet in relation to breast cancer risk among premenopausal women. Cancer Epidemiol Biomarkers Prev 2010 March;19(3):689-96.

(12) Neumark-Sztainer D, Story M, Hannan PJ, Croll J. Overweight status and eating patterns among adolescents: where do youths stand in comparison with the healthy people 2010 objectives? Am J Public Health 2002 May;92(5):844-51.

(13) Nicklas TA, Baranowski T, Cullen KW, Berenson G. Eating patterns, dietary quality and obesity. J Am Coll Nutr 2001 December;20(6):599-608.

(14) Vereecken CA, De HS, Maes L. Adolescents' food habits: results of the Health Behaviour in School-aged Children survey. Br J Nutr 2005 September;94(3):423-31.

(15) Rockell JE, Skidmore PM, Parnell WR, Wilson N. What children eat during afternoons and evenings: is it important? Public Health Nutr 2010 December 8;1-6.

(16) Diethelm K, Jankovic N, Moreno LA, Huybrechts I, De HS, De VT et al. Food intake of European adolescents in the light of different food-based dietary guidelines: results of the HELENA (Healthy Lifestyle in Europe by Nutrition in Adolescence) Study. Public Health Nutr 2012 March;15(3):386-98.

(17) Pedersen TP, Meilstrup C, Holstein BE, Rasmussen M. Fruit and vegetable intake is associated with frequency of breakfast, lunch and evening meal: cross-sectional study of 11-, 13-, and 15-year-olds. Int J Behav Nutr Phys Act 2012;9:9.

(18) Olsen J, Melbye M, Olsen SF, Sorensen TI, Aaby P, Andersen AM et al. The Danish National Birth Cohort - its background, structure and aim. Scand J Public Health 2001 December;29(4):300-7.

(19) Olsen SF, Mikkelsen TB, Knudsen VK, Orozova-Bekkevold I, Halldorsson TI, Strom M et al. Data collected on maternal dietary exposures in the Danish National Birth Cohort. Paediatr Perinat Epidemiol 2007 January;21(1):76-86.

(20) Livingstone MB, Robson PJ, Wallace JM. Issues in dietary intake assessment of children and adolescents. Br J Nutr 2004 October;92 Suppl 2:S213-S222.

(21) Livingstone MB, Robson PJ. Measurement of dietary intake in children. Proc Nutr Soc 2000 May;59(2):279-93.

(22) Thompson FE, Subar AF. Dietary Assessment Methodology. Nutrion in the Prevention and Treatment of Disease 2008.

(23) Magarey A, Watson J, Golley RK, Burrows T, Sutherland R, McNaughton SA et al. Assessing dietary intake in children and adolescents: Considerations and recommendations for obesity research. Int J Pediatr Obes 2010 September 28.

(24) Moreno LA, Kersting M, De HS, Gonzalez-Gross M, Sichert-Hellert W, Matthys C et al. How to measure dietary intake and food habits in adolescence: the European perspective. Int J Obes (Lond) 2005 September;29 Suppl 2:S66-S77.

(25) Rockett HR, Berkey CS, Colditz GA. Evaluation of dietary assessment instruments in adolescents. Curr Opin Clin Nutr Metab Care 2003 September;6(5):557-62.

(26) Berkey CS, Rockett HR, Field AE, Gillman MW, Frazier AL, Camargo CA, Jr. et al. Activity, dietary intake, and weight changes in a longitudinal study of preadolescent and adolescent boys and girls. Pediatrics 2000 April;105(4):E56.

(27) Frank GC, Nicklas TA, Webber LS, Major C, Miller JF, Berenson GS. A food frequency questionnaire for adolescents: defining eating patterns. J Am Diet Assoc 1992 March;92(3):313-8.

(28) Rockett HR, Breitenbach M, Frazier AL, Witschi J, Wolf AM, Field AE et al. Validation of a youth/adolescent food frequency questionnaire. Prev Med 1997;26:808-16.

(29) Watanabe M, Yamaoka K, Yokotsuka M, Adachi M, Tango T. Validity and reproducibility of the FFQ (FFQW82) for dietary assessment in female adolescents. Public Health Nutr 2011 February;14(2):297-305.

(30) Lillegaard IT, Overby NC, Andersen LF. Can children and adolescents use photographs of food to estimate portion sizes? Eur J Clin Nutr 2005 April;59(4):611-7.

(31) Foster E, Matthews JN, Nelson M, Harris JM, Mathers JC, Adamson AJ. Accuracy of estimates of food portion size using food photographs--the importance of using age-appropriate tools. Public Health Nutr 2006 June;9(4):509-14.

(32) Buzzard IM, Stanton CA, Figueiredo M, Fries EA, Nicholson R, Hogan CJ et al. Development and reproducibility of a brief food frequency questionnaire for assessing the fat, fiber, and fruit and vegetable intakes of rural adolescents. J Am Diet Assoc 2001 December;101(12):1438-46.

(33) Schlundt DG, Buchowski MS, Hargreaves MK, Hankin JH, Signorello LB, Blot WJ. Separate estimates of portion size were not essential for energy and nutrient estimation: results from the Southern Community Cohort food-frequency questionnaire pilot study. Public Health Nutr 2007 March;10(3):245-51.

(34) Lazarou C, Panagiotakos DB, Matalas AL. Foods E-KINDEX: a dietary index associated with reduced blood pressure levels among young children: the CYKIDS study. J Am Diet Assoc 2009 June;109(6):1070-5.

(35) Magarey A, Golley R, Spurrier N, Goodwin E, Ong F. Reliability and validity of the Children's Dietary Questionnaire; a new tool to measure children's dietary patterns. Int J Pediatr Obes 2009;4(4):257-65.

(36) Watson JF, Collins CE, Sibbritt DW, Dibley MJ, Garg ML. Reproducibility and comparative validity of a food frequency questionnaire for Australian children and adolescents. Int J Behav Nutr Phys Act 2009;6:62.

(37) Wong JE, Parnell W, Black KE, Skidmore PM. Reliability and relative validity of a food frequency questionnaire to assess food group intakes in New Zealand adolescents. Nutr J 2012 September 5;11(1):65.

(38) Cade J, Thompson R, Burley V, Warm D. Development, validation and utilisation of food-frequency questionnaires - a review. Public Health Nutr 2002 August;5(4):567-87.

(39) Olsen SF, Sorensen JD, Secher NJ, Hedegaard M, Henriksen TB, Hansen HS et al. Randomised controlled trial of effect of fish-oil supplementation on pregnancy duration. Lancet 1992;339:1003-7.

(40) Olsen SF, Secher NJ, Tabor A, Weber T, Walker JJ, Gluud C. Randomised clinical trials of fish oil supplementation in high risk pregnancies. Fish Oil Trials In Pregnancy (FOTIP) Team. BJOG 2000 March;107(3):382-95.

(41) Knudsen VK, Hansen HS, Osterdal ML, Mikkelsen TB, Mu H, Olsen SF. Fish oil in various doses or flax oil in pregnancy and timing of spontaneous delivery: a randomised controlled trial. BJOG 2006 May;113(5):536-43.
