# Supplementary material for: An SMS chatbot digital educational program to increase healthy eating behaviors in adolescence: A multifactorial randomized controlled trial among 7,890 participants in the Danish National Birth Cohort
Source: PLoS Med. 2024 Jun 14;21(6):e1004383. doi: 10.1371/journal.pmed.1004383 (PMC11178212; doi:10.1371/journal.pmed.1004383)
Supplement: S1 Table — (DOCX) [file pmed.1004383.s004.docx]

**Table 3 with an alternative statistical method for adjustment: CACE. Effect estimates of primary outcomes endpoints among adolescents at 6- and 18-months follow-up receiving an SMS intervention compared with non-SMS group.**

|  |  |  | **ITT analyses^a^** | | | | | | | **IPW participant-analyses^b^** | | | | | | |
| --- | --- | --- | --- | --- | --- | --- | --- | --- | --- | --- | --- | --- | --- | --- | --- | --- |
| **Effects on:** | **Effects of:** | **Follow-up** | **Estimate** | **95% CI** | |  | ***p*^c^** | ***p^d^*** | | | **Estimate** | | **95% CI** | | ***p*^c^** | ***p^d^*** |
| **Mini**  **HEI** | **AnySMS vs.** | 6 months | 0.016 | -0.011 | 0.043 |  | 0.253 | 0.172 | | | 0.027 | | -0.002 | 0.056 | 0.072 | 0.119 |
|  | **Non-SMS** | 18 months | -0.016 | -0.045 | 0.013 |  | 0.286 |  |  |  | -0.005 | | -0.036 | 0.026 | 0.755 |  |
| **BMIz-score** | **AnySMS vs. Non-SMS** | 6 months | -0.010 | -0.035 | 0.015 |  | 0.442 | 0.635 | | | -0.028 | | -0.055 | -0.001 | 0.046 | 0.111 |
|  |  | 18 months | 0.002 | -0.029 | 0.033 |  | 0.901 |  | | | -0.006 | | -0.039 | 0.027 | 0.724 |  |
|  |  |  |  |  |  |  |  | |  | | |  |  |  |  |  |

All analyses are adjusted for the full combination of sex, age group, and diet strata and the baseline value corresponding to the outcome.

a: ITT: intention-to-treat analysis comparing all adolescents randomized to (any) SMS intervention with non-SMS group.

b: IPW: inverse probability weighting analysis comparing all adolescents randomized to (any) SMS intervention with non-SMS group. Analyses excluding non-participants of the SMS intervention. Observations are weighted by their inverse probability of participation given their predictors at DNBC baseline and analyses are additionally adjusted for grouped (ten groups) propensity scores.

c: *p*-value for individual test testing effects at each follow-up time

d: *p*-value for joint test testing effects at 6 months and 18 months at the same time

DNBC: Danish National Birth Cohort, SMS: Short Messages Service, SSB: sugar-sweetened beverages, FV: fruits and vegetables, HEI: Healthy Eating Index constitutes of SSB, FV, and Fish, BMI: Body Mass Index z-score defined by the Centers for Disease Control and Prevention (CDC) standard [37,38].
