# Supplementary material for: An SMS chatbot digital educational program to increase healthy eating behaviors in adolescence: A multifactorial randomized controlled trial among 7,890 participants in the Danish National Birth Cohort
Source: PLoS Med. 2024 Jun 14;21(6):e1004383. doi: 10.1371/journal.pmed.1004383 (PMC11178212; doi:10.1371/journal.pmed.1004383)
Supplement: S1 Fig — (PPTX) [file pmed.1004383.s007.pptx]

## Slide 1
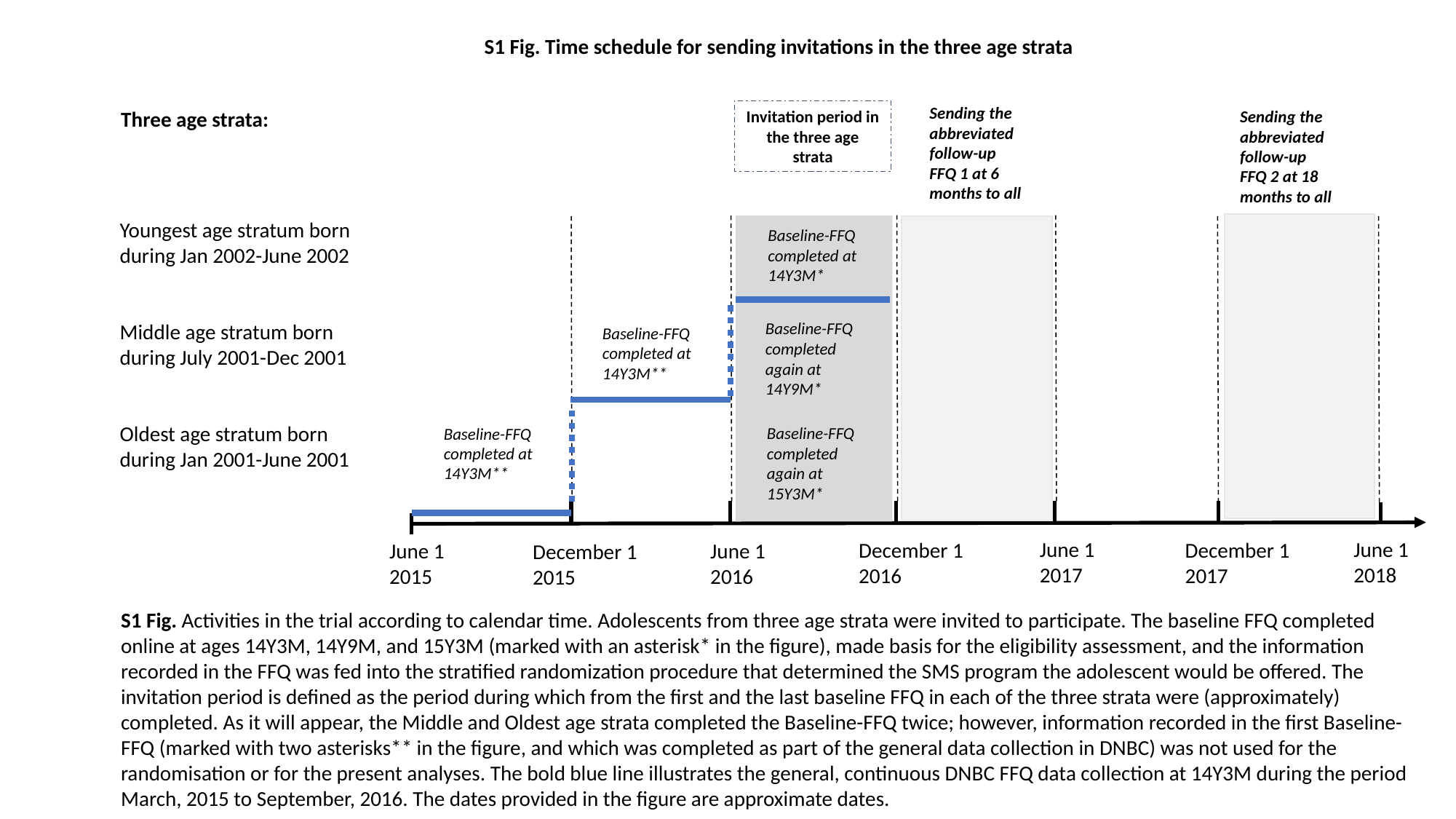

S1 Fig. Time schedule for sending invitations in the three age strata
Sending the abbreviated follow-up FFQ 1 at 6 months to all
Three age strata:
Invitation period in the three age strata
Sending the abbreviated follow-up FFQ 2 at 18 months to all
Youngest age stratum born during Jan 2002-June 2002
Middle age stratum born during July 2001-Dec 2001
Oldest age stratum born during Jan 2001-June 2001
Baseline-FFQ completed at 14Y3M*
Baseline-FFQ completed again at 14Y9M*
Baseline-FFQ completed at 14Y3M**
Baseline-FFQ completed at 14Y3M**
June 1 2015
December 1 2015
Baseline-FFQ completed again at 15Y3M*
June 1 2017
June 1 2018
December 1
2016
June 1 2016
December 1 2017
S1 Fig. Activities in the trial according to calendar time. Adolescents from three age strata were invited to participate. The baseline FFQ completed online at ages 14Y3M, 14Y9M, and 15Y3M (marked with an asterisk* in the figure), made basis for the eligibility assessment, and the information recorded in the FFQ was fed into the stratified randomization procedure that determined the SMS program the adolescent would be offered. The invitation period is defined as the period during which from the first and the last baseline FFQ in each of the three strata were (approximately) completed. As it will appear, the Middle and Oldest age strata completed the Baseline-FFQ twice; however, information recorded in the first Baseline-FFQ (marked with two asterisks** in the figure, and which was completed as part of the general data collection in DNBC) was not used for the randomisation or for the present analyses. The bold blue line illustrates the general, continuous DNBC FFQ data collection at 14Y3M during the period March, 2015 to September, 2016. The dates provided in the figure are approximate dates.
