# Supplementary figures and images for: An SMS chatbot digital educational program to increase healthy eating behaviors in adolescence: A multifactorial randomized controlled trial among 7,890 participants in the Danish National Birth Cohort
Source: PLoS Med. 2024 Jun 14;21(6):e1004383. doi: 10.1371/journal.pmed.1004383 (PMC11178212; doi:10.1371/journal.pmed.1004383)

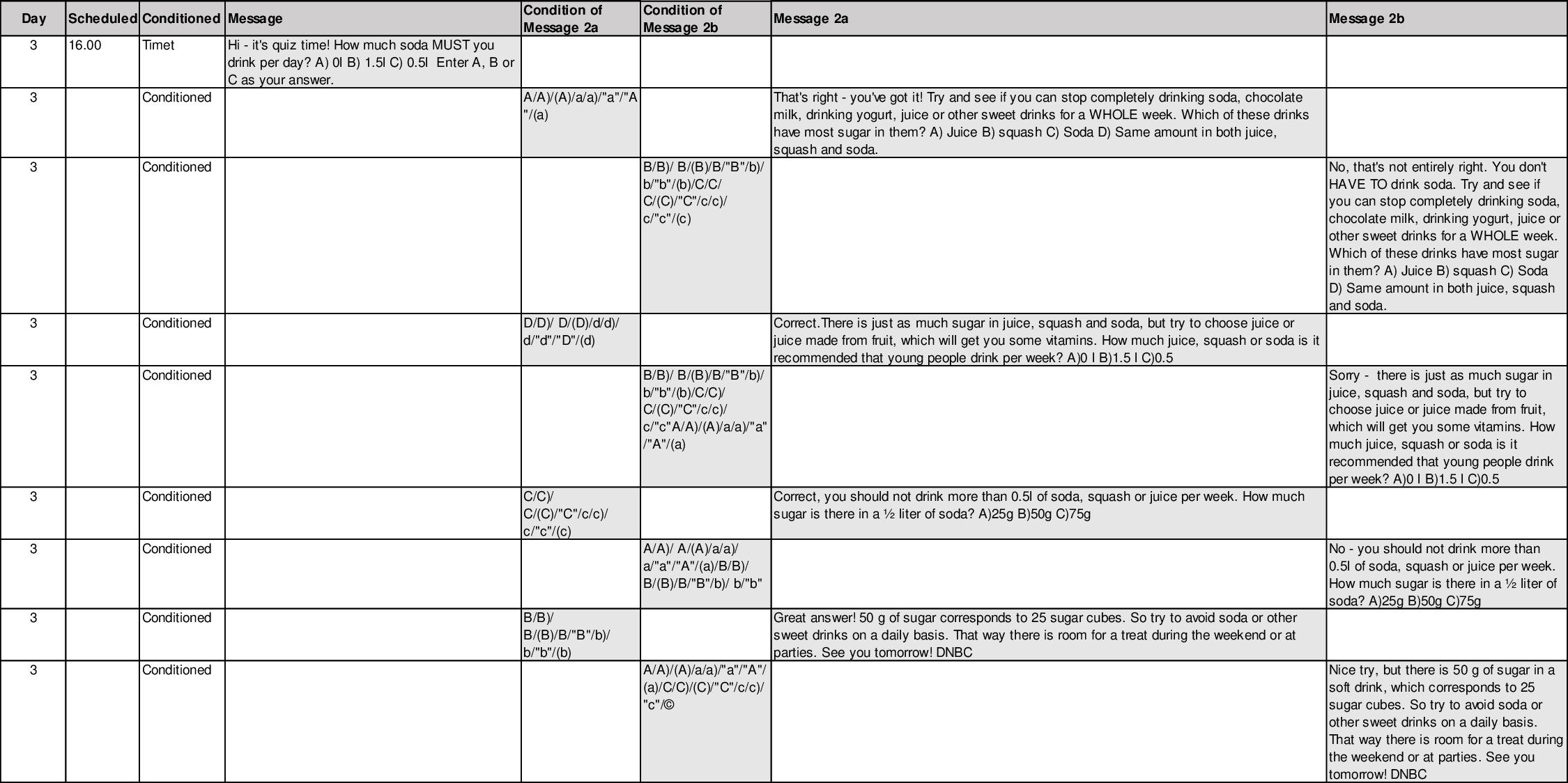

Supplement: S2 Fig — (TIF) [file pmed.1004383.s008.tif]

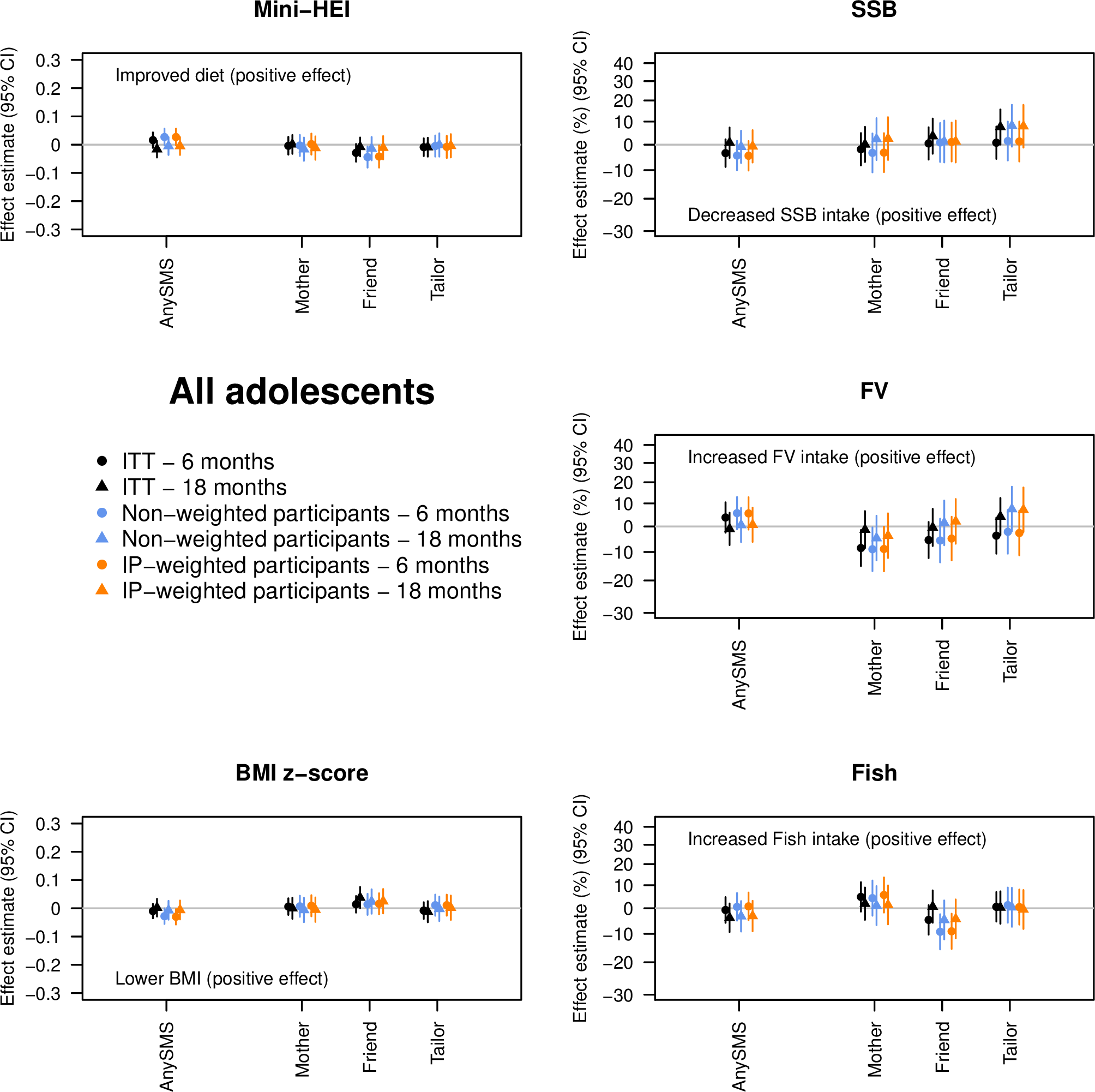

Supplement: S3 Fig — Analyses of the two main outcomes, mini-HEI and BMI z-score, are shown in the upper and lower left panel, respectively, on each page; whereas analyses of the secondary outcomes, SSB, FV and Fish z-scores, are shown in the upper, middle and lower right panel, respectively. In each panel are shown analyses of the effect estimates for the continuous outcomes performed for AnySMS compared to Non-SMS (the estimate to the left in each panel); and for each of the three additional elements, i.e. adding mother compared to not adding mother, adding friend compared to not adding friend, or tailored SMS program compared to the full SMS program, assessed within the group of AnySMS, thus excluding the Non-SMS group (the three estimates to the right in each panel, respectively). Effect sizes are estimated differences between comparison groups in means of outcomes at 6 months and 18 months follow-up. Colors indicate effects estimated from intention-to-treat (ITT)-analyses including all individuals (black) and two Per Protocol-analyses excluding those who did not join the SMS-program that they were offered; one accounting for a set of factors (maternal pregnancy healthy eating index score (low, medium, high), smoking in pregnancy (yes, no), physical activity level in pregnancy (low, medium, high metabolic equivalents (METS) score), pre-pregnancy BMI (underweight, normal weight, overweight, obese), and participation in the following previous DNBC follow-up surveys: when the child was 6 m, 18 m and 7 years, respectively (‘yes’ to all vs. at least one ‘no’)) that may influence participation (orange) and one not accounting for these factors (blue). Values presented by a dot show data at 6 months follow-up whereas values presented by a triangle show data at 18 months follow-up. IP: Inverse Probability, 95% CI: 95% Confidence Interval, SSB: Sugar sweetened beverages, FV: Fruits and vegetables, BMI: Body Mass Index, HEI: Healthy Eating Index, DNBC: Danish National Birth Cohort, y: Years, m [file pmed.1004383.s009.tif]

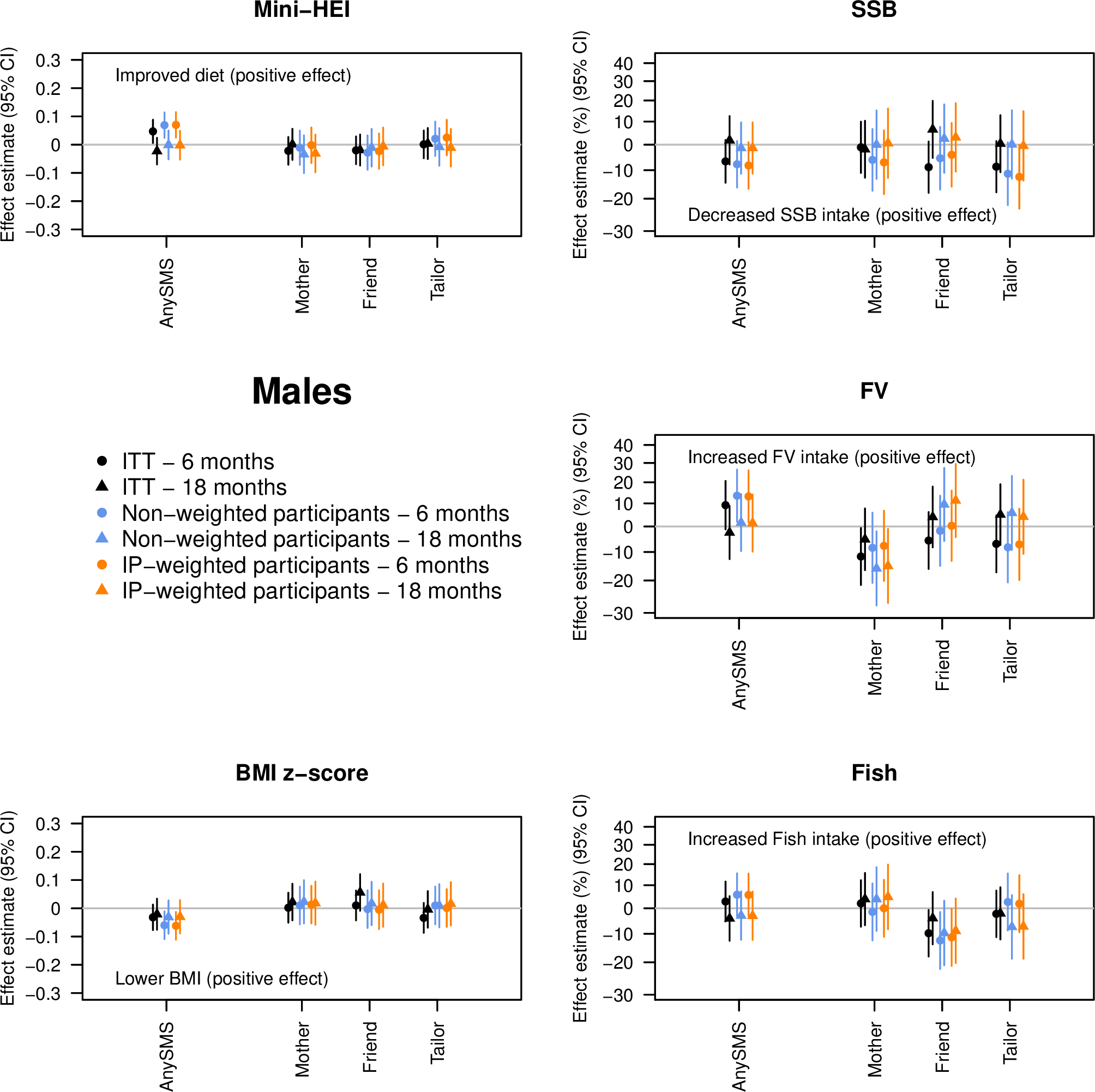

Supplement: S4 Fig — Analyses of the two main outcomes, mini-HEI and BMI z-score, are shown in the upper and lower left panel, respectively, on each page; whereas analyses of the secondary outcomes, SSB, FV and Fish z-scores, are shown in the upper, middle and lower right panel, respectively. In each panel are shown analyses of the effect estimates for the continuous outcomes performed for AnySMS compared to Non-SMS (the estimate to the left in each panel); and for each of the three additional elements, i.e. adding mother compared to not adding mother, adding friend compared to not adding friend, or tailored SMS program compared to the full SMS program, assessed within the group of AnySMS, thus excluding the Non-SMS group (the three estimates to the right in each panel, respectively). Effect sizes are estimated differences between comparison groups in means of outcomes at 6 months and 18 months follow-up. Colors indicate effects estimated from intention-to-treat (ITT)-analyses including all individuals (black) and two Per Protocol-analyses excluding those who did not join the SMS-program that they were offered; one accounting for a set of factors (maternal pregnancy healthy eating index score (low, medium, high), smoking in pregnancy (yes, no), physical activity level in pregnancy (low, medium, high metabolic equivalents (METS) score), pre-pregnancy BMI (underweight, normal weight, overweight, obese), and participation in the following previous DNBC follow-up surveys: when the child was 6 m, 18 m and 7 years, respectively (‘yes’ to all vs. at least one ‘no’)) that may influence participation (orange) and one not accounting for these factors (blue). Values presented by a dot show data at 6 months follow-up whereas values presented by a triangle show data at 18 months follow-up. IP: Inverse Probability, 95% CI: 95% Confidence Interval, SSB: Sugar sweetened beverages, FV: Fruits and vegetables, BMI: Body Mass Index, HEI: Healthy Eating Index, DNBC: Danish National Birth Cohort, y: Years, m [file pmed.1004383.s010.tif]

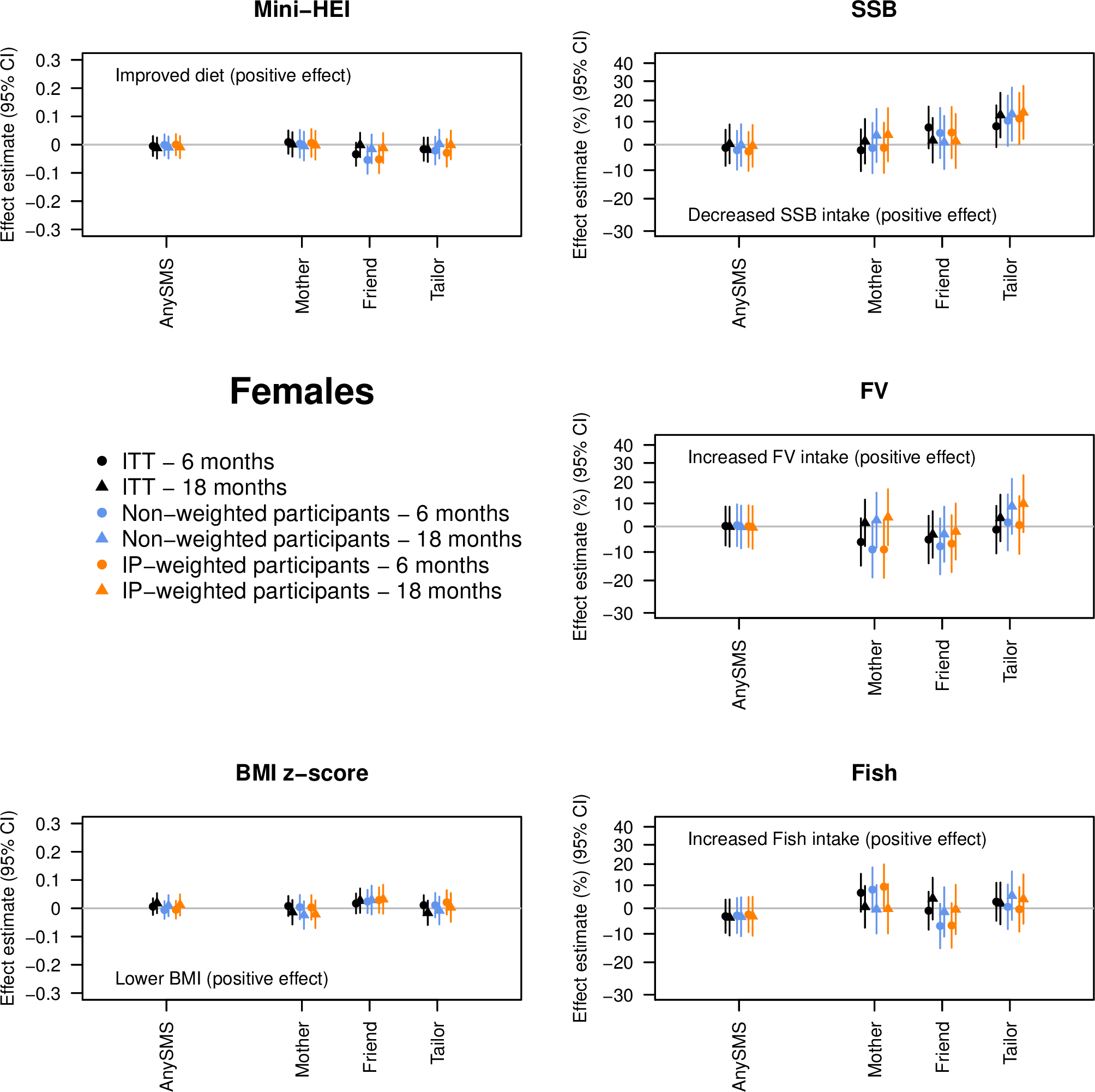

Supplement: S5 Fig — Analyses of the two main outcomes, mini-HEI and BMI z-score, are shown in the upper and lower left panel, respectively, on each page; whereas analyses of the secondary outcomes, SSB, FV and Fish z-scores, are shown in the upper, middle and lower right panel, respectively. In each panel are shown analyses of the effect estimates for the continuous outcomes performed for AnySMS compared to Non-SMS (the estimate to the left in each panel); and for each of the three additional elements, i.e. adding mother compared to not adding mother, adding friend compared to not adding friend, or tailored SMS program compared to the full SMS program, assessed within the group of AnySMS, thus excluding the Non-SMS group (the three estimates to the right in each panel, respectively). Effect sizes are estimated differences between comparison groups in means of outcomes at 6 months and 18 months follow-up. Colors indicate effects estimated from intention-to-treat (ITT)-analyses including all individuals (black) and two Per Protocol-analyses excluding those who did not join the SMS-program that they were offered; one accounting for a set of factors (maternal pregnancy healthy eating index score (low, medium, high), smoking in pregnancy (yes, no), physical activity level in pregnancy (low, medium, high metabolic equivalents (METS) score), pre-pregnancy BMI (underweight, normal weight, overweight, obese), and participation in the following previous DNBC follow-up surveys: when the child was 6 m, 18 m and 7 years, respectively (‘yes’ to all vs. at least one ‘no’)) that may influence participation (orange) and one not accounting for these factors (blue). Values presented by a dot show data at 6 months follow-up whereas values presented by a triangle show data at 18 months follow-up. IP: Inverse Probability, 95% CI: 95% Confidence Interval, SSB: Sugar sweetened beverages, FV: Fruits and vegetables, BMI: Body Mass Index, HEI: Healthy Eating Index, DNBC: Danish National Birth Cohort, y: Years, m [file pmed.1004383.s011.tif]

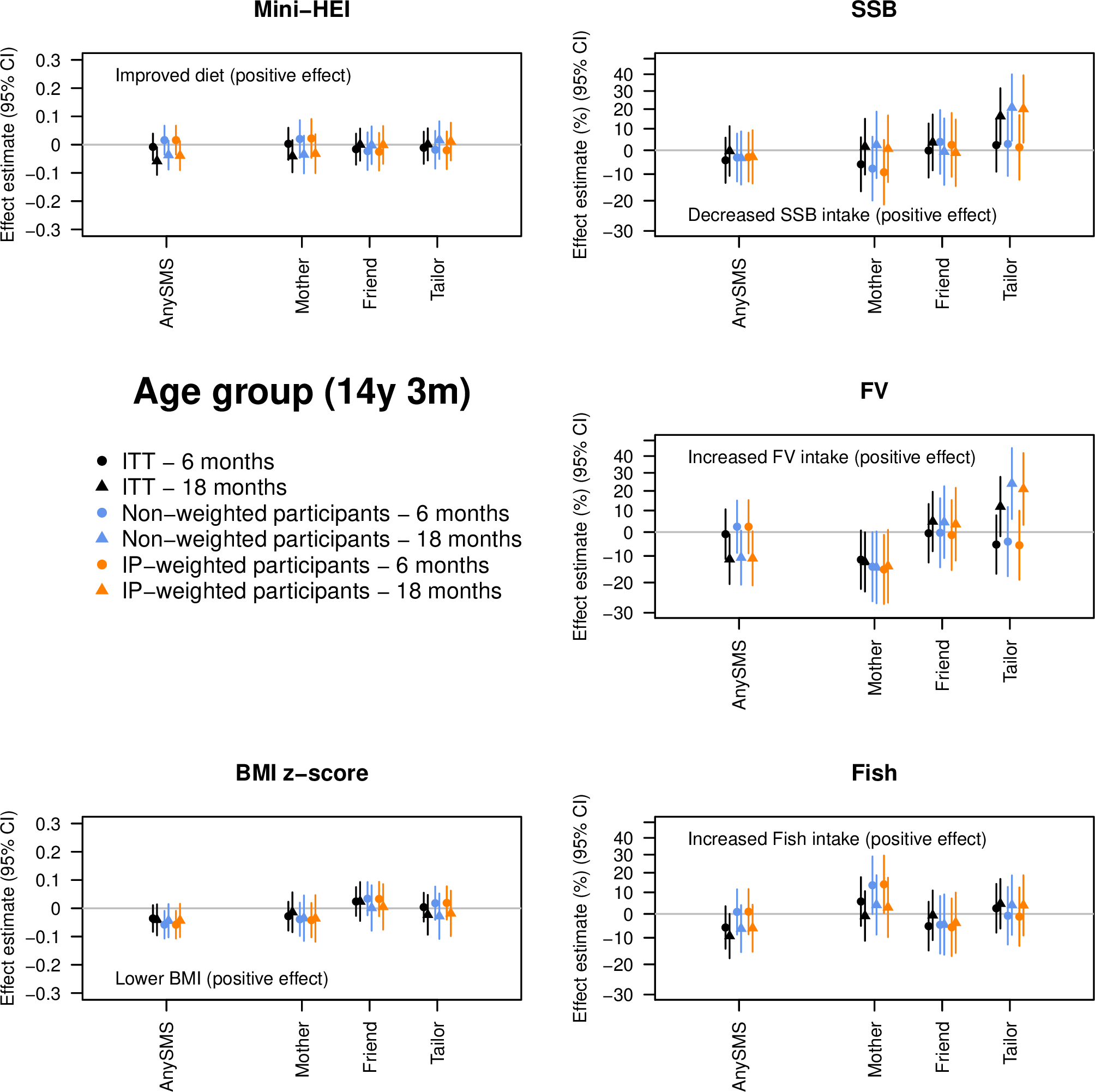

Supplement: S6 Fig — Analyses of the two main outcomes, mini-HEI and BMI z-score, are shown in the upper and lower left panel, respectively, on each page; whereas analyses of the secondary outcomes, SSB, FV and Fish z-scores, are shown in the upper, middle and lower right panel, respectively. In each panel are shown analyses of the effect estimates for the continuous outcomes performed for AnySMS compared to Non-SMS (the estimate to the left in each panel); and for each of the three additional elements, i.e. adding mother compared to not adding mother, adding friend compared to not adding friend, or tailored SMS program compared to the full SMS program, assessed within the group of AnySMS, thus excluding the Non-SMS group (the three estimates to the right in each panel, respectively). Effect sizes are estimated differences between comparison groups in means of outcomes at 6 months and 18 months follow-up. Colors indicate effects estimated from intention-to-treat (ITT)-analyses including all individuals (black) and two Per Protocol-analyses excluding those who did not join the SMS-program that they were offered; one accounting for a set of factors (maternal pregnancy healthy eating index score (low, medium, high), smoking in pregnancy (yes, no), physical activity level in pregnancy (low, medium, high metabolic equivalents (METS) score), pre-pregnancy BMI (underweight, normal weight, overweight, obese), and participation in the following previous DNBC follow-up surveys: when the child was 6 m, 18 m and 7 years, respectively (‘yes’ to all vs. at least one ‘no’)) that may influence participation (orange) and one not accounting for these factors (blue). Values presented by a dot show data at 6 months follow-up whereas values presented by a triangle show data at 18 months follow-up. IP: Inverse Probability, 95% CI: 95% Confidence Interval, SSB: Sugar sweetened beverages, FV: Fruits and vegetables, BMI: Body Mass Index, HEI: Healthy Eating Index, DNBC: Danish National Birth Cohort, y: Years, m [file pmed.1004383.s012.tif]

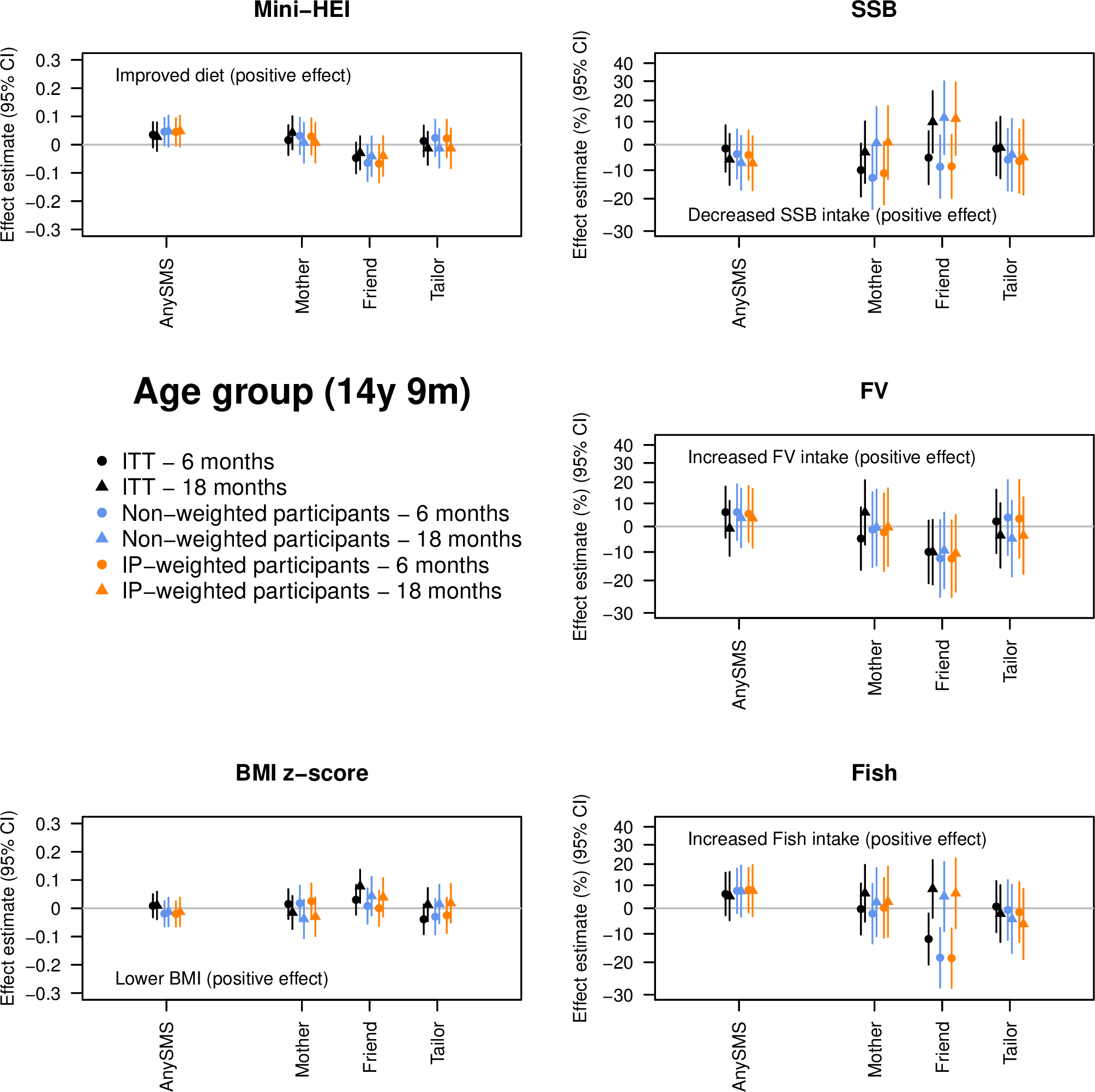

Supplement: S7 Fig — Analyses of the two main outcomes, mini-HEI and BMI z-score, are shown in the upper and lower left panel, respectively, on each page; whereas analyses of the secondary outcomes, SSB, FV and Fish z-scores, are shown in the upper, middle and lower right panel, respectively. In each panel are shown analyses of the effect estimates for the continuous outcomes performed for AnySMS compared to Non-SMS (the estimate to the left in each panel); and for each of the three additional elements, i.e. adding mother compared to not adding mother, adding friend compared to not adding friend, or tailored SMS program compared to the full SMS program, assessed within the group of AnySMS, thus excluding the Non-SMS group (the three estimates to the right in each panel, respectively). Effect sizes are estimated differences between comparison groups in means of outcomes at 6 months and 18 months follow-up. Colors indicate effects estimated from intention-to-treat (ITT)-analyses including all individuals (black) and two Per Protocol-analyses excluding those who did not join the SMS-program that they were offered; one accounting for a set of factors (maternal pregnancy healthy eating index score (low, medium, high), smoking in pregnancy (yes, no), physical activity level in pregnancy (low, medium, high metabolic equivalents (METS) score), pre-pregnancy BMI (underweight, normal weight, overweight, obese), and participation in the following previous DNBC follow-up surveys: when the child was 6 m, 18 m and 7 years, respectively (‘yes’ to all vs. at least one ‘no’)) that may influence participation (orange) and one not accounting for these factors (blue). Values presented by a dot show data at 6 months follow-up whereas values presented by a triangle show data at 18 months follow-up. IP: Inverse Probability, 95% CI: 95% Confidence Interval, SSB: Sugar sweetened beverages, FV: Fruits and vegetables, BMI: Body Mass Index, HEI: Healthy Eating Index, DNBC: Danish National Birth Cohort, y: Years, m [file pmed.1004383.s013.tif]

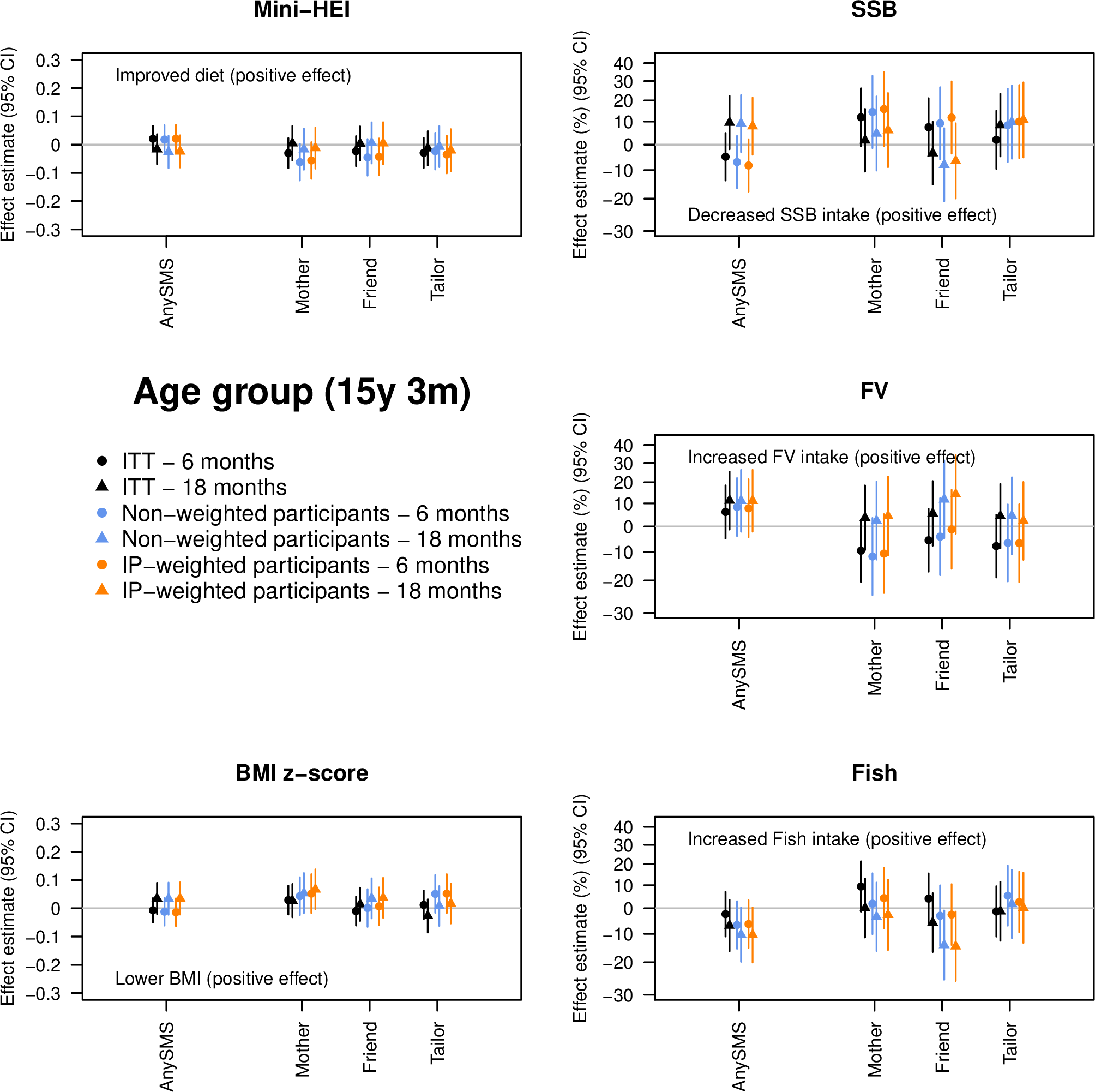

Supplement: S8 Fig — Analyses of the two main outcomes, mini-HEI and BMI z-score, are shown in the upper and lower left panel, respectively, on each page; whereas analyses of the secondary outcomes, SSB, FV and Fish z-scores, are shown in the upper, middle and lower right panel, respectively. In each panel are shown analyses of the effect estimates for the continuous outcomes performed for AnySMS compared to Non-SMS (the estimate to the left in each panel); and for each of the three additional elements, i.e. adding mother compared to not adding mother, adding friend compared to not adding friend, or tailored SMS program compared to the full SMS program, assessed within the group of AnySMS, thus excluding the Non-SMS group (the three estimates to the right in each panel, respectively). Effect sizes are estimated differences between comparison groups in means of outcomes at 6 months and 18 months follow-up. Colors indicate effects estimated from intention-to-treat (ITT)-analyses including all individuals (black) and two Per Protocol-analyses excluding those who did not join the SMS-program that they were offered; one accounting for a set of factors (maternal pregnancy healthy eating index score (low, medium, high), smoking in pregnancy (yes, no), physical activity level in pregnancy (low, medium, high metabolic equivalents (METS) score), pre-pregnancy BMI (underweight, normal weight, overweight, obese), and participation in the following previous DNBC follow-up surveys: when the child was 6 m, 18 m and 7 years, respectively (‘yes’ to all vs. at least one ‘no’)) that may influence participation (orange) and one not accounting for these factors (blue). Values presented by a dot show data at 6 months follow-up whereas values presented by a triangle show data at 18 months follow-up. IP: Inverse Probability, 95% CI: 95% Confidence Interval, SSB: Sugar sweetened beverages, FV: Fruits and vegetables, BMI: Body Mass Index, HEI: Healthy Eating Index, DNBC: Danish National Birth Cohort, y: Years, m [file pmed.1004383.s014.tif]

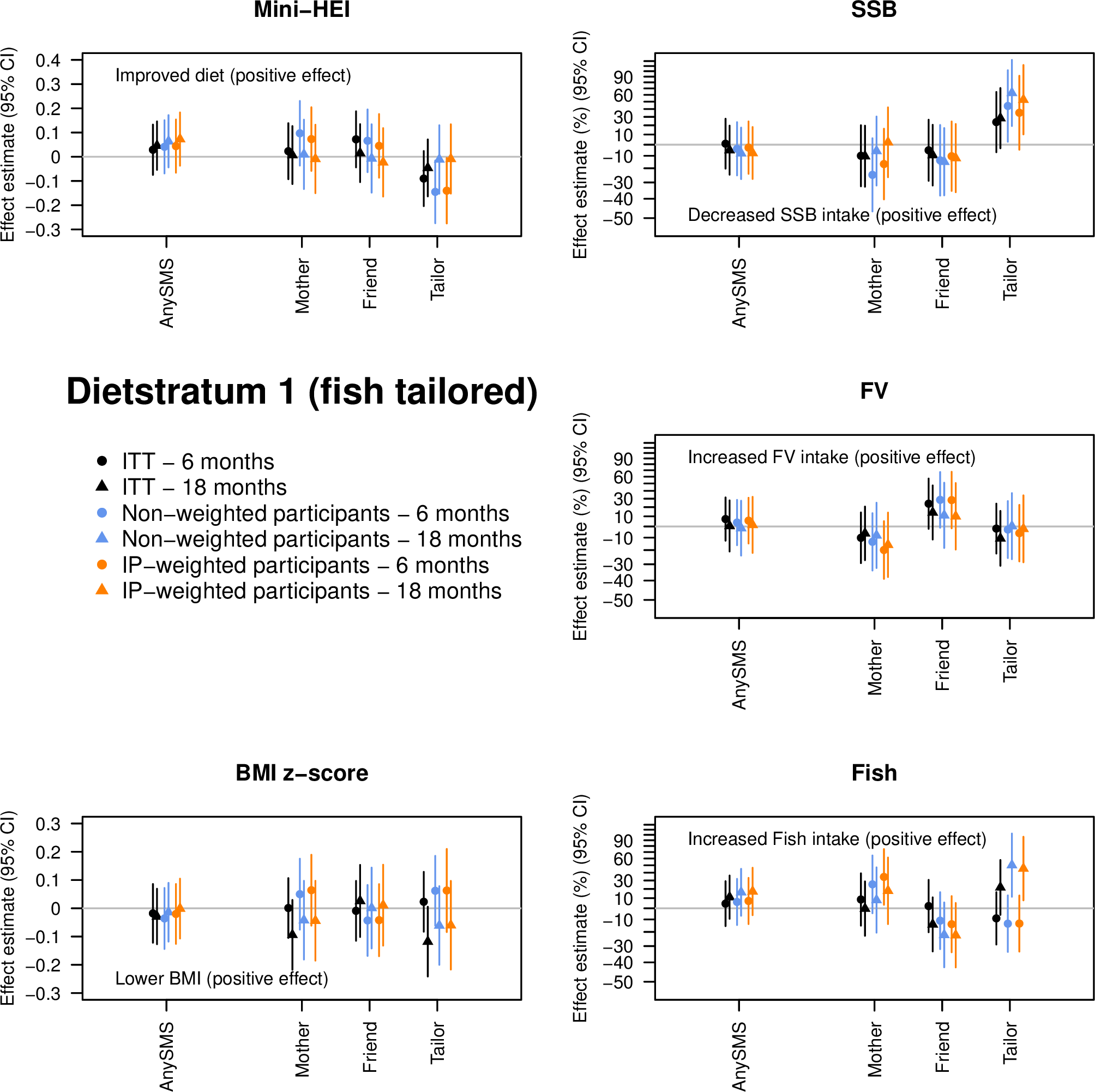

Supplement: S9 Fig — Analyses of the two main outcomes, mini-HEI and BMI z-score, are shown in the upper and lower left panel, respectively, on each page; whereas analyses of the secondary outcomes, SSB, FV and Fish z-scores, are shown in the upper, middle and lower right panel, respectively. In each panel are shown analyses of the effect estimates for the continuous outcomes performed for AnySMS compared to Non-SMS (the estimate to the left in each panel); and for each of the three additional elements, i.e. adding mother compared to not adding mother, adding friend compared to not adding friend, or tailored SMS program compared to the full SMS program, assessed within the group of AnySMS, thus excluding the Non-SMS group (the three estimates to the right in each panel, respectively). Effect sizes are estimated differences between comparison groups in means of outcomes at 6 months and 18 months follow-up. Colors indicate effects estimated from intention-to-treat (ITT)-analyses including all individuals (black) and two Per Protocol-analyses excluding those who did not join the SMS-program that they were offered; one accounting for a set of factors (maternal pregnancy healthy eating index score (low, medium, high), smoking in pregnancy (yes, no), physical activity level in pregnancy (low, medium, high metabolic equivalents (METS) score), pre-pregnancy BMI (underweight, normal weight, overweight, obese), and participation in the following previous DNBC follow-up surveys: when the child was 6 m, 18 m and 7 years, respectively (‘yes’ to all vs. at least one ‘no’)) that may influence participation (orange) and one not accounting for these factors (blue). Values presented by a dot show data at 6 months follow-up whereas values presented by a triangle show data at 18 months follow-up. IP: Inverse Probability, 95% CI: 95% Confidence Interval, SSB: Sugar sweetened beverages, FV: Fruits and vegetables, BMI: Body Mass Index, HEI: Healthy Eating Index, DNBC: Danish National Birth Cohort, y: Years, m [file pmed.1004383.s015.tif]

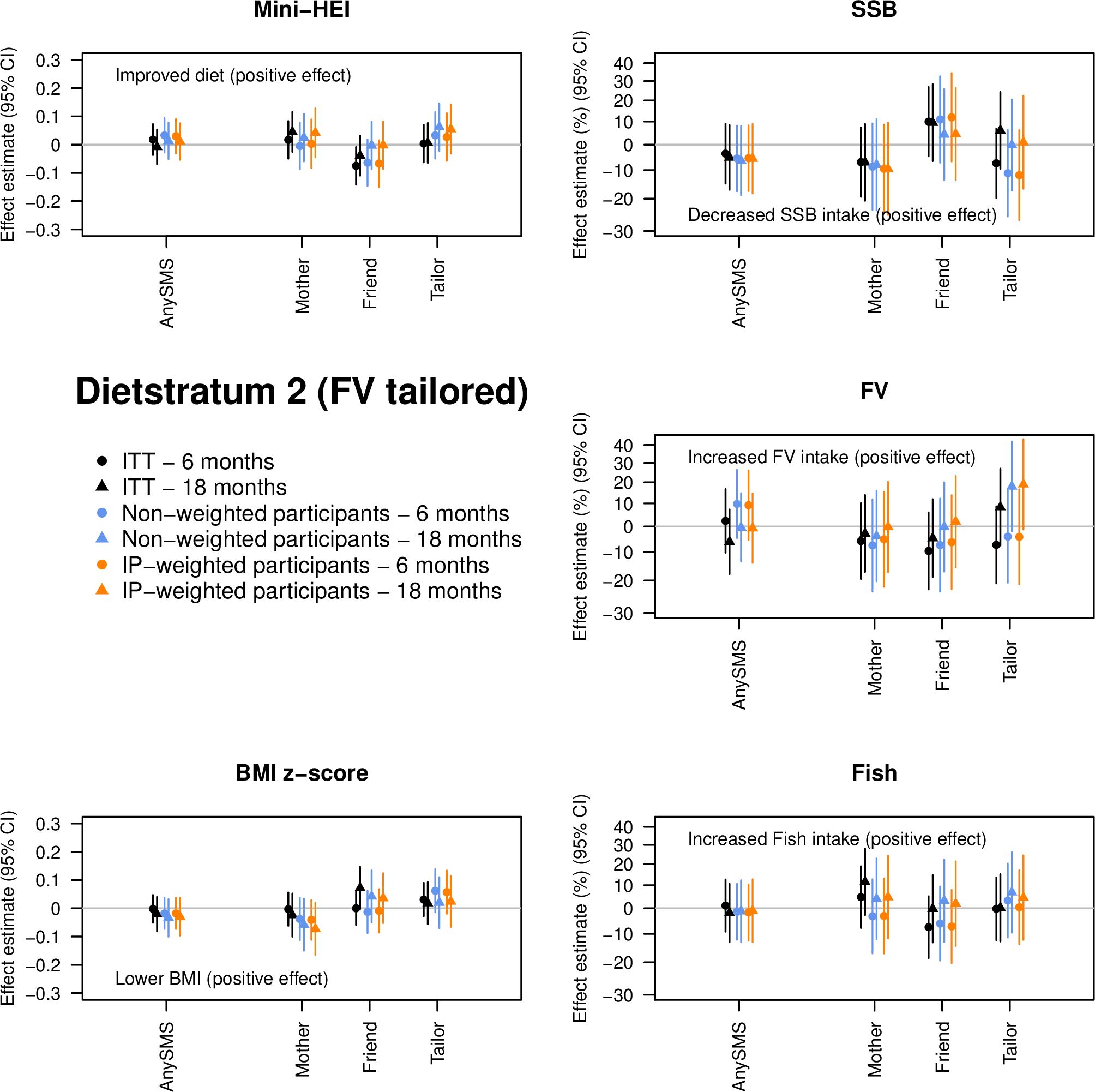

Supplement: S10 Fig — Analyses of the two main outcomes, mini-HEI and BMI z-score, are shown in the upper and lower left panel, respectively, on each page; whereas analyses of the secondary outcomes, SSB, FV and Fish z-scores, are shown in the upper, middle and lower right panel, respectively. In each panel are shown analyses of the effect estimates for the continuous outcomes performed for AnySMS compared to Non-SMS (the estimate to the left in each panel); and for each of the three additional elements, i.e. adding mother compared to not adding mother, adding friend compared to not adding friend, or tailored SMS program compared to the full SMS program, assessed within the group of AnySMS, thus excluding the Non-SMS group (the three estimates to the right in each panel, respectively). Effect sizes are estimated differences between comparison groups in means of outcomes at 6 months and 18 months follow-up. Colors indicate effects estimated from intention-to-treat (ITT)-analyses including all individuals (black) and two Per Protocol-analyses excluding those who did not join the SMS-program that they were offered; one accounting for a set of factors (maternal pregnancy healthy eating index score (low, medium, high), smoking in pregnancy (yes, no), physical activity level in pregnancy (low, medium, high metabolic equivalents (METS) score), pre-pregnancy BMI (underweight, normal weight, overweight, obese), and participation in the following previous DNBC follow-up surveys: when the child was 6 m, 18 m and 7 years, respectively (‘yes’ to all vs. at least one ‘no’)) that may influence participation (orange) and one not accounting for these factors (blue). Values presented by a dot show data at 6 months follow-up whereas values presented by a triangle show data at 18 months follow-up. IP: Inverse Probability, 95% CI: 95% Confidence Interval, SSB: Sugar sweetened beverages, FV: Fruits and vegetables, BMI: Body Mass Index, HEI: Healthy Eating Index, DNBC: Danish National Birth Cohort, y: Years, m [file pmed.1004383.s016.tif]

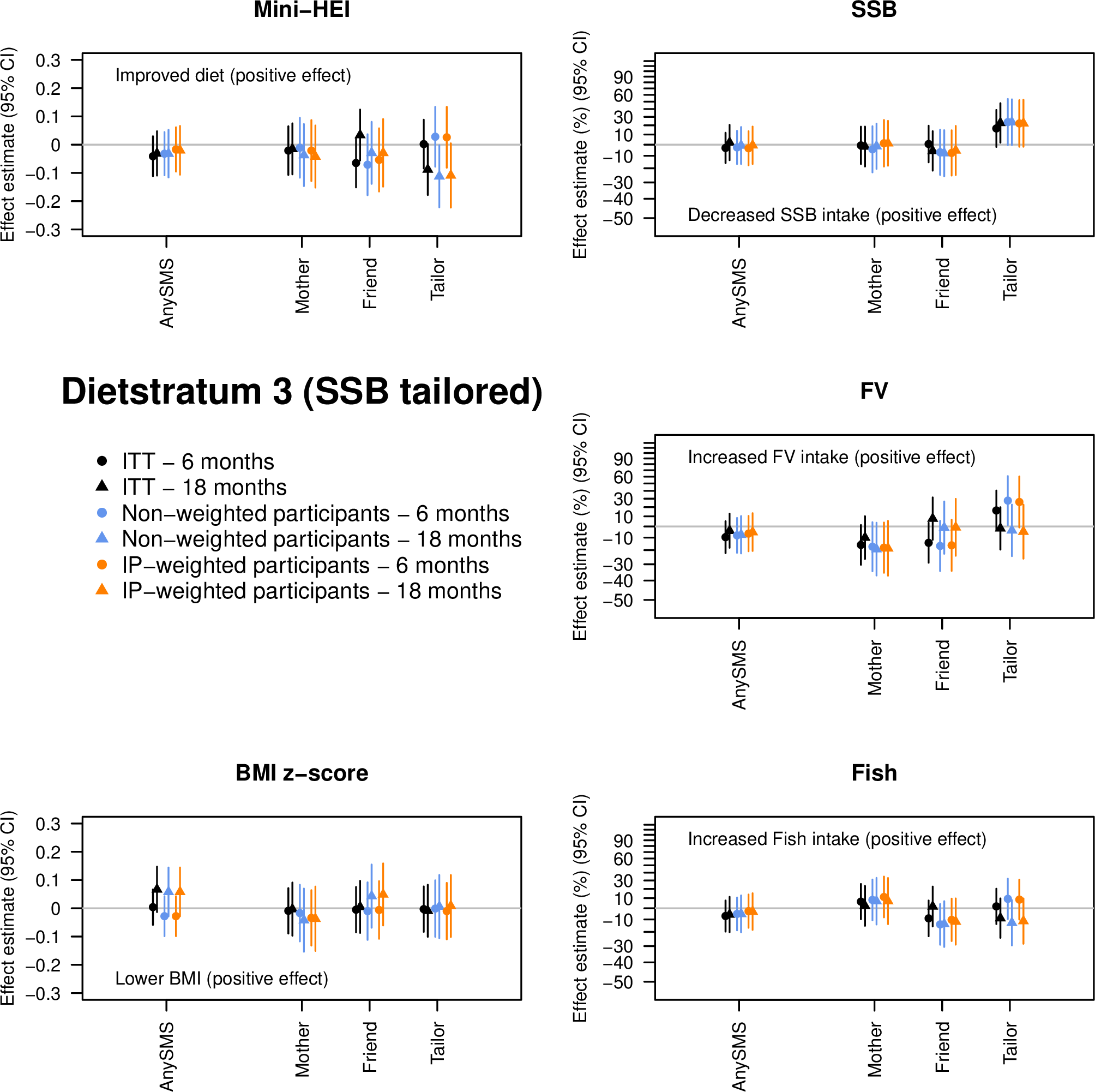

Supplement: S11 Fig — Analyses of the two main outcomes, mini-HEI and BMI z-score, are shown in the upper and lower left panel, respectively, on each page; whereas analyses of the secondary outcomes, SSB, FV and Fish z-scores, are shown in the upper, middle and lower right panel, respectively. In each panel are shown analyses of the effect estimates for the continuous outcomes performed for AnySMS compared to Non-SMS (the estimate to the left in each panel); and for each of the three additional elements, i.e. adding mother compared to not adding mother, adding friend compared to not adding friend, or tailored SMS program compared to the full SMS program, assessed within the group of AnySMS, thus excluding the Non-SMS group (the three estimates to the right in each panel, respectively). Effect sizes are estimated differences between comparison groups in means of outcomes at 6 months and 18 months follow-up. Colors indicate effects estimated from intention-to-treat (ITT)-analyses including all individuals (black) and two Per Protocol-analyses excluding those who did not join the SMS-program that they were offered; one accounting for a set of factors (maternal pregnancy healthy eating index score (low, medium, high), smoking in pregnancy (yes, no), physical activity level in pregnancy (low, medium, high metabolic equivalents (METS) score), pre-pregnancy BMI (underweight, normal weight, overweight, obese), and participation in the following previous DNBC follow-up surveys: when the child was 6 m, 18 m and 7 years, respectively (‘yes’ to all vs. at least one ‘no’)) that may influence participation (orange) and one not accounting for these factors (blue). Values presented by a dot show data at 6 months follow-up whereas values presented by a triangle show data at 18 months follow-up. IP: Inverse Probability, 95% CI: 95% Confidence Interval, SSB: Sugar sweetened beverages, FV: Fruits and vegetables, BMI: Body Mass Index, HEI: Healthy Eating Index, DNBC: Danish National Birth Cohort, y: Years, m [file pmed.1004383.s017.tif]

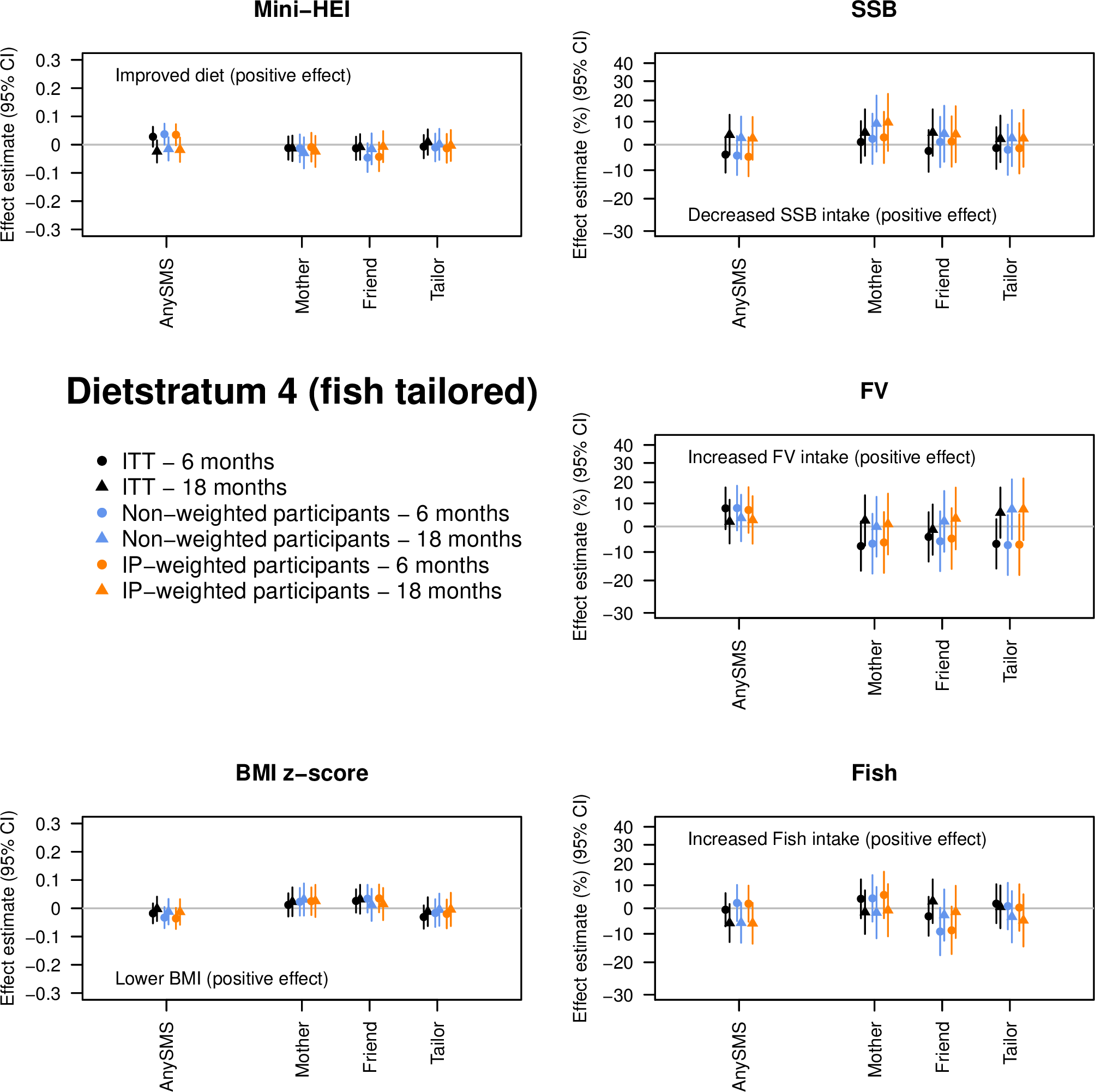

Supplement: S12 Fig — Analyses of the two main outcomes, mini-HEI and BMI z-score, are shown in the upper and lower left panel, respectively, on each page; whereas analyses of the secondary outcomes, SSB, FV and Fish z-scores, are shown in the upper, middle and lower right panel, respectively. In each panel are shown analyses of the effect estimates for the continuous outcomes performed for AnySMS compared to Non-SMS (the estimate to the left in each panel); and for each of the three additional elements, i.e. adding mother compared to not adding mother, adding friend compared to not adding friend, or tailored SMS program compared to the full SMS program, assessed within the group of AnySMS, thus excluding the Non-SMS group (the three estimates to the right in each panel, respectively). Effect sizes are estimated differences between comparison groups in means of outcomes at 6 months and 18 months follow-up. Colors indicate effects estimated from intention-to-treat (ITT)-analyses including all individuals (black) and two Per Protocol-analyses excluding those who did not join the SMS-program that they were offered; one accounting for a set of factors (maternal pregnancy healthy eating index score (low, medium, high), smoking in pregnancy (yes, no), physical activity level in pregnancy (low, medium, high metabolic equivalents (METS) score), pre-pregnancy BMI (underweight, normal weight, overweight, obese), and participation in the following previous DNBC follow-up surveys: when the child was 6 m, 18 m and 7 years, respectively (‘yes’ to all vs. at least one ‘no’)) that may influence participation (orange) and one not accounting for these factors (blue). Values presented by a dot show data at 6 months follow-up whereas values presented by a triangle show data at 18 months follow-up. IP: Inverse Probability, 95% CI: 95% Confidence Interval, SSB: Sugar sweetened beverages, FV: Fruits and vegetables, BMI: Body Mass Index, HEI: Healthy Eating Index, DNBC: Danish National Birth Cohort, y: Years, m [file pmed.1004383.s018.tif]

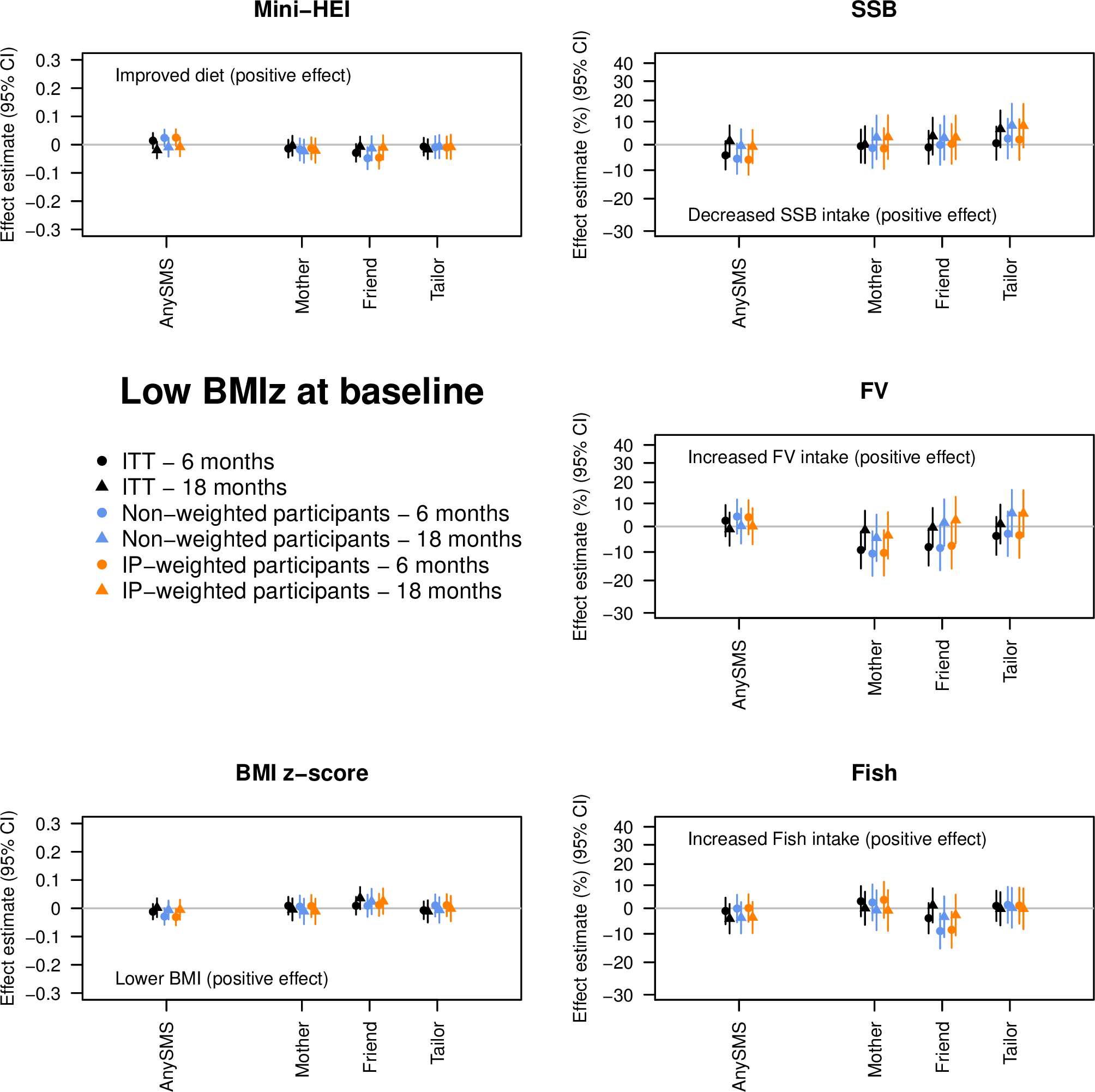

Supplement: S13 Fig — Analyses of the two main outcomes, mini-HEI and BMI z-score, are shown in the upper and lower left panel, respectively, on each page; whereas analyses of the secondary outcomes, SSB, FV and Fish z-scores, are shown in the upper, middle and lower right panel, respectively. In each panel are shown analyses of the effect estimates for the continuous outcomes performed for AnySMS compared to Non-SMS (the estimate to the left in each panel); and for each of the three additional elements, i.e. adding mother compared to not adding mother, adding friend compared to not adding friend, or tailored SMS program compared to the full SMS program, assessed within the group of AnySMS, thus excluding the Non-SMS group (the three estimates to the right in each panel, respectively). Effect sizes are estimated differences between comparison groups in means of outcomes at 6 months and 18 months follow-up. Colors indicate effects estimated from intention-to-treat (ITT)-analyses including all individuals (black) and two Per Protocol-analyses excluding those who did not join the SMS-program that they were offered; one accounting for a set of factors (maternal pregnancy healthy eating index score (low, medium, high), smoking in pregnancy (yes, no), physical activity level in pregnancy (low, medium, high metabolic equivalents (METS) score), pre-pregnancy BMI (underweight, normal weight, overweight, obese), and participation in the following previous DNBC follow-up surveys: when the child was 6 m, 18 m and 7 years, respectively (‘yes’ to all vs. at least one ‘no’)) that may influence participation (orange) and one not accounting for these factors (blue). Values presented by a dot show data at 6 months follow-up whereas values presented by a triangle show data at 18 months follow-up. IP: Inverse Probability, 95% CI: 95% Confidence Interval, SSB: Sugar sweetened beverages, FV: Fruits and vegetables, BMI: Body Mass Index, HEI: Healthy Eating Index, DNBC: Danish National Birth Cohort, y: Years, m [file pmed.1004383.s019.tif]

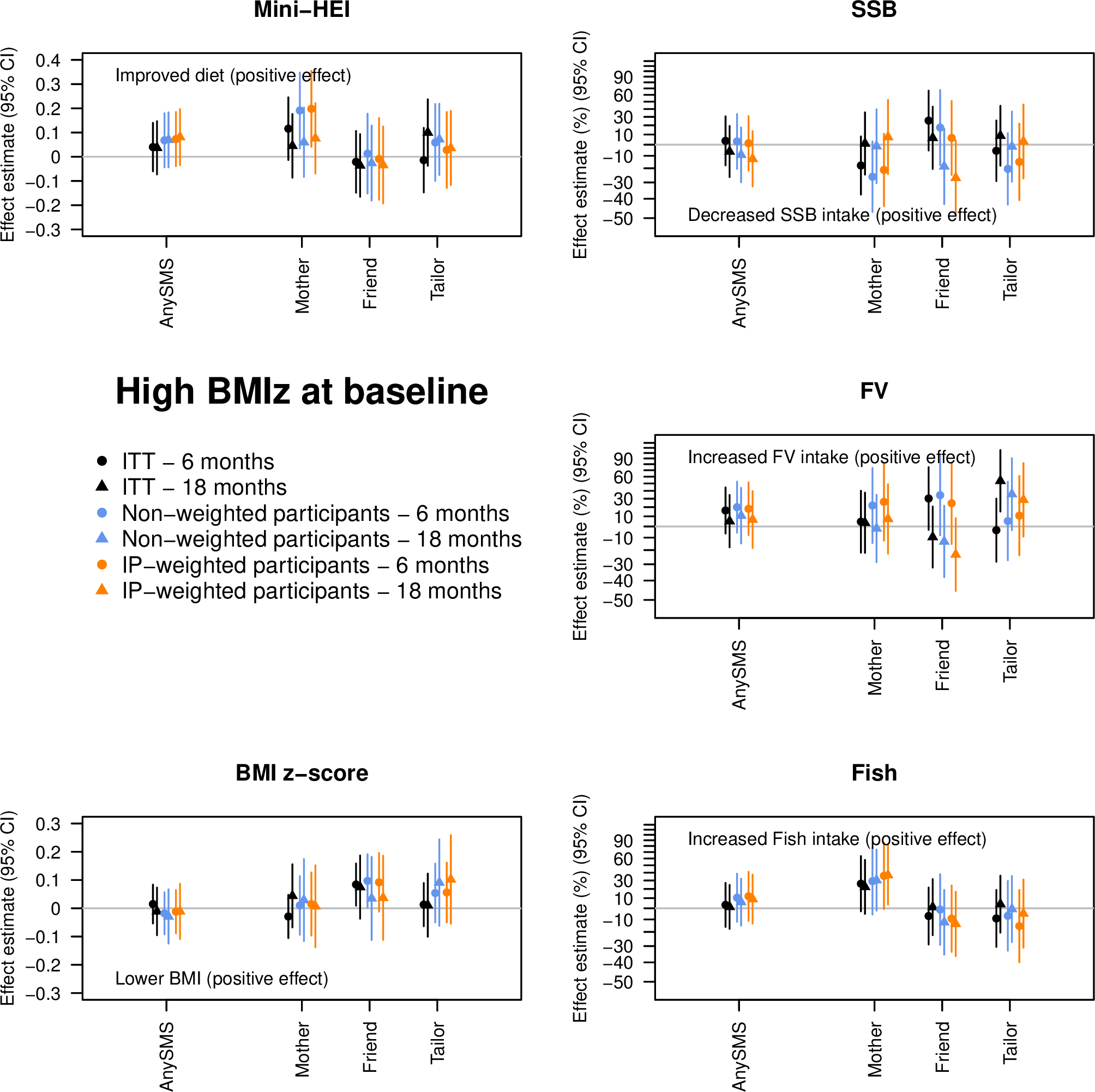

Supplement: S14 Fig — Analyses of the two main outcomes, mini-HEI and BMI z-score, are shown in the upper and lower left panel, respectively, on each page; whereas analyses of the secondary outcomes, SSB, FV and Fish z-scores, are shown in the upper, middle and lower right panel, respectively. In each panel are shown analyses of the effect estimates for the continuous outcomes performed for AnySMS compared to Non-SMS (the estimate to the left in each panel); and for each of the three additional elements, i.e. adding mother compared to not adding mother, adding friend compared to not adding friend, or tailored SMS program compared to the full SMS program, assessed within the group of AnySMS, thus excluding the Non-SMS group (the three estimates to the right in each panel, respectively). Effect sizes are estimated differences between comparison groups in means of outcomes at 6 months and 18 months follow-up. Colors indicate effects estimated from intention-to-treat (ITT)-analyses including all individuals (black) and two Per Protocol-analyses excluding those who did not join the SMS-program that they were offered; one accounting for a set of factors (maternal pregnancy healthy eating index score (low, medium, high), smoking in pregnancy (yes, no), physical activity level in pregnancy (low, medium, high metabolic equivalents (METS) score), pre-pregnancy BMI (underweight, normal weight, overweight, obese), and participation in the following previous DNBC follow-up surveys: when the child was 6 m, 18 m and 7 years, respectively (‘yes’ to all vs. at least one ‘no’)) that may influence participation (orange) and one not accounting for these factors (blue). Values presented by a dot show data at 6 months follow-up whereas values presented by a triangle show data at 18 months follow-up. IP: Inverse Probability, 95% CI: 95% Confidence Interval, SSB: Sugar sweetened beverages, FV: Fruits and vegetables, BMI: Body Mass Index, HEI: Healthy Eating Index, DNBC: Danish National Birth Cohort, y: Years, m [file pmed.1004383.s020.tif]

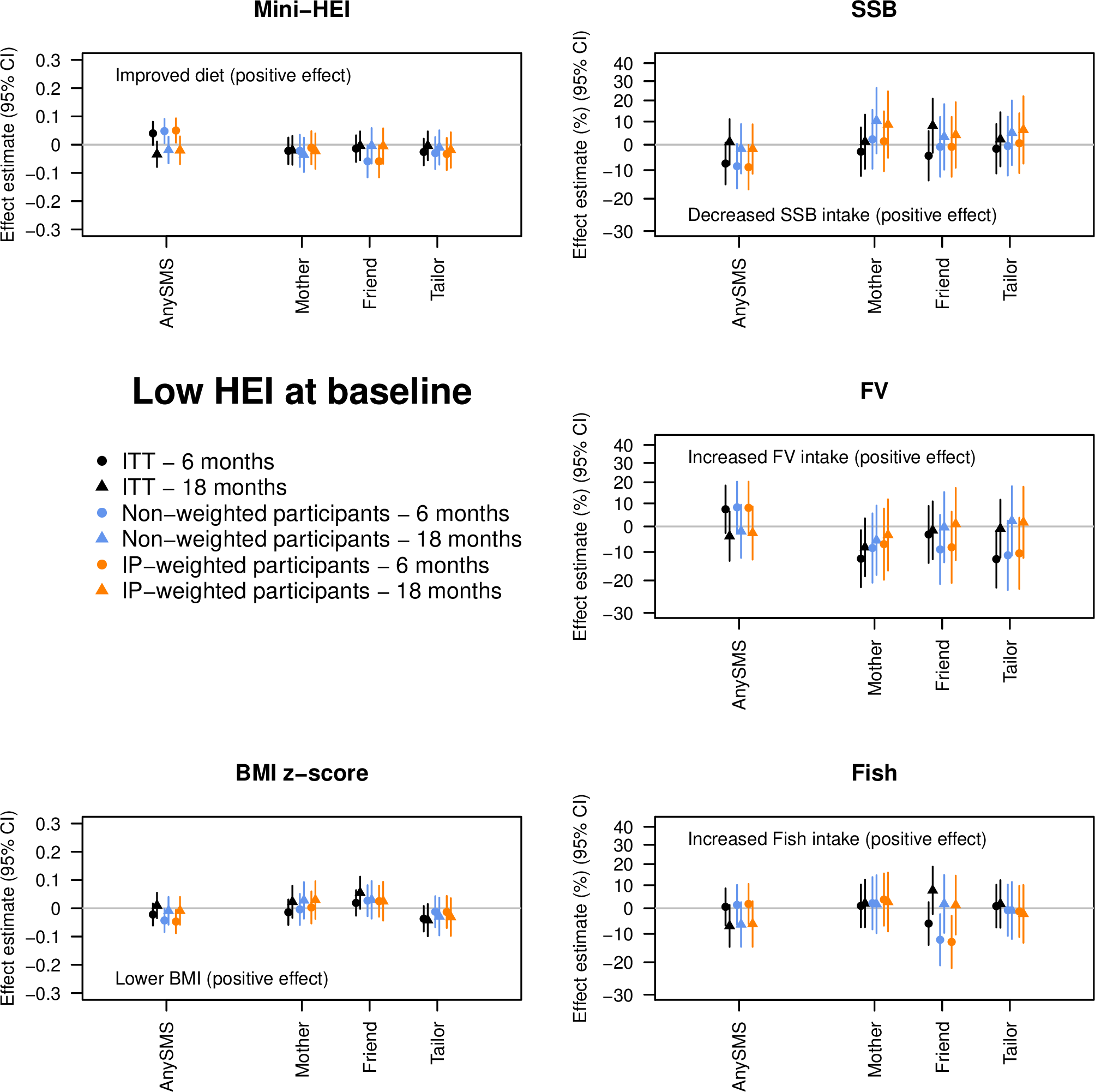

Supplement: S15 Fig — Analyses of the two main outcomes, mini-HEI and BMI z-score, are shown in the upper and lower left panel, respectively, on each page; whereas analyses of the secondary outcomes, SSB, FV and Fish z-scores, are shown in the upper, middle and lower right panel, respectively. In each panel are shown analyses of the effect estimates for the continuous outcomes performed for AnySMS compared to Non-SMS (the estimate to the left in each panel); and for each of the three additional elements, i.e. adding mother compared to not adding mother, adding friend compared to not adding friend, or tailored SMS program compared to the full SMS program, assessed within the group of AnySMS, thus excluding the Non-SMS group (the three estimates to the right in each panel, respectively). Effect sizes are estimated differences between comparison groups in means of outcomes at 6 months and 18 months follow-up. Colors indicate effects estimated from intention-to-treat (ITT)-analyses including all individuals (black) and two Per Protocol-analyses excluding those who did not join the SMS-program that they were offered; one accounting for a set of factors (maternal pregnancy healthy eating index score (low, medium, high), smoking in pregnancy (yes, no), physical activity level in pregnancy (low, medium, high metabolic equivalents (METS) score), pre-pregnancy BMI (underweight, normal weight, overweight, obese), and participation in the following previous DNBC follow-up surveys: when the child was 6 m, 18 m and 7 years, respectively (‘yes’ to all vs. at least one ‘no’)) that may influence participation (orange) and one not accounting for these factors (blue). Values presented by a dot show data at 6 months follow-up whereas values presented by a triangle show data at 18 months follow-up. IP: Inverse Probability, 95% CI: 95% Confidence Interval, SSB: Sugar sweetened beverages, FV: Fruits and vegetables, BMI: Body Mass Index, HEI: Healthy Eating Index, DNBC: Danish National Birth Cohort, y: Years, m [file pmed.1004383.s021.tif]

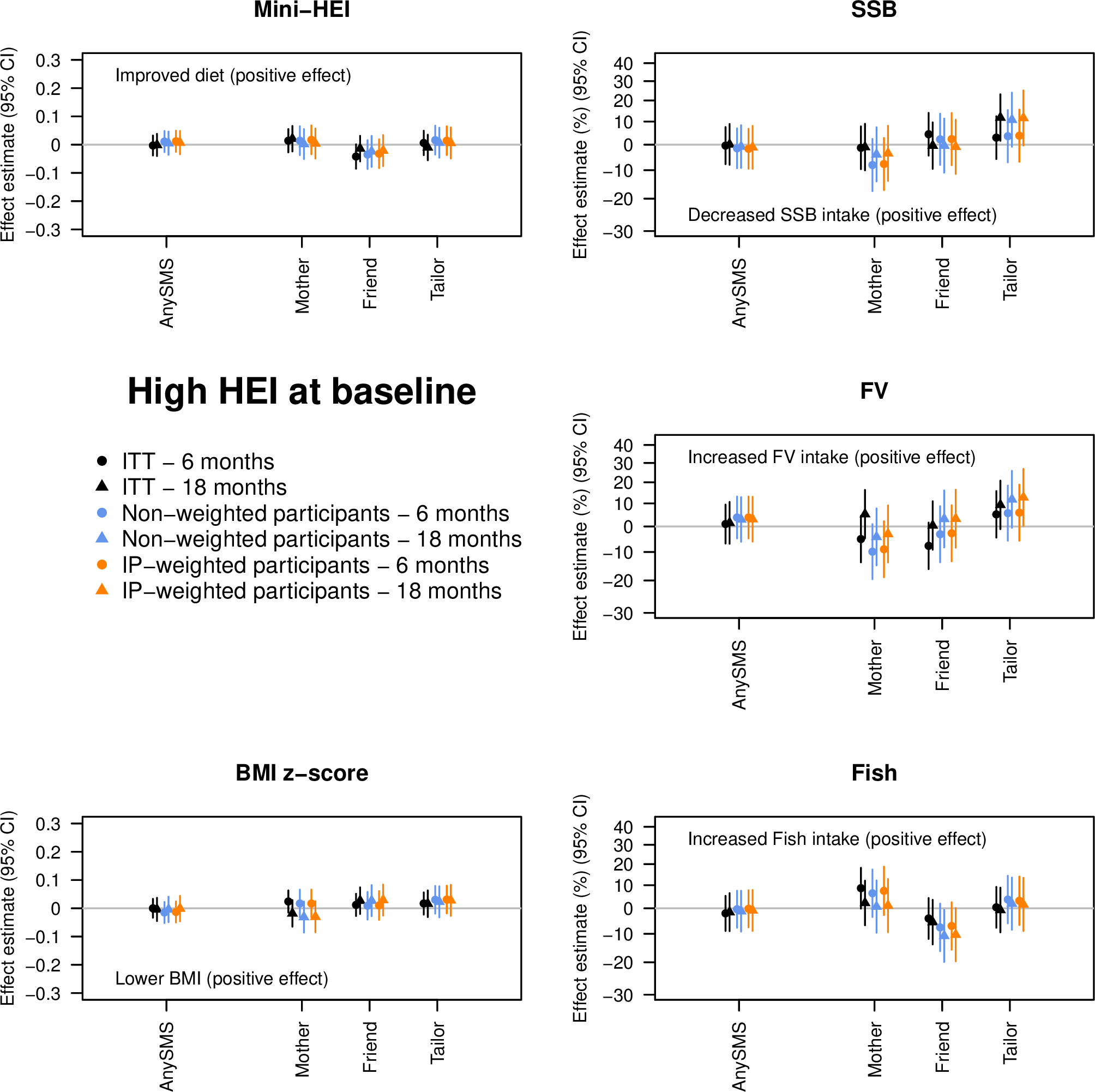

Supplement: S16 Fig — Analyses of the two main outcomes, mini-HEI and BMI z-score, are shown in the upper and lower left panel, respectively, on each page; whereas analyses of the secondary outcomes, SSB, FV and Fish z-scores, are shown in the upper, middle and lower right panel, respectively. In each panel are shown analyses of the effect estimates for the continuous outcomes performed for AnySMS compared to Non-SMS (the estimate to the left in each panel); and for each of the three additional elements, i.e. adding mother compared to not adding mother, adding friend compared to not adding friend, or tailored SMS program compared to the full SMS program, assessed within the group of AnySMS, thus excluding the Non-SMS group (the three estimates to the right in each panel, respectively). Effect sizes are estimated differences between comparison groups in means of outcomes at 6 months and 18 months follow-up. Colors indicate effects estimated from intention-to-treat (ITT)-analyses including all individuals (black) and two Per Protocol-analyses excluding those who did not join the SMS-program that they were offered; one accounting for a set of factors (maternal pregnancy healthy eating index score (low, medium, high), smoking in pregnancy (yes, no), physical activity level in pregnancy (low, medium, high metabolic equivalents (METS) score), pre-pregnancy BMI (underweight, normal weight, overweight, obese), and participation in the following previous DNBC follow-up surveys: when the child was 6 m, 18 m and 7 years, respectively (‘yes’ to all vs. at least one ‘no’)) that may influence participation (orange) and one not accounting for these factors (blue). Values presented by a dot show data at 6 months follow-up whereas values presented by a triangle show data at 18 months follow-up. IP: Inverse Probability, 95% CI: 95% Confidence Interval, SSB: Sugar sweetened beverages, FV: Fruits and vegetables, BMI: Body Mass Index, HEI: Healthy Eating Index, DNBC: Danish National Birth Cohort, y: Years, m [file pmed.1004383.s022.tif]
